# Supplementary material for: Plasma infrared fingerprinting with machine learning enables single-measurement multi-phenotype health screening
Source: Cell Rep Med. 2024 Jun 28;5(7):101625. doi: 10.1016/j.xcrm.2024.101625 (PMC11293328; doi:10.1016/j.xcrm.2024.101625)
Supplement: Document S1. Figures S1–S8 and Tables S1–S5 [file mmc1.pdf]

Cell Reports Medicine, Volume *5*

Supplemental information

Plasma infrared fingerprinting with machine learning enables single-measurement multi-phenotype health screening

Tarek Eissa, Cristina Leonardo, Kosmas V. Kepesidis, Frank Fleischmann, Birgit Linkohr, Daniel Meyer, Viola Zoka, Marinus Huber, Liudmila Voronina, Lothar Richter, Annette Peters, and Mihaela Žigman

	Sample set #1 (3044 samples)	Sample set #2 (2140 samples)	Combined (5184 samples)
**Male proportion, %**	48.23	48.79	48.46
**Age, mean (± SD),** ***years***	56.19 (±13.24)	60.19 (±12.30)	57.84 (±13.01)
**Body mass index, mean (± SD),** ***kg/m***^{2}	27.62 (±4.81)	27.77 (±4.92)	27.68 (±4.85)
**Total cholesterol, mean (± SD),** ***mmol/l***	5.58 (±1.02)	5.58 (±1.01)	5.58 (±1.02)
**LDL cholesterol, mean (± SD),** ***mmol/l***	3.51 (±0.90)	3.48 (±0.90)	3.50 (±0.90)
**HDL cholesterol, mean (± SD),** ***mmol/l***	1.45 (±0.37)	1.69 (±0.48)	1.55 (±0.44)
**Triglycerides, mean (± SD),** ***mmol/l***	1.42 (±0.97)	1.38 (±0.81)	1.40 (±0.90)
**Hematocrit, mean (± SD),** ***l/l***	0.41 (±0.03)	0.41 (±0.03)	0.41 (±0.03)
**Erythrocytes, mean (± SD),** ***/pl***	4.53 (±0.42)	4.60 (±0.43)	4.56 (±0.42)
**Leukocytes, mean (± SD),** ***/nl***	5.98 (±1.88)	5.98 (±1.66)	5.98 (±1.79)
**Platelets, mean (± SD),** ***/nl***	253.99 (±62.29)	229.52 (±55.84)	243.89 (±60.91)
**HbA1c, mean (± SD),** ***mmol/mol***	37.21 (±6.80)	37.05 (±6.99)	37.14 (±6.88)
**Fasting glucose, mean (± SD),** ***mmol/l***	5.46 (±1.08)	5.69 (±1.19)	5.55 (±1.13)
**Albumin, mean (± SD),** ***g/l***	44.57 (±3.38)	42.54 (±3.17)	43.73 (±3.44)
**Creatinine, mean (± SD),** μ***mol/l***	80.45 (±21.58)	80.57 (±20.67)	80.50 (±21.21)

Supplementary Table S1. Description of study cohort, related to STAR Methods. Two time-separated samplings of a longitudinal population-based cohort were considered.

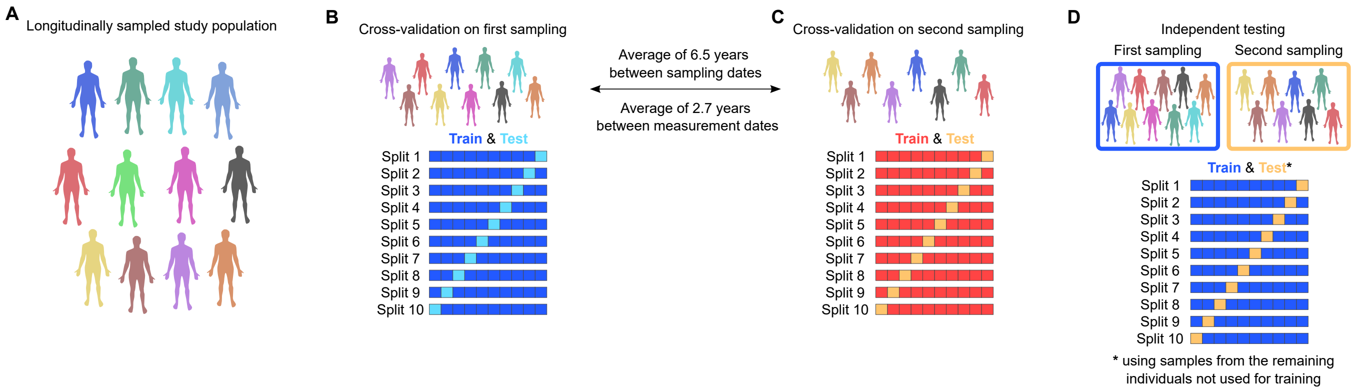

Supplementary Figure S1. Model validation procedure, related to STAR Methods. (**A**) Studied population consisting of naturally occurring heterogeneity between individuals that was sampled at two time points, thus longitudinal. (**B**) On the first sample set of the two, 10-fold cross-validation was performed to obtain metrics of model performance in 10 train and test splits of the samples. Each split was trained on approximately 90% of the samples and tested on approximately 10% of the remaining samples. (**C**) On the second sample set, the same cross-validation procedure was performed – independently of the first. (**D**) In 10 splits, model training was performed on 90% of the first sample set, leaving out a different set of individuals in each iteration. Model testing was then performed on the individuals from the second sample set who were not included in the training data from the first sample set for each data split. The model was thus tested on a dataset of independent samples and individuals.

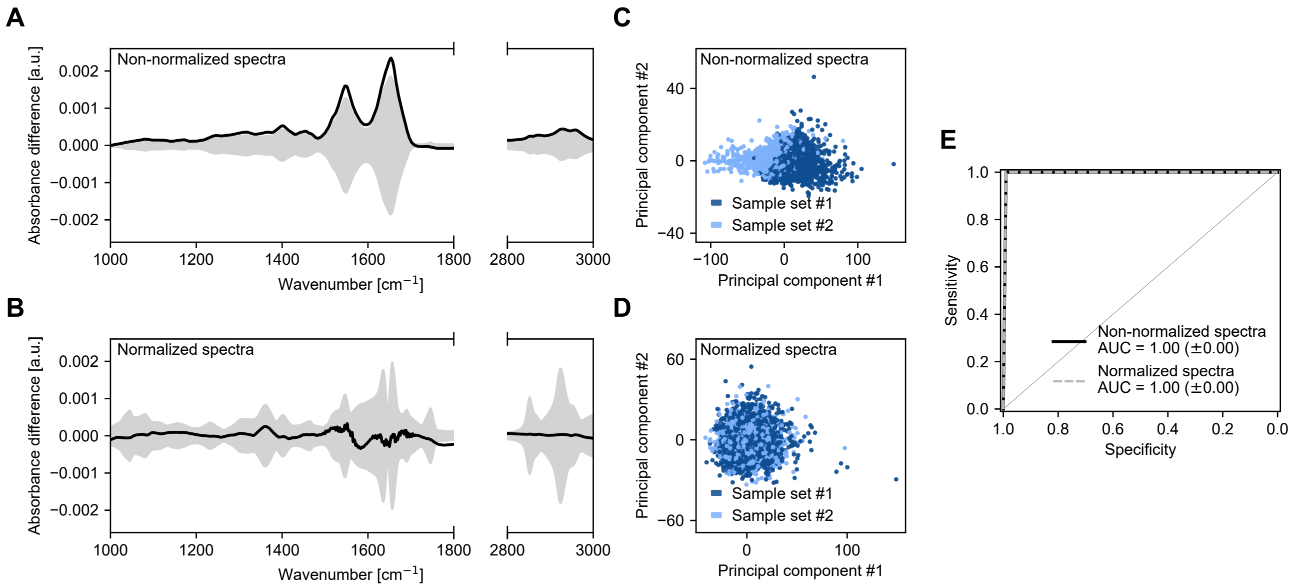

Supplementary Figure S2. Effects of variations in sample and measurement conditions on IR molecular fingerprints, related to STAR Methods. (**A**–**B**) Difference between the mean measurement of the first sample set and the mean measurement of the second on (**A**) non-normalized spectra and (**B**) normalized spectra. Gray-shaded areas depict the standard deviation of the IR fingerprints. (**C**–**D**) Principal component analysis was applied on the combined set of spectra from the two sample sets on (**C**) non-normalized spectra and (**D**) normalized spectra, color-coding the measurements from each sample set. (**E**) Binary classifications were carried out to determine to which sample set a measurement belonged to. Both sample sets were combined into one dataset, and the classifier was trained to assign each measurement into one sample set to determine whether there exists differences between the IR spectra of the sample sets. Two ROC curves of the classifications are depicted – one when applied to normalized spectra and one when applied to non-normalized spectra. The ROC curves are depicted on test samples as determined via cross-validation. Both classifiers were capable of perfectly determining to which sample set each measurement belonged to – revealing that measurement variations existed between the sample sets (even on normalized spectra), despite the overlapping cloud appearance depicted in (**D**).

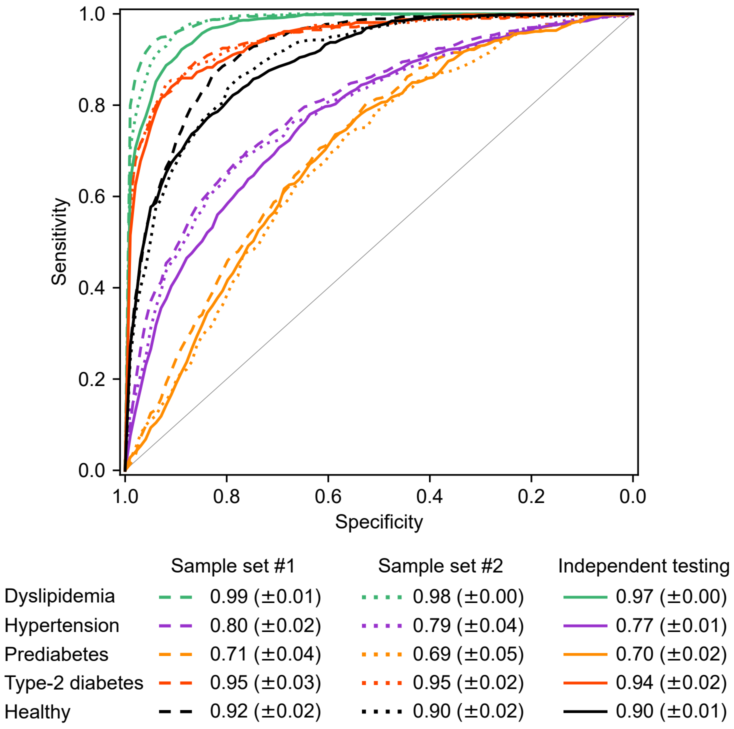

Supplementary Figure S3. Receiver operating characteristic (ROC) curves for binary classifications modeling each listed outcome against all other outcomes (one-versus-rest) without the classifier chaining, related to Figure 2. For each classification, the mean cross-validated ROC curve of the test sets is depicted and the area under the curve is listed below, along with its standard deviation. Model validation was carried out as described in the main text. Compared to the ROC curves depicted in Figure 2**D** in the main text, the binary classification analysis here was performed with independent binary classifiers that do not utilize the chain mechanism.

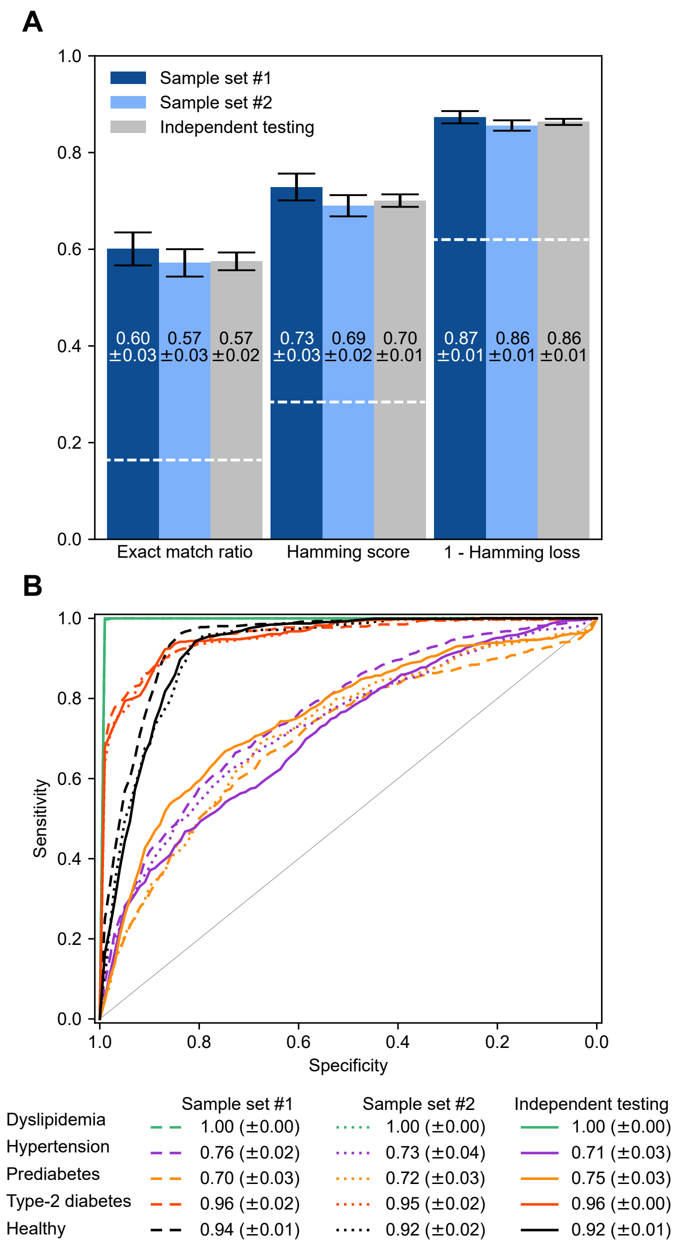

Supplementary Figure S4. Capacity of clinical analytes to predict common phenotypes, related to Figure 2. Twelve clinical analytes (total cholesterol, LDL cholesterol, HDL cholesterol, triglycerides, hematocrit, erythrocytes, leukocytes, platelets, HbA1c, fasting glucose, albumin, and creatinine) were used to compare the performance of simultaneously detecting the phenotypes of interest with the performance of using the IR molecular fingerprints as exclusive predictors (shown in Figure 2 in the main text). (**A**–**B**) Test predictive performance of a model using the clinical analytes as the exclusive predictors. Model validation was carried out as described in the main text.

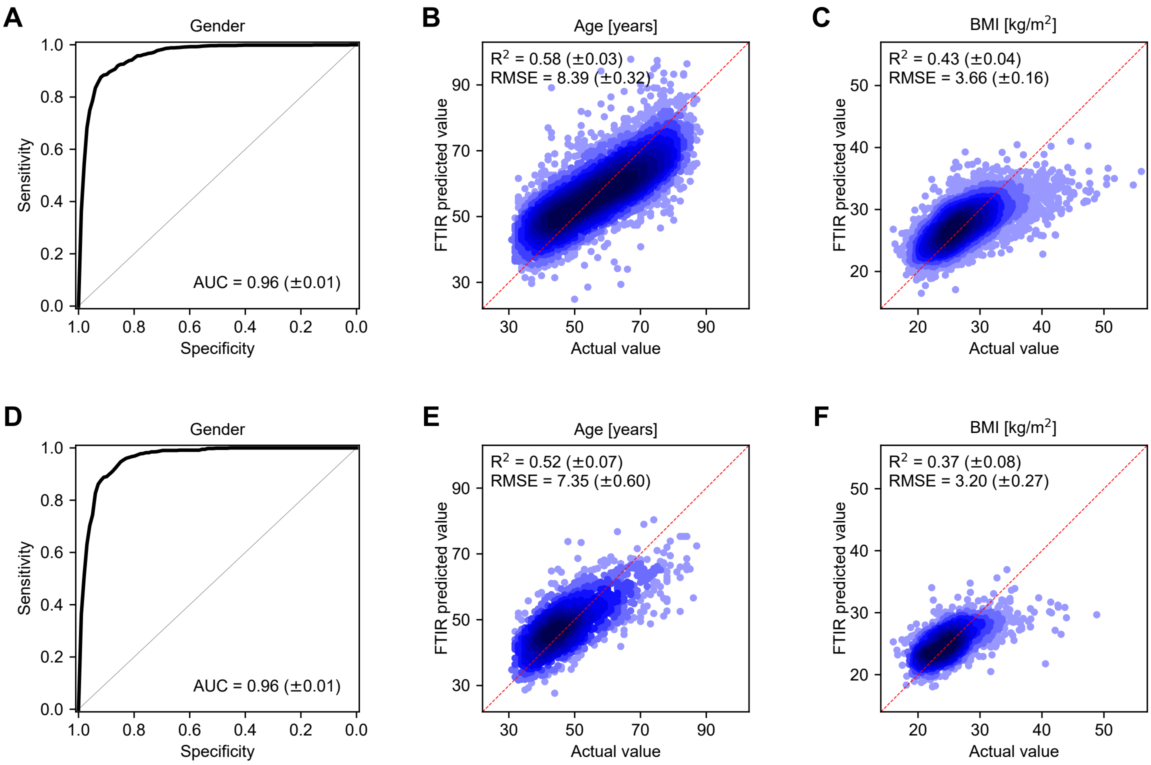

Supplementary Figure S5. Prediction of anthropometric parameters using IR molecular fingerprints, related to Figure 2. (**A**) Test ROC curves for distinguishing males from females. Classification performance was estimated by repeatedly training on 90% of the first sample set and testing on the second sample set in 10 iterations, ensuring no overlap between the longitudinally sampled individuals. (**B–C**) Prediction error of regression models trained to estimate (**B**) age and (**C**) body mass index (BMI) of the sampled individuals, combining samples from both sets. The predicted values follow from the test sets of models fit on training sets in a 10-fold cross-validation where each point represents a measurement. (**D–F**) Same procedure, but applied on the healthy subset of the population to reduce the contribution of the health phenotypes on the anthropometric parameter predictions.

		Sample set #1		Sample set #2	
		**Controls**	**Cases**	**Controls**	**Cases**
**Dyslipidemia**	***n***	1298	1298	856	856
	**Male proportion**, %	50.8	50.4	51.6	51.8
	**Age, mean (± SD),** ***years***	56.3 (±13.3)	57.8 (±12.1)	60.7 (±11.5)	60.6 (±11.3)
	**Body mass index, mean (± SD),** ***kg/m***^{2}	27.5 (±4.8)	28.2 (±4.5)	28.4 (±4.8)	28.6 (±4.7)
**Hypertension**	***n***	757	757	551	551
	**Male proportion**, %	50.5	50.5	49.7	49.7
	**Age, mean (± SD),** ***years***	59.4 (±11.3)	61.1 (±11.4)	63.1 (±10.6)	64.6 (±10.4)
	**Body mass index, mean (± SD),** ***kg/m***^{2}	28.0 (±4.1)	28.5 (±4.2)	27.9 (±4.3)	28.5 (±4.4)
**Prediabetes**	***n***	492	492	375	375
	**Male proportion**, %	52.8	52.8	57.1	57.1
	**Age, mean (± SD),** ***years***	63.1 (±11.0)	63.2 (±11.0)	65.1 (±11.1)	65.2 (±11.0)
	**Body mass index, mean (± SD),** ***kg/m***^{2}	29.7 (±4.7)	29.7 (±4.8)	29.6 (±4.8)	29.6 (±4.8)
**Type-2 diabetes**	***n***	335	335	295	295
	**Male proportion**, %	59.4	59.4	60.0	60.0
	**Age, mean (± SD),** ***years***	66.9 (±9.3)	67.1 (±9.3)	69.2 (±9.9)	69.4 (±10.1)
	**Body mass index, mean (± SD),** ***kg/m***^{2}	31.1 (±5.0)	31.2 (±5.1)	30.9 (±5.2)	31.1 (±5.3)
**Healthy**	***n***	715	715	526	526
	**Male proportion**, %	45.5	45.5	43.9	43.9
	**Age, mean (± SD),** ***years***	51.0 (±11.2)	49.3 (±11.4)	56.1 (±10.5)	54.5 (±10.8)
	**Body mass index, mean (± SD),** ***kg/m***^{2}	26.2 (±3.9)	25.8 (±4.0)	26.5 (±3.9)	26.0 (±4.1)

Supplementary Table S2. Description of cohorts used in case-control matched classifications, related to Figure 2 and Figure S6. Matching was performed based on gender, age, and body mass index of the sampled individuals in a pair-wise fashion.

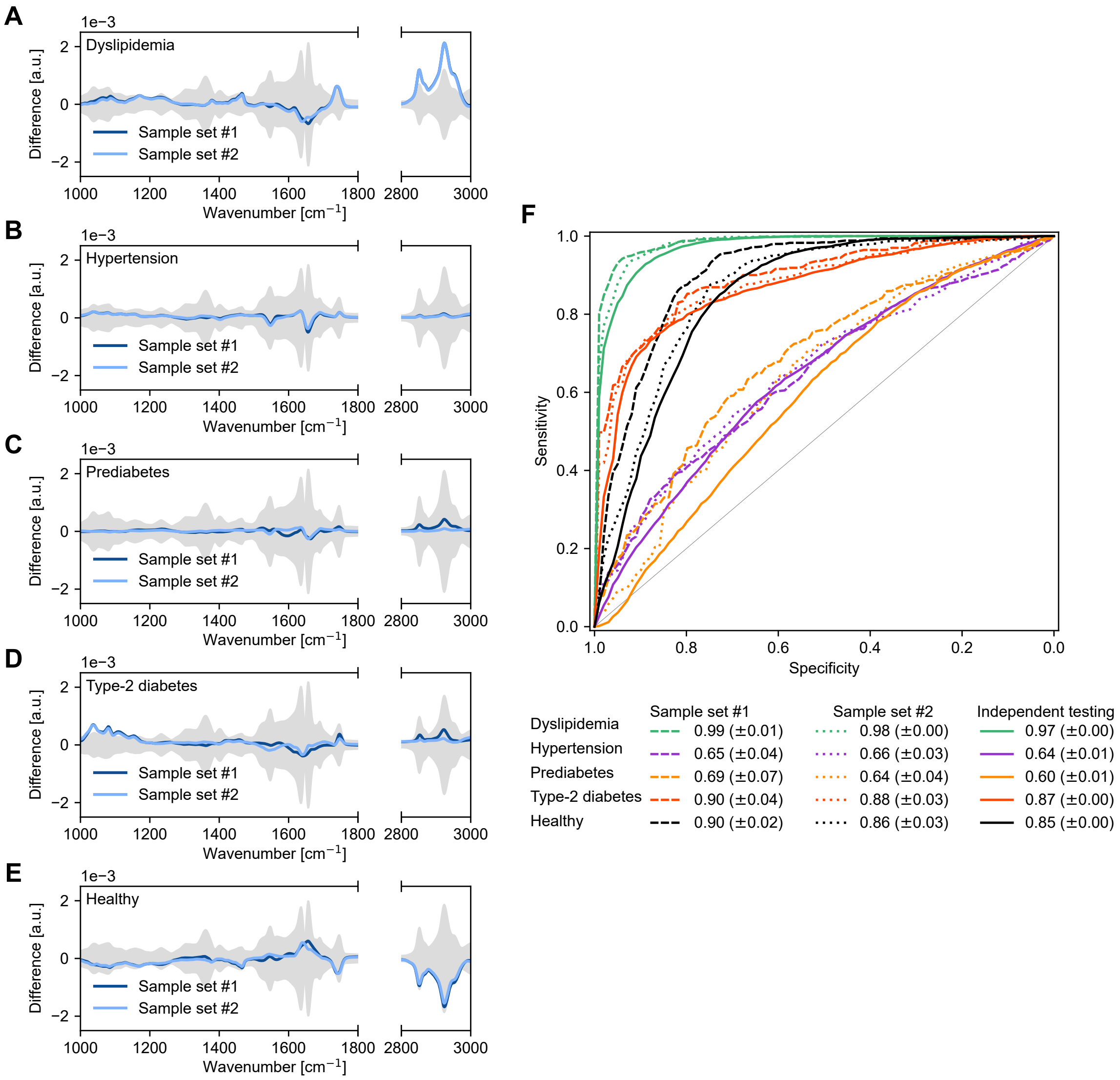

Supplementary Figure S6. Phenotype detection in gender, age, and body mass index matched cohorts using IR molecular fingerprints, related to Figure 2 and Table S2. (**A**–**E**) Difference between the mean spectral measurement of cases and controls for each condition in the first sample set (dark blue) and second sample set (light blue). The "healthy" category includes individuals having none of the other listed conditions. (**F**) ROC curves for the binary classifications detecting each phenotype. The mean cross-validated ROC curve of the test sets is depicted and the area under the curve is listed, along with its standard deviation, for each binary classification.

Sample set #1 (baseline)						
**No. of risk factors**	**Sample count**	**Visceral obesity**	**Hypertriglyceridemia**	**Low HDL cholesterol**	**Hypertension**	**Hyperglycemia**
**0**	547	0 (0.0%)	0 (0.0%)	0 (0.0%)	0 (0.0%)	0 (0.0%)
**1**	709	473 (66.7%)	30 (4.2%)	40 (5.6%)	105 (14.8%)	61 (8.6%)
**2**	659	555 (84.2%)	146 (22.2%)	169 (25.6%)	264 (40.1%)	184 (27.9%)
**3**	548	504 (92.0%)	318 (58.0%)	238 (43.4%)	296 (54.0%)	288 (52.6%)
**4**	365	347 (95.1%)	333 (91.2%)	278 (76.2%)	214 (58.6%)	288 (78.9%)
**5**	163	163 (100.0%)	163 (100.0%)	163 (100.0%)	163 (100.0%)	163 (100.0%)
**Sample set #2 (follow-up)**						
**No. of risk factors**	**Sample count**	**Visceral obesity**	**Hypertriglyceridemia**	**Low HDL cholesterol**	**Hypertension**	**Hyperglycemia**
**0**	291	0 (0.0%)	0 (0.0%)	0 (0.0%)	0 (0.0%)	0 (0.0%)
**1**	562	434 (77.2%)	14 (2.5%)	7 (1.2%)	39 (6.9%)	68 (12.1%)
**2**	488	432 (88.5%)	107 (21.9%)	50 (10.2%)	130 (26.6%)	257 (52.7%)
**3**	375	361 (96.3%)	215 (57.3%)	135 (36.0%)	172 (45.9%)	242 (64.5%)
**4**	290	278 (95.9%)	281 (96.9%)	216 (74.5%)	121 (41.7%)	264 (91.0%)
**5**	97	97 (100.0%)	97 (100.0%)	97 (100.0%)	97 (100.0%)	97 (100.0%)

Supplementary Table S3. Breakdown of metabolic risk factors for pre-MetS and MetS investigations, related to Figure 3. Each row represents a group of individuals with varying numbers of concurrent risk factors. The sample counts and percentages are listed according to the distribution of risk factors observed in each group. Distributions of the first and second sample sets are listed separately. Samples with unknown values for any of the risk factors were excluded.

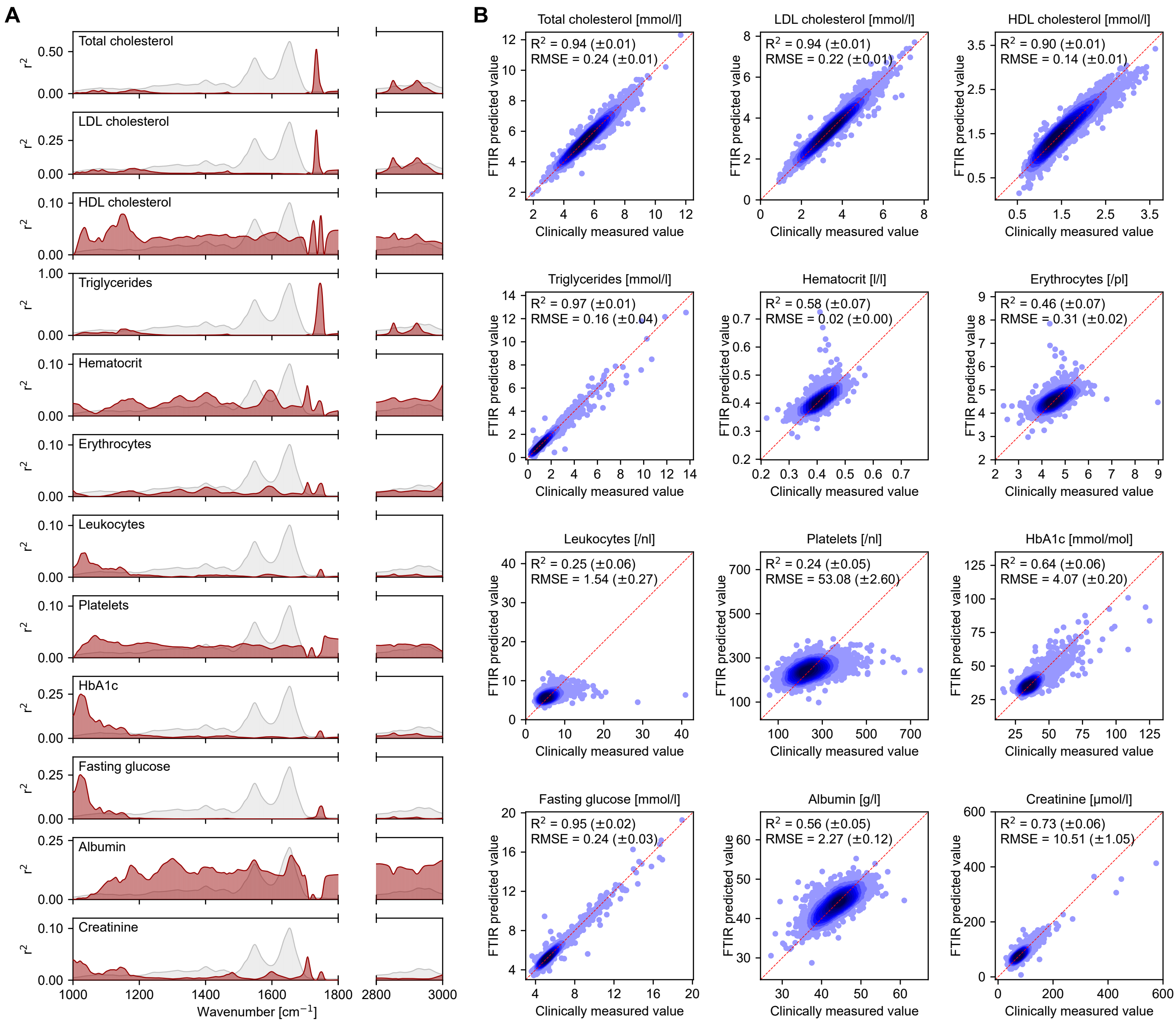

Supplementary Figure S7. Classical clinical analytes reflected in non-normalized IR molecular fingerprints, related to Figure 4. (**A**) Pearson correlation coefficient (red curves) between the concentrations of each clinical analyte and the absorbance at each wavenumber between 1000 and 3000 cm^{-1} for non-normalized spectra. The mean absorbance spectrum of all measured IR spectra (n = 5184) is overlaid in gray on each panel as a visual reference for the shape of the spectrum. (**B**) Performance of quantitatively predicting clinical analytes using the non-normalized IR spectra of the measured population. Regression algorithms were trained for each parameter to capture the relations between the clinically-measured values and the multivariate spectral features. The predicted values follow from the test sets of 10-fold cross-validations. Each point represents a measurement. The mean coefficient of determination (R^2) and the root mean squared error (RMSE) are listed for each parameter, along with their standard deviations across the test splits. The diagonal (dashed red line) is a visual reference for a perfect fit.

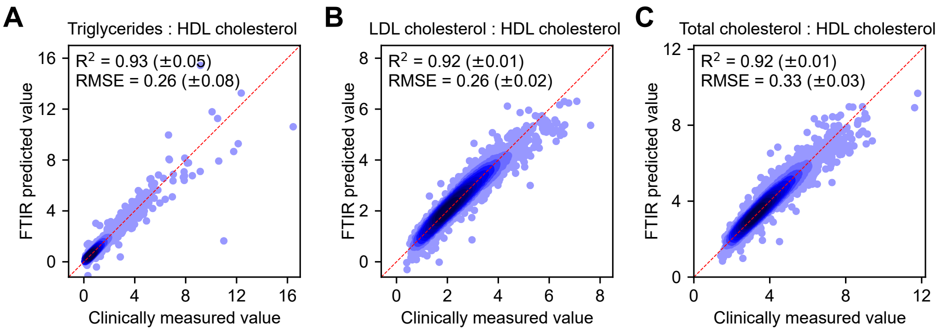

Supplementary Figure S8. Performance of quantitatively predicting cholesterol ratios using IR molecular fingerprints, related to Figure 4. Ratios were calculated by dividing the clinically measured mmol/l concentrations of triglycerides by HDL cholesterol (**A**), LDL cholesterol by HDL cholesterol (**B**), and total cholesterol by HDL cholesterol (**C**). Regression algorithms were trained for each ratio to capture the relations between the calculated values and the multivariate spectral features. Predicted values follow from test sets of models fit on training sets in a 10-fold cross-validation. Each point represents a measurement. Model performance was measured by the mean coefficient of determination (R^2) and the root mean squared error (RMSE) and is listed for each parameter, along the standard deviation, across the test sets of the cross-validation. The diagonal (dashed red line) represents a visual reference for a perfect fit.

Supplementary Table S4. Predictive performance of multilabel classification using different label arrangements for optimal model selection, related to STAR Methods. To select an optimal classification order, several permutations were tested to observe their influence on the overall predictive efficacy. For each listed order, model performance was estimated by cross-validating on only sample set #1 to avoid introducing model selection bias by leaking information from sample set #2 (which was used for the independent testing). The mean and standard deviation of the calculated exact match ratio, Hamming score, and Hamming loss is listed for each of the possible 120 ordering of the 5 binary classifiers in the chain. The last entry in the table lists the performance of a non-chained model where each of the 5 phenotypes was detected with an independent binary classifier. Abbreviations: T2D – type-2 diabetes; PD – prediabetes.

#	Label order	Exact match ratio	Hamming score	1 - Hamming loss
**1**	Dyslipidemia → Hypertension → T2D → PD → Healthy	0.58 (±0.03)	0.71 (±0.03)	0.86 (±0.01)
**2**	Hypertension → T2D → Dyslipidemia → PD → Healthy	0.58 (±0.03)	0.71 (±0.03)	0.86 (±0.01)
**3**	Hypertension → Dyslipidemia → T2D → PD → Healthy	0.57 (±0.03)	0.71 (±0.03)	0.86 (±0.01)
**4**	PD → Hypertension → T2D → Dyslipidemia → Healthy	0.57 (±0.03)	0.70 (±0.03)	0.86 (±0.01)
**5**	Dyslipidemia → Hypertension → PD → T2D → Healthy	0.57 (±0.03)	0.70 (±0.03)	0.86 (±0.01)
**6**	T2D → Hypertension → Dyslipidemia → PD → Healthy	0.57 (±0.03)	0.71 (±0.03)	0.86 (±0.01)
**7**	PD → Hypertension → Dyslipidemia → T2D → Healthy	0.57 (±0.04)	0.70 (±0.03)	0.86 (±0.01)
**8**	Dyslipidemia → PD → Hypertension → T2D → Healthy	0.57 (±0.04)	0.70 (±0.03)	0.86 (±0.01)
**9**	Hypertension → PD → T2D → Dyslipidemia → Healthy	0.57 (±0.03)	0.70 (±0.03)	0.86 (±0.01)
**10**	Hypertension → PD → Dyslipidemia → T2D → Healthy	0.57 (±0.03)	0.70 (±0.03)	0.86 (±0.01)
**11**	Hypertension → Dyslipidemia → PD → T2D → Healthy	0.57 (±0.03)	0.70 (±0.03)	0.86 (±0.01)
**12**	PD → Dyslipidemia → Hypertension → T2D → Healthy	0.57 (±0.04)	0.70 (±0.03)	0.86 (±0.01)
**13**	Dyslipidemia → Hypertension → Healthy → PD → T2D	0.57 (±0.03)	0.70 (±0.03)	0.86 (±0.01)
**14**	PD → T2D → Hypertension → Dyslipidemia → Healthy	0.57 (±0.04)	0.70 (±0.03)	0.86 (±0.01)
**15**	Dyslipidemia → T2D → Hypertension → PD → Healthy	0.57 (±0.03)	0.70 (±0.03)	0.86 (±0.01)
**16**	T2D → Dyslipidemia → Hypertension → PD → Healthy	0.57 (±0.03)	0.70 (±0.03)	0.86 (±0.01)
**17**	Hypertension → T2D → PD → Dyslipidemia → Healthy	0.57 (±0.03)	0.70 (±0.03)	0.86 (±0.01)
**18**	Dyslipidemia → Hypertension → PD → Healthy → T2D	0.57 (±0.03)	0.70 (±0.03)	0.86 (±0.01)
**19**	Hypertension → Dyslipidemia → Healthy → PD → T2D	0.57 (±0.03)	0.70 (±0.03)	0.86 (±0.01)
**20**	PD → Hypertension → Dyslipidemia → Healthy → T2D	0.57 (±0.03)	0.70 (±0.03)	0.86 (±0.01)
**21**	T2D → Hypertension → PD → Dyslipidemia → Healthy	0.57 (±0.03)	0.70 (±0.03)	0.86 (±0.01)
**22**	Dyslipidemia → PD → Hypertension → Healthy → T2D	0.57 (±0.04)	0.70 (±0.03)	0.86 (±0.01)
**23**	Dyslipidemia → PD → T2D → Hypertension → Healthy	0.57 (±0.04)	0.70 (±0.03)	0.86 (±0.01)
**24**	PD → T2D → Dyslipidemia → Hypertension → Healthy	0.57 (±0.04)	0.70 (±0.03)	0.86 (±0.01)
**25**	Hypertension → PD → Dyslipidemia → Healthy → T2D	0.57 (±0.03)	0.70 (±0.03)	0.86 (±0.01)
**26**	Hypertension → Dyslipidemia → PD → Healthy → T2D	0.57 (±0.03)	0.70 (±0.03)	0.86 (±0.01)
**27**	PD → Dyslipidemia → Hypertension → Healthy → T2D	0.57 (±0.04)	0.70 (±0.03)	0.86 (±0.01)
**28**	PD → Dyslipidemia → T2D → Hypertension → Healthy	0.57 (±0.04)	0.70 (±0.03)	0.86 (±0.01)
**29**	Dyslipidemia → Hypertension → T2D → Healthy → PD	0.57 (±0.03)	0.70 (±0.03)	0.86 (±0.01)
**30**	Dyslipidemia → Hypertension → Healthy → T2D → PD	0.57 (±0.03)	0.70 (±0.03)	0.86 (±0.01)
**31**	PD → Hypertension → T2D → Healthy → Dyslipidemia	0.57 (±0.03)	0.70 (±0.03)	0.86 (±0.01)
**32**	T2D → PD → Hypertension → Dyslipidemia → Healthy	0.57 (±0.04)	0.70 (±0.03)	0.86 (±0.01)
**33**	Hypertension → T2D → Dyslipidemia → Healthy → PD	0.57 (±0.03)	0.70 (±0.03)	0.86 (±0.01)
**34**	Hypertension → PD → T2D → Healthy → Dyslipidemia	0.57 (±0.03)	0.70 (±0.03)	0.86 (±0.01)
**35**	Hypertension → Dyslipidemia → T2D → Healthy → PD	0.57 (±0.03)	0.70 (±0.03)	0.86 (±0.01)
**36**	PD → T2D → Hypertension → Healthy → Dyslipidemia	0.57 (±0.04)	0.70 (±0.03)	0.86 (±0.01)
**37**	Dyslipidemia → T2D → PD → Hypertension → Healthy	0.57 (±0.04)	0.70 (±0.03)	0.86 (±0.01)
**38**	T2D → Dyslipidemia → PD → Hypertension → Healthy	0.57 (±0.04)	0.70 (±0.03)	0.86 (±0.01)
**39**	Hypertension → Dyslipidemia → Healthy → T2D → PD	0.57 (±0.03)	0.70 (±0.03)	0.86 (±0.01)
**40**	T2D → Hypertension → Dyslipidemia → Healthy → PD	0.57 (±0.03)	0.70 (±0.03)	0.86 (±0.01)
**41**	Hypertension → T2D → PD → Healthy → Dyslipidemia	0.57 (±0.03)	0.70 (±0.03)	0.86 (±0.01)
**42**	T2D → PD → Dyslipidemia → Hypertension → Healthy	0.57 (±0.04)	0.70 (±0.03)	0.86 (±0.01)
**43**	T2D → Hypertension → PD → Healthy → Dyslipidemia	0.57 (±0.03)	0.70 (±0.03)	0.86 (±0.01)
**44**	Dyslipidemia → T2D → Hypertension → Healthy → PD	0.57 (±0.04)	0.70 (±0.03)	0.86 (±0.01)
**45**	T2D → Dyslipidemia → Hypertension → Healthy → PD	0.57 (±0.03)	0.70 (±0.03)	0.86 (±0.01)
**46**	T2D → PD → Hypertension → Healthy → Dyslipidemia	0.57 (±0.04)	0.70 (±0.03)	0.86 (±0.01)
**47**	PD → Hypertension → Healthy → Dyslipidemia → T2D	0.56 (±0.04)	0.69 (±0.03)	0.85 (±0.01)
**48**	Dyslipidemia → PD → T2D → Healthy → Hypertension	0.56 (±0.04)	0.70 (±0.03)	0.86 (±0.01)
**49**	PD → Hypertension → Healthy → T2D → Dyslipidemia	0.56 (±0.03)	0.69 (±0.03)	0.85 (±0.01)
**50**	PD → T2D → Dyslipidemia → Healthy → Hypertension	0.56 (±0.04)	0.70 (±0.03)	0.86 (±0.01)
**51**	Hypertension → PD → Healthy → Dyslipidemia → T2D	0.56 (±0.03)	0.69 (±0.03)	0.85 (±0.01)
**52**	PD → Dyslipidemia → T2D → Healthy → Hypertension	0.56 (±0.04)	0.70 (±0.03)	0.86 (±0.01)
**53**	Hypertension → PD → Healthy → T2D → Dyslipidemia	0.56 (±0.03)	0.69 (±0.03)	0.85 (±0.01)
**54**	Dyslipidemia → PD → Healthy → Hypertension → T2D	0.56 (±0.03)	0.70 (±0.02)	0.86 (±0.01)
**55**	PD → Dyslipidemia → Healthy → Hypertension → T2D	0.56 (±0.03)	0.70 (±0.02)	0.86 (±0.01)
**56**	Dyslipidemia → PD → Healthy → T2D → Hypertension	0.56 (±0.03)	0.70 (±0.02)	0.86 (±0.01)
**57**	PD → Dyslipidemia → Healthy → T2D → Hypertension	0.56 (±0.03)	0.70 (±0.02)	0.86 (±0.01)
**58**	Dyslipidemia → T2D → PD → Healthy → Hypertension	0.56 (±0.04)	0.70 (±0.03)	0.86 (±0.01)
**59**	T2D → Dyslipidemia → PD → Healthy → Hypertension	0.56 (±0.04)	0.70 (±0.03)	0.86 (±0.01)
**60**	T2D → PD → Dyslipidemia → Healthy → Hypertension	0.56 (±0.04)	0.70 (±0.03)	0.86 (±0.01)

Continued on next page.

Supplementary Table S4. *Continued from previous page.*

#	Label order	Exact match ratio	Hamming score	1 - Hamming loss
**61**	Hypertension → T2D → Healthy → Dyslipidemia → PD	0.56 (±0.03)	0.69 (±0.03)	0.86 (±0.01)
**62**	Hypertension → Healthy → PD → Dyslipidemia → T2D	0.56 (±0.03)	0.69 (±0.03)	0.85 (±0.01)
**63**	Hypertension → T2D → Healthy → PD → Dyslipidemia	0.56 (±0.03)	0.69 (±0.03)	0.86 (±0.01)
**64**	T2D → Hypertension → Healthy → Dyslipidemia → PD	0.56 (±0.03)	0.69 (±0.03)	0.86 (±0.01)
**65**	Hypertension → Healthy → PD → T2D → Dyslipidemia	0.56 (±0.03)	0.69 (±0.03)	0.85 (±0.01)
**66**	Dyslipidemia → Healthy → Hypertension → PD → T2D	0.56 (±0.03)	0.69 (±0.02)	0.85 (±0.01)
**67**	T2D → Hypertension → Healthy → PD → Dyslipidemia	0.56 (±0.03)	0.69 (±0.03)	0.86 (±0.01)
**68**	Hypertension → Healthy → Dyslipidemia → PD → T2D	0.56 (±0.03)	0.69 (±0.03)	0.85 (±0.01)
**69**	Dyslipidemia → Healthy → PD → Hypertension → T2D	0.55 (±0.03)	0.69 (±0.02)	0.85 (±0.01)
**70**	Dyslipidemia → Healthy → PD → T2D → Hypertension	0.55 (±0.03)	0.69 (±0.02)	0.85 (±0.01)
**71**	Hypertension → Healthy → Dyslipidemia → T2D → PD	0.55 (±0.03)	0.69 (±0.03)	0.86 (±0.01)
**72**	Dyslipidemia → Healthy → Hypertension → T2D → PD	0.55 (±0.03)	0.69 (±0.03)	0.86 (±0.01)
**73**	Dyslipidemia → Healthy → T2D → Hypertension → PD	0.55 (±0.03)	0.69 (±0.02)	0.86 (±0.01)
**74**	Hypertension → Healthy → T2D → Dyslipidemia → PD	0.55 (±0.03)	0.68 (±0.03)	0.86 (±0.01)
**75**	Hypertension → Healthy → T2D → PD → Dyslipidemia	0.55 (±0.03)	0.69 (±0.03)	0.86 (±0.01)
**76**	Dyslipidemia → Healthy → T2D → PD → Hypertension	0.55 (±0.03)	0.69 (±0.03)	0.86 (±0.01)
**77**	Dyslipidemia → T2D → Healthy → Hypertension → PD	0.55 (±0.03)	0.69 (±0.03)	0.86 (±0.01)
**78**	T2D → Dyslipidemia → Healthy → Hypertension → PD	0.55 (±0.03)	0.69 (±0.03)	0.86 (±0.01)
**79**	Dyslipidemia → T2D → Healthy → PD → Hypertension	0.55 (±0.04)	0.69 (±0.03)	0.86 (±0.01)
**80**	T2D → Dyslipidemia → Healthy → PD → Hypertension	0.55 (±0.03)	0.69 (±0.03)	0.86 (±0.01)
**81**	PD → T2D → Healthy → Hypertension → Dyslipidemia	0.54 (±0.03)	0.67 (±0.02)	0.85 (±0.01)
**82**	PD → T2D → Healthy → Dyslipidemia → Hypertension	0.54 (±0.03)	0.67 (±0.02)	0.85 (±0.01)
**83**	T2D → PD → Healthy → Hypertension → Dyslipidemia	0.53 (±0.03)	0.67 (±0.02)	0.85 (±0.01)
**84**	T2D → PD → Healthy → Dyslipidemia → Hypertension	0.53 (±0.03)	0.67 (±0.02)	0.85 (±0.01)
**85**	PD → Healthy → Dyslipidemia → Hypertension → T2D	0.53 (±0.03)	0.67 (±0.02)	0.84 (±0.01)
**86**	PD → Healthy → Dyslipidemia → T2D → Hypertension	0.53 (±0.03)	0.67 (±0.02)	0.84 (±0.01)
**87**	PD → Healthy → Hypertension → Dyslipidemia → T2D	0.53 (±0.03)	0.67 (±0.02)	0.84 (±0.01)
**88**	PD → Healthy → T2D → Dyslipidemia → Hypertension	0.53 (±0.03)	0.67 (±0.02)	0.84 (±0.01)
**89**	PD → Healthy → T2D → Hypertension → Dyslipidemia	0.53 (±0.02)	0.66 (±0.02)	0.84 (±0.01)
**90**	PD → Healthy → Hypertension → T2D → Dyslipidemia	0.53 (±0.03)	0.66 (±0.02)	0.84 (±0.01)
**91**	Healthy → Dyslipidemia → Hypertension → PD → T2D	0.53 (±0.03)	0.66 (±0.02)	0.84 (±0.01)
**92**	Healthy → PD → Dyslipidemia → Hypertension → T2D	0.52 (±0.02)	0.66 (±0.02)	0.84 (±0.01)
**93**	Healthy → Hypertension → PD → Dyslipidemia → T2D	0.52 (±0.03)	0.66 (±0.02)	0.84 (±0.01)
**94**	Healthy → PD → Dyslipidemia → T2D → Hypertension	0.52 (±0.02)	0.66 (±0.02)	0.84 (±0.01)
**95**	Healthy → Dyslipidemia → PD → T2D → Hypertension	0.52 (±0.03)	0.66 (±0.02)	0.84 (±0.01)
**96**	Healthy → Dyslipidemia → PD → Hypertension → T2D	0.52 (±0.02)	0.66 (±0.02)	0.84 (±0.01)
**97**	Healthy → PD → Hypertension → Dyslipidemia → T2D	0.52 (±0.02)	0.66 (±0.02)	0.84 (±0.01)
**98**	Healthy → Hypertension → PD → T2D → Dyslipidemia	0.52 (±0.02)	0.66 (±0.02)	0.84 (±0.01)
**99**	Healthy → PD → T2D → Dyslipidemia → Hypertension	0.52 (±0.02)	0.66 (±0.02)	0.84 (±0.01)
**100**	Healthy → Hypertension → Dyslipidemia → PD → T2D	0.52 (±0.02)	0.66 (±0.02)	0.84 (±0.01)
**101**	Healthy → PD → T2D → Hypertension → Dyslipidemia	0.52 (±0.02)	0.66 (±0.02)	0.84 (±0.01)
**102**	T2D → Healthy → Dyslipidemia → Hypertension → PD	0.52 (±0.03)	0.66 (±0.02)	0.85 (±0.01)
**103**	Healthy → PD → Hypertension → T2D → Dyslipidemia	0.52 (±0.02)	0.66 (±0.02)	0.84 (±0.01)
**104**	Healthy → Dyslipidemia → Hypertension → T2D → PD	0.52 (±0.03)	0.66 (±0.02)	0.85 (±0.01)
**105**	Healthy → Dyslipidemia → T2D → Hypertension → PD	0.52 (±0.03)	0.66 (±0.02)	0.85 (±0.01)
**106**	T2D → Healthy → PD → Dyslipidemia → Hypertension	0.52 (±0.03)	0.66 (±0.02)	0.85 (±0.01)
**107**	T2D → Healthy → PD → Hypertension → Dyslipidemia	0.52 (±0.03)	0.66 (±0.03)	0.85 (±0.01)
**108**	T2D → Healthy → Dyslipidemia → PD → Hypertension	0.52 (±0.03)	0.66 (±0.02)	0.85 (±0.01)
**109**	T2D → Healthy → Hypertension → Dyslipidemia → PD	0.52 (±0.03)	0.66 (±0.03)	0.85 (±0.01)
**110**	Healthy → T2D → Dyslipidemia → Hypertension → PD	0.52 (±0.02)	0.66 (±0.02)	0.85 (±0.01)
**111**	Healthy → Dyslipidemia → T2D → PD → Hypertension	0.52 (±0.03)	0.66 (±0.02)	0.85 (±0.01)
**112**	Healthy → Hypertension → Dyslipidemia → T2D → PD	0.52 (±0.02)	0.66 (±0.02)	0.85 (±0.01)
**113**	T2D → Healthy → Hypertension → PD → Dyslipidemia	0.52 (±0.03)	0.66 (±0.03)	0.85 (±0.01)
**114**	Healthy → T2D → PD → Dyslipidemia → Hypertension	0.52 (±0.03)	0.66 (±0.02)	0.85 (±0.01)
**115**	Healthy → T2D → Dyslipidemia → PD → Hypertension	0.52 (±0.03)	0.66 (±0.02)	0.85 (±0.01)
**116**	Healthy → Hypertension → T2D → Dyslipidemia → PD	0.52 (±0.02)	0.66 (±0.02)	0.85 (±0.01)
**117**	Healthy → Hypertension → T2D → PD → Dyslipidemia	0.52 (±0.03)	0.66 (±0.02)	0.85 (±0.01)
**118**	Healthy → T2D → PD → Hypertension → Dyslipidemia	0.52 (±0.02)	0.66 (±0.02)	0.85 (±0.01)
**119**	Healthy → T2D → Hypertension → Dyslipidemia → PD	0.52 (±0.03)	0.66 (±0.02)	0.85 (±0.01)
**120**	Healthy → T2D → Hypertension → PD → Dyslipidemia	0.52 (±0.03)	0.66 (±0.02)	0.85 (±0.01)
**121**	Non-chained model (label order not applicable)	0.50 (±0.02)	0.64 (±0.02)	0.86 (±0.01)

Input	True label ($\mathbf{y}_i$)	Model output ($\hat{\mathbf{y}}_i$)	Exact match ratio	Hamming score	1 - Hamming loss
$\mathbf{x}_1$	$[1\,0\,0\,0\,0]$	$[0\,1\,0\,0\,0]$	0.00	$0/2 = 0.00$	$3/5 = 0.60$
$\mathbf{x}_2$	$[1\,0\,0\,0\,0]$	$[1\,1\,0\,0\,0]$	0.00	$1/2 = 0.50$	$4/5 = 0.80$
$\mathbf{x}_3$	$[1\,1\,0\,0\,0]$	$[1\,0\,1\,0\,0]$	0.00	$1/3 = 0.33$	$3/5 = 0.60$
$\mathbf{x}_4$	$[1\,1\,0\,0\,0]$	$[1\,1\,1\,0\,0]$	0.00	$2/3 = 0.67$	$4/5 = 0.80$
$\mathbf{x}_5$	$[1\,1\,1\,0\,0]$	$[1\,1\,1\,1\,0]$	0.00	$3/4 = 0.75$	$4/5 = 0.80$
$\mathbf{x}_6$	$[1\,1\,1\,1\,0]$	$[1\,1\,1\,1\,0]$	1.00	$4/4 = 1.00$	$5/5 = 1.00$
**Average**			**0.17**	**0.54**	**0.77**

Supplementary Table S5. Example of evaluating multilabel classification metrics, related to STAR Methods. The exact match ratio, Hamming score, and Hamming loss metrics were computed and provided as examples to demonstrate the accuracy assessment between predictions and true labels. $\mathbf{x}_i$ represents an input sample, $\mathbf{y}_i$ is a five-dimensional vector of the true labels for the sample, and $\hat{\mathbf{y}}_i$ is the vector of predicted labels. Erroneous label predictions are highlighted in red.
