# Supplementary material for: Plasma infrared fingerprinting with machine learning enables single-measurement multi-phenotype health screening
Source: Cell Rep Med. 2024 Jun 28;5(7):101625. doi: 10.1016/j.xcrm.2024.101625 (PMC11293328; doi:10.1016/j.xcrm.2024.101625)
Supplement: Document S2. Article plus supplemental information [file mmc2.pdf]

Cell Reports
Medicine

Article

Plasma infrared fingerprinting with machine learning enables single-measurement multi-phenotype health screening

Graphical abstract

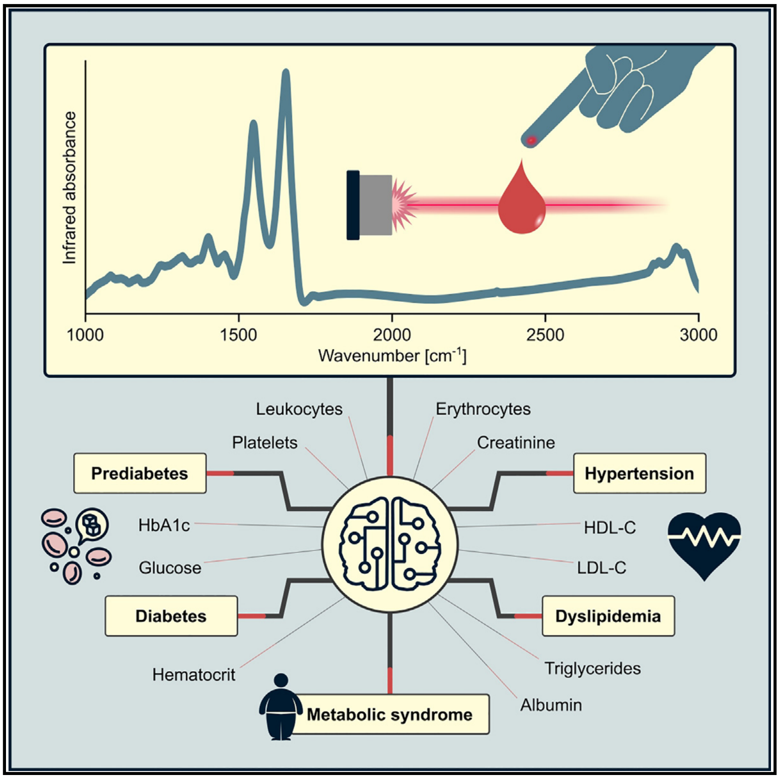

Highlights

- Multimorbidities are detectable through infrared molecular fingerprinting of plasma
- Infrared fingerprints forecast metabolic syndrome within 6.5 years pre-onset
- Concentrations of clinical chemistry analytes are reflected in infrared fingerprints
- Infrared fingerprinting enables cost-effective, high-throughput clinical diagnostics

Authors

Tarek Eissa, Cristina Leonardo, Kosmas V. Kepesidis, ..., Lothar Richter, Annette Peters, Mihaela Žigman

Correspondence

tarek.eissa@mpq.mpg.de (T.E.),
mihaela.zigman@mpq.mpg.de (M.Ž.)

In brief

Eissa et al. put forward a cost-effective diagnostic platform for high-throughput health profiling via infrared spectroscopy of blood plasma. Using a population-based study, they develop machine learning models to detect chronic health conditions and multimorbidities, forecast the development of metabolic syndrome, and quantify the concentrations of classical clinical chemistry analytes.

Eissa et al., 2024, Cell Reports Medicine *5*, 101625
July 16, 2024 © 2024 The Authors. Published by Elsevier Inc.
https://doi.org/10.1016/j.xcrm.2024.101625

CellPress

Cell Reports Medicine

CellPress
OPEN ACCESS

Article

Plasma infrared fingerprinting with machine learning enables single-measurement multi-phenotype health screening

Tarek Eissa,[1,2,3,*] Cristina Leonardo,[1] Kosmas V. Kepesidis,[1,2,4] Frank Fleischmann,[1,2] Birgit Linkohr,[5] Daniel Meyer,[2,4] Viola Zoka,[1,4] Marinus Huber,[1,2] Liudmila Voronina,[1] Lothar Richter,[3] Annette Peters,[5,6,7,8] and Mihaela Žigman[1,2,9,*]

[1]Department of Laser Physics, Ludwig Maximilian University of Munich (LMU), Garching, Germany
[2]Laboratory for Attosecond Physics, Max Planck Institute of Quantum Optics (MPQ), Garching, Germany
[3]School of Computation, Information and Technology, Technical University of Munich (TUM), Garching, Germany
[4]Center for Molecular Fingerprinting (CMF), Budapest, Hungary
[5]Institute of Epidemiology, Helmholtz Zentrum München, Neuherberg, Germany
[6]School of Public Health, Institute for Medical Information Processing, Biometry, and Epidemiology, Pettenkofer, Ludwig Maximilian University of Munich (LMU), Munich, Germany
[7]German Center for Diabetes Research (DZD), Neuherberg, Germany
[8]German Centre for Cardiovascular Research (DZHK), Partner Site Munich, Munich, Germany
[9]Lead contact
*Correspondence: tarek.eissa@mpq.mpg.de (T.E.), mihaela.zigman@mpq.mpg.de (M.Ž.)
https://doi.org/10.1016/j.xcrm.2024.101625

SUMMARY

Infrared spectroscopy is a powerful technique for probing the molecular profiles of complex biofluids, offering a promising avenue for high-throughput *in vitro* diagnostics. While several studies showcased its potential in detecting health conditions, a large-scale analysis of a naturally heterogeneous potential patient population has not been attempted. Using a population-based cohort, here we analyze 5,184 blood plasma samples from 3,169 individuals using Fourier transform infrared (FTIR) spectroscopy. Applying a multi-task classification to distinguish between dyslipidemia, hypertension, prediabetes, type 2 diabetes, and healthy states, we find that the approach can accurately single out healthy individuals and characterize chronic multimorbid states. We further identify the capacity to forecast the development of metabolic syndrome years in advance of onset. Dataset-independent testing confirms the robustness of infrared signatures against variations in sample handling, storage time, and measurement regimes. This study provides the framework that establishes infrared molecular fingerprinting as an efficient modality for populational health diagnostics.

INTRODUCTION

The pursuit of molecular gateways that describe the composition of chemically complex media in a minimally invasive, cost-effective, and high-throughput manner is a long-standing challenge.[1,2] As physiological phenotypes often manifest in the molecular profiles of systemic biofluids (e.g., blood), gateways to quantitatively profile their compositions are invaluable for detecting and characterizing changing health states.[3,4]

As minimally invasive screening and risk assessment tools, blood-based laboratory test panels are often employed in clinical diagnostics.[5,6] Despite their foundational role in modern medicine, the process of measuring several biomarkers can be time-consuming and resource-intensive.[7] Furthermore, biomarkers were developed in candidate approaches—requiring decades of research to link any given molecule to a physiological state.

"Shotgun" approaches that simultaneously probe an ensemble of molecules, requiring no *a priori* information, thus carry the potential to forge the future of efficient clinical testing.[8–10] Untargeted mass spectrometry and nuclear magnetic resonance spectroscopy are two highly promising technologies that may fulfill this role. Still, concerns relating to low measurement throughput, run-to-run and between-lab variability, and involved experimental handling have hindered their applications in high-throughput health diagnostics beyond clinical studies.[8–11]

Infrared (IR) spectroscopy is a powerful top-down approach that enables the label-free profiling of a given sample by interrogating molecular fragments (i.e., functional groups) and measuring their resonant vibrational response to IR excitation.[12–14] This technique can employ various light sources, including globars,[15] few-cycle laser-based excitation,[16,17] and discrete wavelength sources such as quantum cascade lasers.[18,19] In broadband IR absorption spectroscopy, absorption occurs when the IR source emits frequencies that align with the vibrational frequency of a molecular fragment. When analyzing complex samples, the presence of similar chemical bonds and functional groups in different biomolecules leads to overlapping spectral response signals that

© 2024 The Authors. Published by Elsevier Inc.
This is an open access article under the CC BY license (http://creativecommons.org/licenses/by/4.0/).

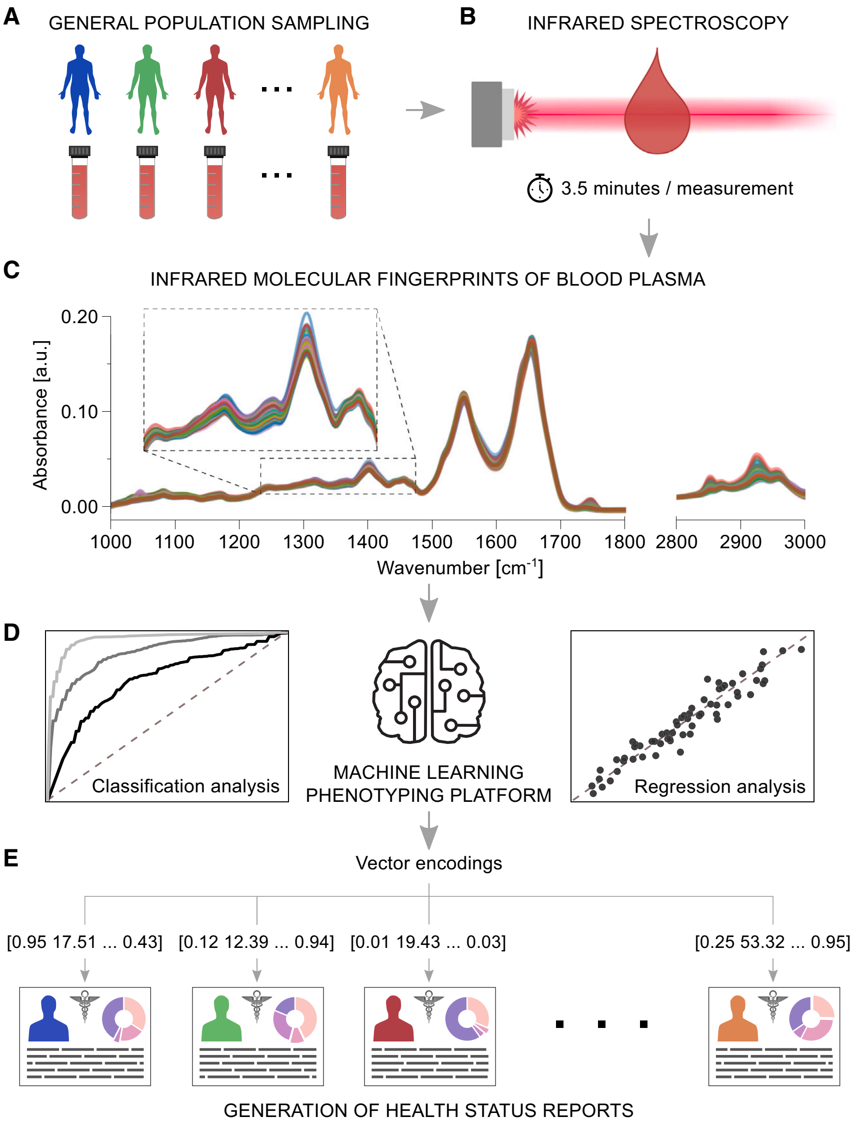

Figure 1. Proposed IR molecular fingerprinting strategy to aid clinical phenotyping and decision-making

(A) Blood samples are drawn, and blood plasma is prepared from individuals with unknown health states, or phenotypes.

(B) Collected plasma samples are measured using an automated FTIR spectrometer.

(C) Preprocessed absorption spectra of liquid blood plasma are captured. The inset depicts a close-up of the spectra in a truncated spectral region.

(D) Machine learning methods are applied to decode the measured spectra into numerical vectors, modeling diverse biological outcomes.

(E) Health reports are generated from a framework established on the measured IR spectra and machine learning models, which quantify unknown health-related properties of each individual to describe their physiology.

hinder the identification of individual constituents. The measured IR absorption spectrum is characteristic of a sample's overall molecular composition, representing a superposition of the responses of all fragments. The corresponding IR absorption spectrum is thus referred to as a molecular fingerprint—simultaneously integrating the entire set of *omes* across biomolecules (e.g., proteins, lipids, and metabolites).[20] When analyzing systemic biofluids, such as plasma, physiological phenotypes may be encoded within the molecular fingerprints as spectral patterns, and machine learning algorithms can be trained to detect them—linking IR fingerprints to multiple phenotypes (Figure 1).

Applications of IR fingerprinting to address medical problems have been previously examined, including numerous studies applying different approaches of IR spectroscopy for *in vitro* clinical diagnostics.[20–35] However, the concept of biofluid IR fingerprinting for medical health diagnostics or screening has not been evaluated in a large-scale naturally variable population, nor has the approach been introduced into the canon of healthcare practices.[36,37] To test its capacity—as an *in vitro* phenotype medical diagnostic tool—large-scale clinical studies involving deeply parametrized individuals whose physiology reflects a diverse patient population are required. Moreover, covariates potentially affecting IR fingerprints (e.g., anthropometric parameters or comorbidities) are yet to be established. Furthermore, trained phenotype detection models must be robustly validated in ways that closely align with real-world scenarios. Hindered by the scale of efforts required, progress toward incorporating IR-based phenotyping in medical diagnostic routines has been limited.[38]

To assess whether an alliance between IR fingerprinting and machine learning could fuel a single-measurement multi-phenotype medical diagnostics platform, we applied Fourier transform IR (FTIR) spectroscopy to profile blood plasma in a large phenotyped cohort. Experimentally, we measured 5,184 samples from 3,169 individuals within the population-based Cooperative Health Research in the Region of Augsburg (KORA) longitudinal cohort,[39] representing a potential patient population. Profiling the largest population with IR fingerprinting to our knowledge, our study aimed to examine the capacity of the approach to comprehensively detect several medically relevant human health phenotypes. With an age range of 32–88 years and the corresponding frequent occurrence of multimorbidity in the older age groups, we focused on the capacity to simultaneously detect a set of commonly occurring phenotypes—dyslipidemia, hypertension, prediabetes, type 2 diabetes, interrelated conditions of the metabolic syndrome (MetS), and healthy states. We employed different methods of validating how such a parallel, multilabel

phenotype classification model performed to examine the utility of the proposed approach. Furthermore, we examined the correlations between clinical laboratory test analytes and the informational content of IR spectra, facilitating their broader interpretability in a known clinical context. Our results provide a strategy to enable a robust and efficient multi-phenotyping platform.

RESULTS

Study characteristics for testing IR fingerprinting in populational screening

To test the performance of IR fingerprinting, we analyzed the KORA population-based longitudinal cohort.[39] The study cohort comprised a random sample from the general adult population, aged 32 to 88 years, in Southern Germany (STAR Methods; Table S1). Our analysis included samples from two time points within the cohort: KORA-F4, sample set #1 (conducted between 2006 and 2008) and KORA-FF4, sample set #2 (conducted between 2013 and 2014), both of which are follow-ups of the KORA-S4 study (conducted between 1999 and 2001). The first sample set (KORA-F4) included 3,044 participants, while the second (KORA-FF4) included 2,140 participants. In total, 5,184 blood plasma samples from 3,169 unique individuals were considered. Among these, 2,015 individuals participated in both sample sets. Extensive medical examinations were performed on the participants using standardized protocols (STAR Methods). FTIR spectroscopy in transmission mode was performed on all 5,184 samples in two independent measurement campaigns—one for each sample set—where the measurement times were separated by an average of 2.7 years. The two sample sets were thus collected at different times, freezer-stored for varying periods, and measured independently—all on a multi-year scale (STAR Methods).

Combining IR spectroscopy of human blood plasma with machine learning analysis, we put forward a rapid approach for describing several characteristics of human health at a populational level (Figure 1). Our study aimed to investigate the potential of IR molecular fingerprints to simultaneously detect commonly occurring chronic conditions and non-communicable diseases (NCDs) through a single, cost-efficient sample measurement.

As proof of concept, we focused on conditions that were highly prevalent in multimorbid individuals (Figure 2A). Dyslipidemia was the most frequently occurring condition among the population, accounting for a total of 49% of the first sample set and 42% of the second sample set. Hypertension was the second most commonly occurring condition, observed in 38% of the individuals in the first sample set and 39% of the individuals in the second. Prediabetic and type 2 diabetic individuals accounted for 28% of the first sample set and 33% of the second when combined. Corresponding to these terms, we defined an additional category of individuals as "healthy"—encompassing individuals negative for each of the four aforementioned phenotypes. Across the 5 phenotypes, we observed that the investigated conditions did not occur randomly in individuals but in certain natural combinations, such that 12 different co-occurrence patterns existed with varying prevalence (Figure 2A). The healthy group was found to be the largest sub-category of individuals in both sample sets.

IR fingerprinting enables simultaneous detection of multiple phenotypes

The capacity to capture and distinguish if a single phenotype or any combination of phenotypes existed in an individual would be essential for health screening and monitoring.[40,41] To evaluate whether IR molecular fingerprinting has any capacity herein, we first examined the study cohort in a cross-sectional fashion. Specifically, we tested whether the approach was sensitive and robust enough to simultaneously detect the presence of dyslipidemia, hypertension, prediabetes, and type 2 diabetes and the absence of these conditions (the healthy group).

We developed a multilabel machine learning classifier capable of simultaneously detecting and distinguishing between the conditions of interest, with the output being a five-dimensional vector describing the phenotypic state of each individual (Figure 2B). The classifier incorporated a procedure in which the task was broken down into five binary classifications linked through a chain of decisions (STAR Methods). This allowed the multilabel classifier to consider correlations between the outcomes, leading to improved detection performance, as well as consider mutual exclusivities (e.g., diabetic individuals cannot be prediabetic and an individual with any chronic condition cannot be in the healthy group).

The inclusion of two sample sets from the examined study population allowed us to test how the classification performed with several validation methods (STAR Methods; Figure S1). First, we applied a 10-fold cross-validation, independently, on each of the two sample sets. This resulted in two classification performance estimates (one for the sample set #1 and another one for the sample set #2), whereby the two sample sets were collected as well as measured years apart. Yet, since a significant portion of individuals were sampled at both time points, and their samples contributed to both sample sets, the cross-validated metrics are not truly independent of each other in the initial examination here. To overcome this and ensure a robust evaluation unbiased by any possible overlap of the involved individuals, we applied a further validation strategy to examine how the classification performed when tested on samples from entirely different individuals. Specifically, the classifier was repeatedly trained on a portion of individuals from sample set #1 and tested on a held-out portion of independent individuals from sample set #2 (STAR Methods). This strategy was designed to test the classification's generalizability over sample storage time, measurement time, as well as across different individuals.

The complex multi-output nature of this classification, which encompasses the occurrence of 12 clinically feasible phenotype combinations (Figure 2A), requires a more thoughtful evaluation as predictions may be partially correct. We therefore considered three established metrics that measure multilabel prediction efficacy in different ways: the exact match ratio, Hamming score, and Hamming loss (metric definitions further explained in STAR Methods).[42–45] Briefly, the exact match ratio rewards predictions that accurately identify the physiological state of a sample across all possible outcomes—scoring each 5-dimensional prediction with either a 0 or 1. The metric is strict as it treats partially correct predictions as wholly incorrect. In contrast, the Hamming score considers partially correct predictions as having value. For a given sample, it is defined as the proportion of

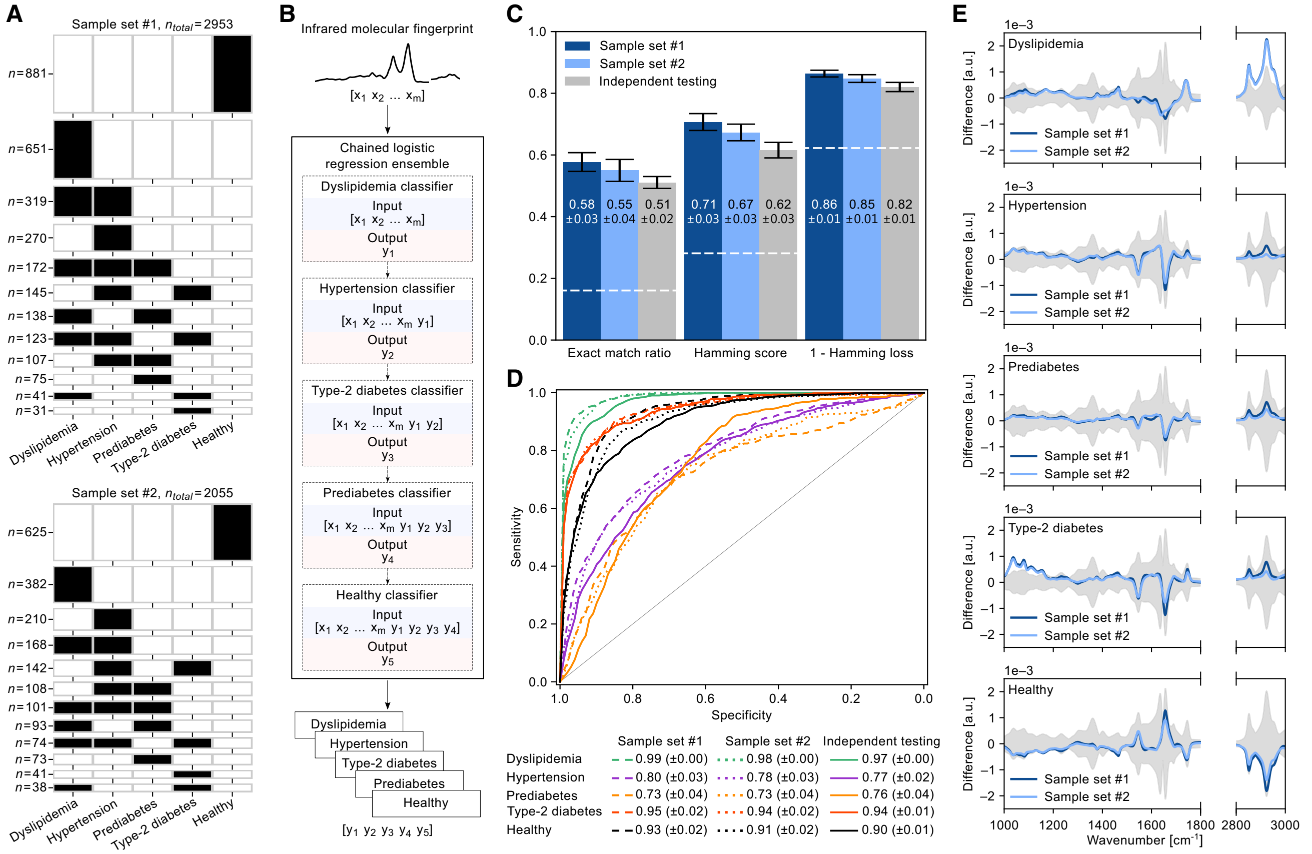

Figure 2. Detection of highly prevalent and co-occurring phenotypes using IR molecular fingerprints

(A) Distributions of all possible combinations of the investigated phenotypes in sample set #1 (upper panel) and sample set #2 (lower panel), encompassing the study cohort. Due to unknown/missing values in the medical records relevant to the listed phenotypes, 91 samples (out of 3,044) were excluded from sample set #1 and 85 samples (out of 2,140) were excluded from sample set #2. Individuals with listed phenotypes are highlighted in black, with each row representing a group of combined phenotypes and its height scaled by the number of individuals in that group.

(B) Model architecture of a multilabel classifier trained to describe the phenotypic state of each input IR spectrum, giving out a five-dimensional vector. A chained binary classification modeling method was applied where each model classifies one outcome against all others using the depicted classification order.

(C) Multilabel classification performance estimates for models trained to simultaneously predict all outcomes of a given IR fingerprint. Classification performance was estimated on unseen test samples by independently cross-validating on each sample set (sample set #1 in dark blue bar and sample set #2 in light blue bar). For independent testing (gray bar), classification performance was estimated by training on a portion of sample set #1 and testing the trained classifier on entirely independent individuals from sample set #2. This provides an estimate of classification generalization across measurements performed years apart, encompassing different training-test individuals. The white dashed lines are a reference that depicts estimates for random chance multilabel predictions for each classification metric (STAR Methods).

(D) Receiver operating characteristic (ROC) curves for binary classifications modeling each listed outcome against the remaining of the population. For each classification, the mean cross-validated ROC curves of the test sets are depicted and the area under the curve is listed below, along with its standard deviation.

(E) Difference between the mean IR fingerprints of samples positive and negative for each phenotype on each sample set (sample set #1 in dark blue and sample set #2 in light blue). Gray-shaded areas depict the standard deviation of IR fingerprints negative for each corresponding phenotype.

correctly predicted positive outcomes to the total number of predicted/actually positive outcomes for the sample. The Hamming loss is defined as the proportion of incorrectly predicted labels across all samples and the 5 labels. All three metrics range from 0 to 1, where higher values for the exact match ratio and Hamming score indicate better predictive performance, while a lower Hamming loss indicates better performance. To provide a consistent interpretation, we calculate 1 − Hamming loss, where higher values also signify better predictive performance. Unlike binary predictions, where a threshold of 0.50 is often assumed to gauge classifiers making random predictions, a contextually relevant point of comparison for multilabel classification is more complex and dependent on the definition of the evaluation metric (details on chance predictions in STAR Methods).

Applying the multilabel classification, we found that the approach was capable of predicting all the exact phenotypic outcomes of the probed individuals with exact match ratios of 0.58 and 0.55 when independently cross-validated on each of the two sample sets (Figure 2C, light/dark blue bars). Importantly, the performance of the classification was stable between sample set #1 and #2, with differences falling within the standard deviation of the validation splits. When the classification was

repeatedly tested on the sample-set-independent measurements, we observed an exact match ratio of 0.51, which significantly exceeds a chance model (Figure 2C, gray bars). The Hamming loss ranged between 0.82 and 0.86 across the three validation methods—revealing that, in the worst case, only 18% of the individual phenotypes were misclassified across all the test samples. In light of the simplicity and cost-effectiveness of the single-measurement IR fingerprinting method, combined with the medical challenge to assess several phenotypes simultaneously, it was surprising to find such high multilabel prediction rates across the three metrics and validation methods. This underscores that the approach is not only sensitive to the diversity of phenotypes but also specific to medical aberrations and their combinations.

This investigation also revealed that the classification performance estimates derived through cross-validation were fairly consistent with how the classifier performed when tested on dataset-independent samples—a validation approach that more closely mirrors practical applications. However, it is important to highlight that a minor drop in predictive efficacy was observed. This drop underscores the importance of validating how such classifications perform when tested on samples that were collected and measured by different operators at considerably different time points from the training dataset. Such dataset-independent validation helps prevent the potential of overestimating how a classification would perform in more realistic applications. Despite this drop, it was surprising to observe that the phenotypes were encoded in IR molecular fingerprints to an extent that enabled their robust detection, even though variations between the sample sets and measurement conditions had significant effects on the spectra (Figure S2).

Phenotype-dependent detection capacity

Since the multilabel classification was built on a series of binary classifiers, where the predictions were driven exclusively by the information encoded in an input IR spectrum, we further examined the diagnostic capacity of each individual phenotype-specific classifier (Figure 2D). This analysis provides a quantitative assessment of the degree to which each individual phenotype was detectable by the IR spectra in a case-control setting—where the control class here combined the set of the remaining four phenotypes (i.e., one-vs-rest analysis). To assess predictive performance, we computed the receiver operating characteristic (ROC) curves of each binary classification, following the same validation procedure as previously described in the multilabel analysis. To combine the measures of sensitivity and specificity, we calculated the area under the ROC curve (AUC) as a summarizing metric of performance.

The AUCs ranged from 0.76 for prediabetes to 0.97 for dyslipidemia when independently tested, which shows that all 5 phenotypes were detectable with a high degree of confidence (Figure 2D). The highest prediction efficacies were observed for type 2 diabetes and dyslipidemia, underscoring the approach's potential for these conditions. Intriguingly, the classifier detecting whether an individual was healthy achieved an AUC of 0.90 when employing the sample set-independent testing method. This revealed that the IR-based approach was capable of both predicting each phenotype individually and providing a metric of an overall healthy physiology. Importantly, the classification performance remained stable across the three testing methods for all the investigated phenotypes in this binary classification setting.

To further assess the efficacy of detecting each individual phenotype, we repeated the aforementioned analysis but removed the chain mechanism such that each phenotype was classified with an independent binary classifier (Figure S3). Without the chain, all classifications perform comparably well (Figure 2D vs. Figure S3). This similarity in predictive efficacy was expected for the binary classifications, as all predictions—whether chained or not—relied exclusively on information in the IR spectra. Hence, the classifiers did not require information about the predictions of previous classifiers as they were able to leverage all the information encoded in the IR spectrum. The only noticeable (albeit minor) drop in prediction efficacy was observed for prediabetes detection. This likely arose from the fact that prediabetes is an intermediate condition, and the control class included both type 2 diabetic and normal glucose tolerant (NGT) individuals. Therefore, we found that a single (linear) classifier struggled to single out the intermediate prediabetic cases, compared to combining multiple linear classifiers that have the capacity to single out NGT and type 2 diabetic individuals as well.

Phenotype-specific IR spectral signatures

Investigating the capacity to detect medically relevant phenotypes—both individually and simultaneously—raised the question of whether the molecular signature of individuals positive for a phenotype was characteristically distinct from those negative for it. To examine this, we calculated the difference between the mean IR molecular fingerprint of individuals positive for a phenotype and that of the remaining individuals' combined set of fingerprints encompassing the four remaining phenotypes (Figure 2E).

Differences in the molecular IR signatures of individuals bearing dyslipidemia, hypertension, and diabetic states showed very characteristic profiles with clear distinctions between one another. The signature of prediabetic individuals was revealed to share a very similar shape to that of individuals bearing type 2 diabetes—with only smaller variations in the magnitude of the differences. The latter finding is both promising and reassuring, given that prediabetes is an intermediate condition at a high risk of progressing into type 2 diabetes. Moreover, it was very reassuring to observe that the region with the most significant differences in the IR fingerprints of type 2 diabetic cases and controls was 1,000–1,180 cm^{-1}, with the peaks aligning with known spectral signatures of glucose.[46] When the fingerprints of the healthy individuals were compared to all the other individuals in the population, the differences revealed a shape that is similar to the inverse differences of all the other conditions studied—thereby supporting the aforementioned results. Crucially, the signatures of all phenotypes remained stable between the two sample sets, given the heavy overlap between the two curves depicted for each phenotype. Summarizing, these comparisons of spectral differences further corroborate the distinctive properties and reproducibility of the plasma-based IR fingerprinting approach.

Comparing the predictive value of IR fingerprints to clinical analytes

To examine the predictive value of IR molecular fingerprints in relation to standard clinical analytes, we repeated the multilabel classification but in a modified fashion. Rather than using IR fingerprints, we used a set of 12 commonly measured analytes from blood-based clinical chemistry and hematology analysis as the input features to make predictions (Figure S4). These analytes are widely used in blood cell counts, along with metabolomic and lipidomic panels.

Using the 12 clinical analytes as exclusive predictors of the studied phenotypes, this multilabel classification gained a slight advantage over the classification based only on IR fingerprints—with an exact match ratio of 0.57 and Hamming score of 0.70, compared to the previously mentioned exact match ratio of 0.51 and Hamming score of 0.68 (Figure 2C vs. Figure S4A). As the panels of clinical analytes included fasting glucose, hemoglobin A1c (HbA1c), low-density lipoprotein (LDL) cholesterol, high-density lipoprotein (HDL) cholesterol, and triglycerides, it was not surprising that an improvement in predictive capacity was observed when predicting type 2 diabetes and dyslipidemia as such clinical parameters are often used to clinically diagnose these conditions (Figure 2D vs. Figure S4B). For the condition that is not closely defined by such analytes, hypertension, the IR-based model outperformed the clinical-based model.

The involved costs of instrumentation and reagents, along with turnaround times, are a limiting factor for blood-based clinical laboratory testing. In contrast, the IR-based approach offers a reagent-free, one-shot measurement of bulk plasma, providing a comprehensive view of the sample's cross-molecular profile. This provides an efficient means of predicting the presence of health conditions, requiring minimal sample preparation, small sample volume, and short measurement time. A further highlight is that the IR-based approach is not limited to health conditions closely defined by conventional clinical laboratory parameters.

IR fingerprints are sensitive to anthropometric parameters

Gender, age, and body mass index (BMI) are risk factors for NCDs and chronic conditions.[47,48] Should plasma-based IR fingerprints encode the contributions of such anthropometric parameters, their effects on the IR fingerprints would potentially influence the predictions of models trained to detect clinical phenotypes.

To investigate the extent of their contributions, we first examined the capacity to predict the gender, age, and BMI of the sampled individuals using their IR molecular fingerprints (Figures S5A–S5C). Classifying gender was highly successful, achieving an AUC of 0.96. Estimating age with multivariate regression modeling was possible with an R^2 value of 0.58 and a root-mean-square error (RMSE) of 8.39 years. BMI was estimated with an R^2 value of 0.43 and an RMSE of 3.66 kg/m^2. These results demonstrate the unambiguous impact of gender, age, and BMI on blood-based IR molecular fingerprints, affecting any future diagnostic applications.

To further investigate their contributions independently of any possible health deviation, we performed the same analysis, but only on the healthy subset of individuals (Figures S5D–S5F). In this setting, classifying gender remained stable, with an AUC of 0.96. The efficacies of estimating age and BMI, however, were reduced to R^2 values of 0.52 and 0.37, respectively. Given that it was more challenging to estimate age and BMI in the healthy subset, this analysis suggested that NCDs or chronic conditions had an influence on the models estimating these anthropometric parameters. This supported the notion that the signals of aging and increased BMI are reflected in declining health states.

Prompted by these results, we further examined the capacity to detect each phenotype in a case-control setting—but classifying on samples of similar anthropometric distributions, rather than considering the populational distribution as previously. For each phenotype, we pair-matched a case individual to a control individual of similar gender, age, and BMI to minimize their possible contributions (Table S2). Using independent classification models, the AUCs ranged from 0.60 for prediabetes to 0.97 for dyslipidemia (Figure S6)—newly revealing that the contribution of these anthropometric parameters was higher for phenotypes with weaker disease signals.

Overall, we identified anthropometric parameters that significantly influence IR molecular fingerprints and, thereby, affect the phenotype classification models. These findings shall directly inform any future IR fingerprinting medical case evaluations as well as case-control clinical study designs.

IR fingerprints uncover shared pathophysiologies of MetS

MetS is a complex progressive condition characterized by a manifestation of interconnected metabolic abnormalities involving visceral obesity, hyperglycemia, raised blood pressure, raised triglycerides, and lowered HDL cholesterol (Figure 3A). Although its definition and pathogenesis are controversial,[49,50] MetS identifies individuals with shared pathophysiology—ones who are at risk of developing further health conditions, such as atherosclerotic cardiovascular disease, type 2 diabetes,[51] cerebrovascular events,[52] liver cancer,[53] and colorectal cancers.[54] Robust yet unsophisticated methods of identifying individuals with MetS would not only facilitate a better understanding of its pathophysiology but also enable efficient methods of stratifying populations into different levels of risk. This approach could further facilitate individualized pharmacologic and/or lifestyle interventions.[55,56]

Following the harmonized criteria of the International Diabetes Federation and the American Heart Association/National Heart, Lung, and Blood Institute,[57] MetS is present when at least three out of five aforementioned metabolic risk factors are present. As an early sign of MetS, one or two risk factors qualify as pre-MetS, a precursor condition that may represent the critical intermediate transition phase.[58,59] Breaking down the number of risk factors we observed in our two sample sets, we found that most individuals were at a pre-MetS stage (Figure 3B). At the pre-MetS stage, visceral obesity was the most prevalent risk factor, followed by hypertension and hyperglycemia (Table S3).

Employing the IR fingerprinting approach, we first systematically evaluated the extent to which we can single out individuals with varying numbers of metabolic risk factors from those with none (Figure 3C). We found that the classification efficacy increased when the number of metabolic risk factors increased

CellPress
OPEN ACCESS

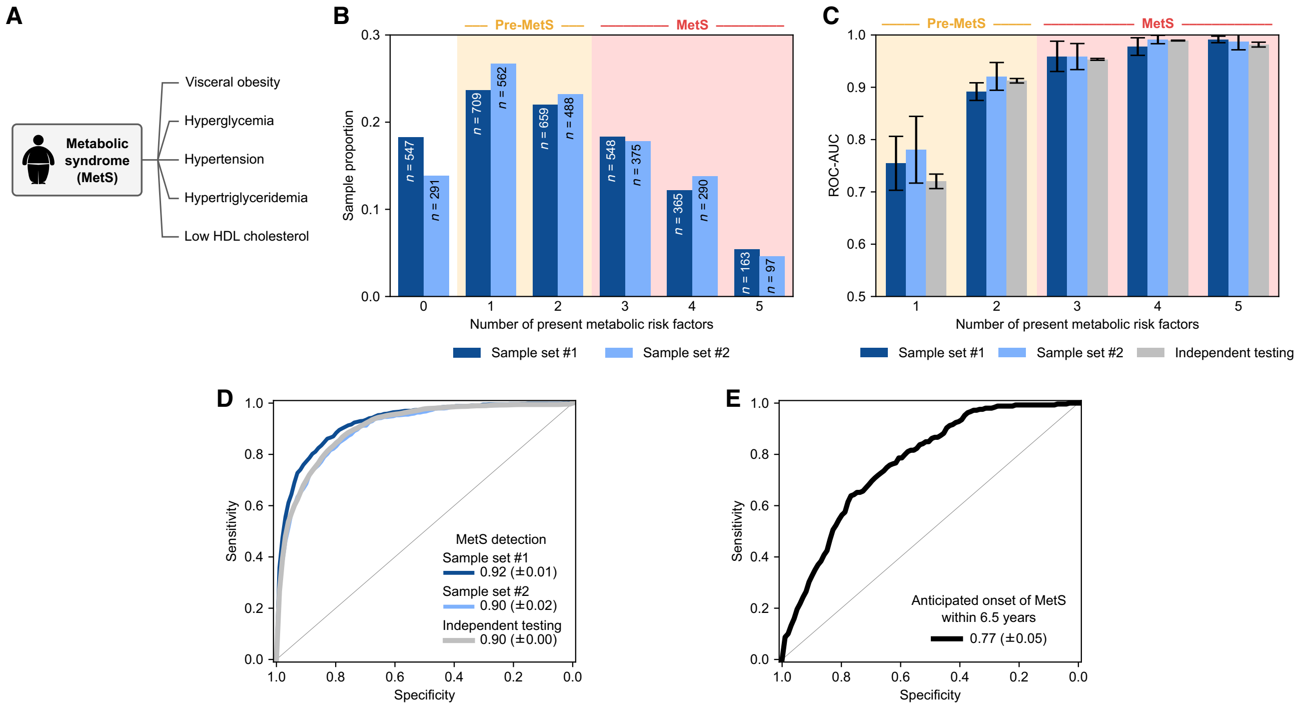

Figure 3. Detecting shared pathophysiologies through the metabolic syndrome

(A) Metabolic syndrome (MetS) was defined according to the harmonized criteria of the International Diabetes Federation (IDF) and American Heart Association/National Heart, Lung, and Blood Institute (AHA/NHLBI)—requiring individuals to have at least three of the five listed metabolic risk factors.

(B) Proportion of samples with different numbers of concurrent risk factors. The pure sample counts are listed on each corresponding bar. Samples with unknown values for any of the risk factors were excluded. Distributions of sample set #1 (dark blue bars) and sample set #2 (light blue bars) are depicted separately. See also Table S3 for a breakdown of risk factor prevalence.

(C) Binary classifiers were trained on the IR molecular fingerprints to distinguish individuals with one, two, three, and so on risk factors from those with none of the risk factors. Classifier validation was carried out as previously described—by cross-validating on sample set #1, cross-validating on sample set #2, and repeatedly training on a portion of sample set #1, testing on independent individuals from sample set #2. The mean area under the ROC curve is depicted for classifying each group, with the error bars showing the standard deviation of the AUCs across the validation sets.

(D) Binary classifiers were trained on the IR molecular fingerprints of the two population-based sample sets to distinguish those with MetS (i.e., having at least three risk factors) and those without MetS (i.e., having two or fewer risk factors). ROC curves are depicted on unseen test samples where classifier validation was carried out as previously described. The mean AUC is listed for each validation type, along with the standard deviation across the validation sets.

(E) Binary classification to forecast the future onset of MetS using IR molecular fingerprints. The classifier was trained on individuals from two groups: those who did not have MetS at baseline and developed it by the follow-up (n = 233), and those who did not have MetS at baseline and remained without it by the follow-up (n = 1,154). Classifier training and testing (via a 10-fold cross-validation) was performed exclusively on measurements from the baseline visit (sample set #1), while the medical records of the follow-up were examined to set the outcomes that describe whether the syndrome was developed (within an average of 6.5 follow-up years). The mean test AUC is listed along with its standard deviation.

within the group. Intriguingly, the approach had the capacity to single out pre-MetS cases with a high efficacy—achieving an AUC near 0.75 when identifying individuals with one risk factor and an AUC near 0.90 when identifying individuals with two concurrent risk factors. This observation underscored the classifier's ability to stratify individuals depending on their metabolic burden.

We further discovered the high capacity of identifying individuals with MetS, achieving an AUC near 0.90 (Figure 3D). This further highlighted the high efficacy of identifying those at a greater risk of developing more serious health conditions, as previously mentioned.

IR fingerprints forecast the development of MetS

In contrast to detecting the presence of MetS and metabolic risk factors, the longitudinal aspect in our dataset enabled us to also assess the capacity of forecasting the possible future development of MetS (Figure 3E). Within the study population, a subpopulation of 2,015 individuals participated in both baseline (sample set #1) and follow-up (sample set #2) samplings. Of these individuals, 1,387 did not have MetS at baseline, and among them, 233 developed MetS during the 6.5-year follow-up period. We constructed a predictive model that aimed to identify the individuals who transitioned from not having MetS at baseline but developed it in the follow-up sampling. As the control group, we used the 1,154 individuals who remained free of MetS throughout both samplings. Classifier training and testing were performed exclusively on the IR spectra of the baseline sampling (via 10-fold cross-validation). The binary outcomes, which denote whether each individual developed MetS during the follow-up, were determined by reviewing medical records from the follow-up. We found that the baseline measurements could effectively forecast

the future onset of MetS within the 6.5-year follow-up period, achieving an impressive AUC of 0.77 (Figure 3E). This AUC is comparable to a previously reported proteomics-based approach using 11 serum proteins, albeit over a 10-year follow-up period.[60]

Altogether, these results further highlighted the IR fingerprinting modality in screening scenarios as an efficient means of early detection to reduce the risk of developing further health conditions.

Interpreting IR spectral features through clinical analytes

To facilitate the medical utility of the approach, the nature of IR molecular fingerprints must be described in the context of known substances—e.g., clinical chemistry analytes. Since different biomolecules are composed of different atoms and chemical bonds, the concentrations of distinct molecular groups may correlate to different spectral regions at varying degrees. We therefore investigated how each spectral feature correlated with the concentrations of 12 commonly measured clinical lab analytes (Figure 4A).

Triglycerides and LDL cholesterol heavily overlapped in the regions of highest correlation with absorbance—with the regions of 1,725–1,750 cm^{-1} (C=O stretching modes of fatty acids[61]) and 2,800–2,975 cm^{-1} (CH_2 and CH_3 stretching modes of lipid[61]) reflecting the highest levels of positive correlations. Triglycerides also observed high levels of correlation with absorbance in the region of 1,100–1,275 cm^{-1} (C–O and C–O–C stretching of carbohydrates[61]). As LDL is the main source of cholesterol build-up, the total cholesterol also showed a very similar trend in correlation across the spectral range. HDL cholesterol, on the other hand, revealed a relatively weak correlation pattern compared to the aforementioned parameters. The remaining analytes revealed different patterns from that of the lipid profile. As HbA1c is a glycosylated form of hemoglobin localized within erythrocytes, it was reassuring to find that HbA1c and glucose concentrations correlated to similar wavenumbers. It was also expected to find that the measures of hematocrit (representing the volume of erythrocytes) and the number of erythrocytes had very similar patterns. Albumin, the most abundant protein in blood plasma, showed its strongest correlations at the so-called amide-I and -II bands, generally specific to proteins (between 1,550 and 1,700 cm^{-1}).[62] The remaining parameters observed relatively weak correlations across the spectral range.

These analyses were performed on the plasma IR fingerprints to broadly interpret the information encoded in the spectral positions by relating them to the concentrations of known clinical lab analytes. Although univariate analyses provide some insights by examining individual spectral features, they do not capture the full scope of molecular information in the IR fingerprints. Plasma, a highly complex biofluid, contains numerous molecular constituents that produce overlapping absorbance signals across different spectral features, potentially obscuring one another. To comprehensively understand the relationships between these clinical analyte concentrations and the information embedded in the IR fingerprints, it is crucial to consider the dependencies among spectral features. In the following subsection, we employ multivariate analyses to further examine relations between the clinical lab analytes and plasma IR fingerprints.

Information is shared between clinical analytes and IR fingerprints

To further investigate the extent to which the studied clinical analytes were encoded within the IR fingerprints, we applied multivariate regression analysis to quantify their individual concentrations using the plasma IR spectra (Figure 4B). Unlike univariate analysis, the interactions and dependencies among all spectral features are considered here, potentially capturing relations that may be obscured by molecular absorbance overlap.

We found that analytes relating to the lipid profile and glucose level were most accurately estimated, all with R^2 values ≥ 0.91 (Figure 4B). Surprisingly, the concentration of HDL cholesterol was estimated with a high efficacy despite its relatively weak correlations with individual spectral features as depicted in Figure 4A. This highlighted that, despite the molecular overshadowing effect that likely concealed its molecular contributions at individual spectral features, the HDL cholesterol content had sufficient contributions that were distributed across several spectral features—thereby enabling the analyte to be accurately estimated when spectral dependencies were considered.

Albumin, creatinine, and HbA1c revealed relatively weaker yet still significantly strong associations with the informational content encoded in the fingerprints. For albumin, the relatively weaker R^2 value of 0.58 sparked intriguing considerations—particularly due to its high abundance and its integral role in shaping the IR spectrum of blood plasma.[63] This prompted us to re-perform the analysis depicted in Figure 4B, but on non-normalized spectra (Figure S7B). Without the normalization, no statistically significant change was observed in the concentration prediction efficacy—neither for albumin nor for any other analyte. Although it was previously reported that IR spectroscopy using quantum cascade lasers has the means to quantify albumin,[64] our FTIR evaluations of plasma show differences here. This may be attributed to the fact that, in our setting, the clinical chemistry laboratory albumin concentrations were measured in serum (Methods), which may have contributed noise to the task of quantifying albumin from the plasma-based IR spectra. In addition, in this molecularly complex milieu, however, the presence of numerous coexisting plasma molecules might conceivably eclipse the distinctive spectral contributions of albumin—especially given that several other highly abundant proteins overlap in their regions of absorbance.[63]

As nearly half of the blood volume is made of erythrocytes, and as they serve as a reservoir of factors secreted in plasma, it was not surprising to find that their numbers could be estimated, to an extent, using IR molecular fingerprints of plasma. The parameters that were least reflected in the IR molecular fingerprints related to other types of blood cell counts—platelets and leukocytes. This indicated that neither their intracellular molecules nor their metabolic products strongly contribute to the extracellular composition of plasma and that the approach is not capable of specifically addressing their impacts on the chemistry of plasma (without any cellular enrichment and more dedicated analysis—e.g., immune cell profiling).

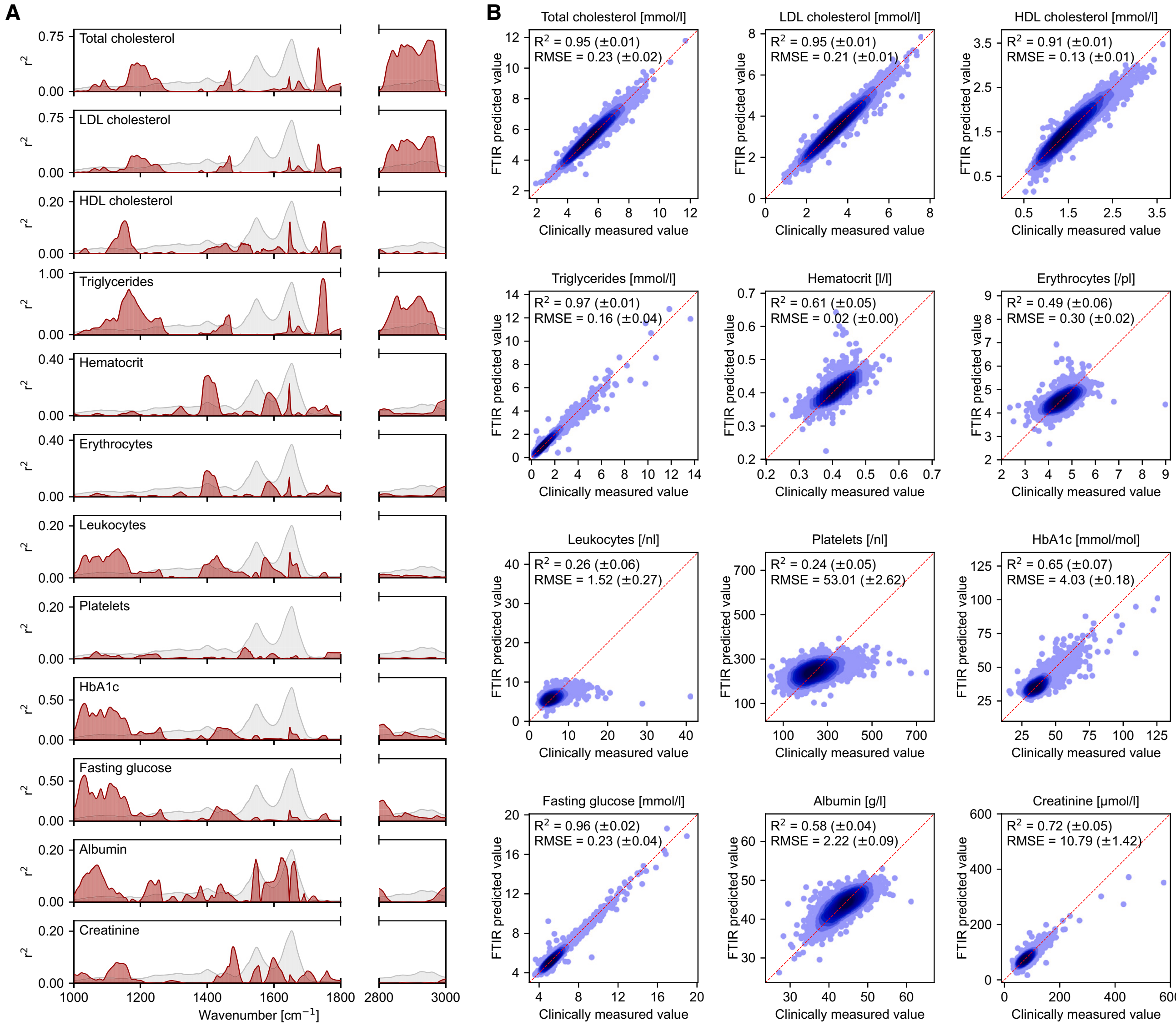

Figure 4. Classical clinical analytes reflected in IR molecular fingerprints

(A) Pearson correlation coefficient (red curves) between the concentrations of each clinical analyte and the absorbance at each wavenumber between 1,000 and 3,000 cm^{-1} for preprocessed spectra. The mean absorbance spectrum of all measured IR spectra (5,184 samples) is depicted in gray on each panel as a visual reference for the shape of the spectrum.

(B) Performance of quantitatively predicting clinical analytes using the preprocessed IR spectra of the measured population. Regression algorithms were trained for each parameter to capture the relations between the clinically measured values and the multivariate spectral features. The predicted values follow from the test sets of 10-fold cross-validations. Each point represents a measurement. The mean coefficient of determination (R^2) and the root-mean-square error (RMSE) are listed for each parameter, along with their standard deviations across the test splits. The diagonal (dashed red line) is a visual reference for a perfect fit. This same investigation was carried out on non-normalized spectra (Figure S7), where the univariate correlations (A) expectedly showed shifts in correlation trends while the efficacy of quantifying the concentration of each analyte (B) remained relatively unchanged.

In some instances, ratios between clinical analytes are utilized in medical diagnostics. For instance, ratios of triglycerides, LDL cholesterol, and total cholesterol to HDL cholesterol are often used for cardiovascular disease (CVD) risk assessments and have proven to be superior risk markers over individual analyte concentrations.[65] Since the IR molecular fingerprints are capable of estimating the concentrations of different parameters simultaneously, they are also informative to estimate ratios between them. To assess this, we calculated the ratios of triglycerides, LDL cholesterol, and total cholesterol to HDL cholesterol and studied the capacity to estimate their values (Figure S8). Thereby, we revealed that all three ratios were well estimated, each with an R^2 value near 0.92. This highlighted the potential of applying the approach to CVD risk assessments.

Altogether, it was evident that several distinct molecules and molecular groups correlate to the informational content of IR

fingerprints to a high degree despite the high complexity of blood plasma. IR fingerprint-based phenotype classification models can thus be linked to several relevant clinical lab parameters, providing a level of interpreting IR-based classifications in a known clinical context. Moreover, IR fingerprints present a viable alternative to traditional clinical laboratory methods for directly quantifying key clinical analytes, such as cholesterol and glucose levels, in routine clinical lab analysis. This provides an efficient, reagent-free means of simultaneously quantifying several analytes in a one-shot measurement.

DISCUSSION

The value of high-throughput molecular profiling is highest when molecular alterations have the capacity to define several medical phenotypes. The framework for IR fingerprinting to possibly fuel medical screening targeting the diversity of common conditions and their combinations within a naturally variable population has not been provided before.[37,38,66] It was yet to be comprehensively proven whether IR fingerprinting could facilitate the robustness required for population-wide screening or whether it carries any capacity to capture the reality of complex multimorbid prevalence.

Combining IR spectroscopy with machine learning, our study demonstrated that IR molecular fingerprints (1) have the capacity to simultaneously detect and distinguish between common health phenotypes, providing a basis for multi-task phenotype detection; (2) can detect health-to-disease intermediate medical phenotypes (e.g., prediabetes and (pre-)MetS) that are of great value to screen and stratify populations for risk of developing further health conditions and intervene timely; (3) can forecast the future onset of clinically relevant health disorders, showcasing the approach's potential as an early detection approach; (4) strongly correlate to the concentrations of several clinical chemistry analytes; (5) reflect the physiological contributions of anthropometric parameters (gender, age, and BMI); and (6) are robust to variations in sample handling, storage time, and measurement regime differences on a multi-year scale. The approach is powerful for high-throughput population-scale screening. Specifically, for describing common, chronic health state phenotypes with a single cost-effective measurement that requires no preanalytical sample preparation or *a priori* molecular knowledge.

Health is multidimensional and individually heterogeneous, and parametrizing it is non-trivial. As complex clinical traits may be effectively probed systemically,[67] system-level cross-molecular IR fingerprints provide a new *in vitro* diagnostic advantage here. The impact of the presented paradigm, beyond clinical study scenarios, is manyfold. Especially as, in any adult population, the majority of individuals with identified health aberrations will likely have multiple coexisting conditions, and various shared disease mechanisms can ideally be targeted via lifestyle or pharmacological interventions.[40,55,68] Thus, screening avenues to efficiently discern between different multimorbid states are essential. Multimorbid states have been initially thought of as a collection of independent ailments and are still often measured separately. Given the direct coupling between many phenotypes (e.g., metabolic abnormalities and cardiovascular conditions), informing measures on multimorbidity could advance therapeutic strategies and help assess and stratify risk levels. Importantly, the classifier detecting healthy individuals provided a very high prediction capacity (AUC of 0.90 when independently tested). This demonstrates that the approach is well suited to single out individuals in their healthspan (the period of life free from disease).

Beyond the phenotypes investigated here, the approach can serve as a platform that can be extended to screen and stratify for a variety of other health conditions. It may also be suitable for conditions of either unknown molecular origins or ones without knowledge of the underlying multi-molecular changes. To better understand and capture sub-clinical, intermediate phenotypes, we showcased how IR fingerprinting could facilitate the isolation of actionable at-risk phenotypes. Specifically, reporting on prediabetes could present an intervention that may prevent or delay the onset of type 2 diabetes that would, in turn, reduce the incidence of atherosclerotic CVD, kidney disease, vascular dementia, and Alzheimer's disease.[69–72] Although methods for diagnosing prediabetes and type 2 diabetes are available,[73,74] tools to identify individuals at risk for type 2 diabetes that could guide interventions are still lacking.[75] The second example is the capacity of IR fingerprinting to detect pre-MetS, a precondition and risk factor that may contribute to the evolution of further diseases. Thus, the presented framework is valuable for dynamic risk predictions across a variety of phenotypes as well as to capture combined conditions and potentially forecast their future onset—especially given its minimally invasive nature, ease of measurement, and low cost of the approach.

Previous clinical spectroscopy studies typically relied on rather small patient cohorts, hindering the potential of developing IR-based diagnostic models that adeptly generalize to larger populations.[38] Involving two datasets, from samples collected at two different times as well as experimentally measured years apart, we not only cross-validated the outcomes of each sample set separately but also further validated the robustness of our findings in a more realistic validation setting. Specifically, we were capable of training a classifier on samples from individuals collected at a point in time and testing this classifier on a different group of individuals that were measured years apart. Such a validation approach limited the potential to overestimate the clinical efficacy of our developed detection models. Additionally, the analyzed study paradigm reduced the possibility of patient selection bias since the individuals assessed were from a naturally heterogeneous population-based study involving a variety of potential conditions and health states—thus reflecting a realistic demographic distribution and true disease prevalence.

Limitations of the study

Critically seen, the generalizations of our machine learning applications may be limited to the studied population—where many individuals likely share similar genetic backgrounds and lifestyles.[39] To investigate the extent of this limitation, the models must be further tested on independent sets of ethnically diverse individuals. Secondly, the presented metrics of model validation considered the ground truth to be the health characterizations determined using existing clinical evaluations and may have been limited by the efficiency of the clinical analytical procedures

performed. For instance, diagnosing prediabetes and type 2 diabetes relied on an oral glucose tolerance test. Although commonly applied, this diagnostic approach is influenced by a multitude of physiological fluctuations.[74,76] Finally, some of our analyses treated two time-separated samplings of partially overlapping individuals as two cross-sectional sample sets. This may partially explain the agreement between the cross-validations performed on the two sample sets. Additional testing on cohorts from fully independent clinical studies would provide an even more definitive verification of our findings.

Conclusions

The human body is a dynamic and complex system—with a multitude of ongoing biochemical processes driven by genetics, individual lifestyles, and aging.[77] The role of IR fingerprints to predict future disease onset and regression will need to be further assessed in additional longitudinal analyses that will be important to even more accurately capture and model (sub)clinical phenotypes, especially in light of the heterogeneity of person-specific healthspans. The concept of within-person stability of IR fingerprints has been revealed,[78] and we also demonstrated the underlying concept that time-tracking of IR fingerprints enhances phenotype detection.[79] However, it still remains to be proven whether decades-long health state trajectories can be decoded with IR fingerprinting.

Altogether, this study sets a framework with analytical validity and clinical utility that could reduce and streamline clinical operations, improve sample turnarounds, accelerate time to treatment for a variety of medical conditions, and risk stratify populations. We identify the value of IR fingerprinting when combined with machine learning as a multi-phenotyping modality able to fuel medical screening at very affordable costs and demonstrated potential to complement health checkups. If further developed and independently validated, the approach has the potential to provide actionable information to support and empower reliable clinical decisions while conserving resources.

STAR★METHODS

Detailed methods are provided in the online version of this paper and include the following:

- KEY RESOURCES TABLE
- RESOURCE AVAILABILITY
  - Lead contact
  - Materials availability
  - Data and code availability
- EXPERIMENTAL MODEL AND SUBJECT DETAILS
  - Study design and sample collection
  - Phenotype definitions, biochemical and hematological analysis
  - Exclusion of samples
- METHOD DETAILS
  - Sample preparation and FTIR measurements
  - Measurement preprocessing
- QUANTIFICATION AND STATISTICAL ANALYSIS
  - Classification analysis
  - Validation of multilabel and binary classifications
  - Multilabel classification metrics
  - Regression analysis
  - Feature selection analysis
  - Analysis software

SUPPLEMENTAL INFORMATION

Supplemental information can be found online at https://doi.org/10.1016/j.xcrm.2024.101625.

ACKNOWLEDGMENTS

This work was supported by LMU Munich, Centre for Advanced Laser Applications (CALA). The KORA study was initiated and financed by the Helmholtz Zentrum München – German Research Center for Environmental Health, which is funded by the German Federal Ministry of Education and Research (BMBF) and the State of Bavaria. Data collection in the KORA study is done in cooperation with the University Hospital of Augsburg. We thank Ferenc Krausz for the research environment, Sigrid Auweter for her support in initiating this study, and Jacqueline Hermann for her support throughout the study.

AUTHOR CONTRIBUTIONS

M.Ž. designed, initiated, and supervised the study; T.E., C.L., K.V.K., and M.Ž. designed the analytical framework; T.E. defined the data analysis methodology and performed all computational analyses, data visualizations, and data interpretations; T.E. and C.L. coordinated the data acquisition from the KORA study; B.L. and A.P. oversaw, organized, and coordinated the KORA study; C.L., D.M., and V.Z. performed the experimental measurements; F.F. supervised and contributed to the experimental measurements; K.V.K., L.R., M.H., and L.V. provided methodological and statistical advice on the data analysis; T.E. and M.Ž. wrote the manuscript; all authors contributed to revising the manuscript; and all authors approve the manuscript.

DECLARATION OF INTERESTS

The authors declare no competing interests.

Received: October 11, 2023
Revised: April 19, 2024
Accepted: June 7, 2024
Published: June 28, 2024

REFERENCES

1. Shilo, S., Rossman, H., and Segal, E. (2020). Axes of a revolution: challenges and promises of big data in healthcare. Nat. Med. *26*, 29–38.
2. Hasin, Y., Seldin, M., and Lusis, A. (2017). Multi-omics approaches to disease. Genome Biol. *18*, 83.
3. Wright, J.T., and Herzberg, M.C. (2021). Science for the next century: Deep phenotyping. J. Dent. Res. *100*, 785–789.
4. Duarte, T.T., and Spencer, C.T. (2016). Personalized proteomics: The future of precision medicine. Proteomes *4*, 29.
5. Zhi, M., Ding, E.L., Theisen-Toupal, J., Whelan, J., and Arnaout, R. (2013). The landscape of inappropriate laboratory testing: A 15-year meta-analysis. PLoS One *8*, e78962.
6. Kogan, A., Peleg, M., Tu, S.W., Allon, R., Khaitov, N., and Hochberg, I. (2020). Towards a goal-oriented methodology for clinical-guideline-based management recommendations for patients with multimorbidity: Gocom and its preliminary evaluation. J. Biomed. Inf. *112*, 103587.
7. Shen, X., Kellogg, R., Panyard, D.J., Bararpour, N., Castillo, K.E., Lee-McMullen, B., Delfarah, A., Ubellacker, J., Ahadi, S., Rosenberg-Hasson, Y., et al. (2023). Multi-omics microsampling for the profiling of lifestyle-associated changes in health. Nat. Biomed. Eng. *8*, 11–29.
8. Emwas, A.-H.M. (2015). The strengths and weaknesses of NMR spectroscopy and mass spectrometry with particular focus on metabolomics research. In Methods in Molecular Biology (Springer), pp. 161–193.
9. Seger, C., and Salzmann, L. (2020). After another decade: LC–MS/MS became routine in clinical diagnostics. Clin. Biochem. *82*, 2–11.

10. Geyer, P.E., Voytik, E., Treit, P.V., Doll, S., Kleinhempel, A., Niu, L., Müller, J.B., Buchholtz, M.-L., Bader, J.M., Teupser, D., et al. (2019). Plasma proteome profiling to detect and avoid sample-related biases in biomarker studies. EMBO Mol. Med. *11*, e10427.
11. Buergel, T., Steinfeldt, J., Ruyoga, G., Pietzner, M., Bizzarri, D., Vojinovic, D., Upmeier zu Belzen, J., Loock, L., Kittner, P., Christmann, L., et al. (2022). Metabolomic profiles predict individual multidisease outcomes. Nat. Med. *28*, 2309–2320.
12. Griffiths, P.R., De Haseth, J.A., and Winefordner, J.D. (2007). Fourier Transform Infrared Spectrometry. Chemical Analysis: A Series of Monographs on Analytical Chemistry and its Applications, Second Edition (Wiley-Blackwell).
13. Movasaghi, Z., Rehman, S., and ur Rehman, I. (2008). Fourier transform infrared (FTIR) spectroscopy of biological tissues. Appl. Spectrosc. Rev. *43*, 134–179.
14. Dębska, B., and Guzowska-Świder, B. (2000). Fuzzy definition of molecular fragments in chemical structures. J. Chem. Inf. Comput. Sci. *40*, 325–329.
15. Miller, L.M., and Smith, R.J. (2005). Synchrotrons versus globars, point-detectors versus focal plane arrays: Selecting the best source and detector for specific infrared microspectroscopy and imaging applications. Vib. Spectrosc. *38*, 237–240.
16. Pupeza, I., Huber, M., Trubetskov, M., Schweinberger, W., Hussain, S.A., Hofer, C., Fritsch, K., Poetzlberger, M., Vamos, L., Fill, E., et al. (2020). Field-resolved infrared spectroscopy of biological systems. Nature *577*, 52–59.
17. Kowligy, A.S., Timmers, H., Lind, A.J., Elu, U., Cruz, F.C., Schunemann, P.G., Biegert, J., and Diddams, S.A. (2019). Infrared electric field sampled frequency comb spectroscopy. Sci. Adv. *5*, eaaw8794.
18. Villares, G., Hugi, A., Blaser, S., and Faist, J. (2014). Dual-comb spectroscopy based on quantum-cascade-laser frequency combs. Nat. Commun. *5*, 5192.
19. Schwaighofer, A., Montemurro, M., Freitag, S., Kristament, C., Culzoni, M.J., and Lendl, B. (2018). Beyond Fourier transform infrared spectroscopy: External cavity quantum cascade laser-based mid-infrared transmission spectroscopy of proteins in the amide I and amide II region. Anal. Chem. *90*, 7072–7079.
20. Baker, M.J., Trevisan, J., Bassan, P., Bhargava, R., Butler, H.J., Dorling, K.M., Fielden, P.R., Fogarty, S.W., Fullwood, N.J., Heys, K.A., et al. (2014). Using Fourier transform IR spectroscopy to analyze biological materials. Nat. Protoc. *9*, 1771–1791.
21. Huber, M., Kepesidis, K.V., Voronina, L., Fleischmann, F., Fill, E., Hermann, J., Koch, I., Milger-Kneidinger, K., Kolben, T., Schulz, G.B., et al. (2021). Infrared molecular fingerprinting of blood-based liquid biopsies for the detection of cancer. eLife *10*, e68758.
22. Baker, M.J., Hussain, S.R., Lovergne, L., Untereiner, V., Hughes, C., Lukaszewski, R.A., Thiéfin, G., and Sockalingum, G.D. (2016). Developing and understanding biofluid vibrational spectroscopy: a critical review. Chem. Soc. Rev. *45*, 1803–1818.
23. Martin, F.L., Kelly, J.G., Llabjani, V., Martin-Hirsch, P.L., Patel, I.I., Trevisan, J., Fullwood, N.J., and Walsh, M.J. (2010). Distinguishing cell types or populations based on the computational analysis of their infrared spectra. Nat. Protoc. *5*, 1748–1760.
24. Petrich, W., Lewandrowski, K.B., Muhlestein, J.B., Hammond, M.E.H., Januzzi, J.L., Lewandrowski, E.L., Pearson, R.R., Dolenko, B., Früh, J., Haass, M., et al. (2009). Potential of mid-infrared spectroscopy to aid the triage of patients with acute chest pain. Analyst *134*, 1092–1098.
25. Li, Y., Guo, L., Li, L., Yang, C., Guang, P., Huang, F., Chen, Z., Wang, L., and Hu, J. (2020). Early diagnosis of type 2 diabetes based on near-infrared spectroscopy combined with machine learning and aquaphotomics. Front. Chem. *8*, 580489.
26. de Souza, N.M.P., Machado, B.H., Koche, A., da Silva Furtado, L.B.F., Becker, D., Corbellini, V.A., and Rieger, A. (2023). Discrimination of dyslipidemia types with ATR-FTIR spectroscopy and chemometrics associated with multivariate analysis of the lipid profile, anthropometric, and pro-inflammatory biomarkers. Clin. Chim. Acta *540*, 117231.
27. Bhargava, R. (2023). Digital histopathology by infrared spectroscopic imaging. Annu. Rev. Anal. Chem. *16*, 205–230.
28. Bellisola, G., and Sorio, C. (2012). Infrared spectroscopy and microscopy in cancer research and diagnosis. Am. J. Cancer Res. *2*, 1–21.
29. Kazarian, S.G., and Chan, K.L.A. (2013). ATR-FTIR spectroscopic imaging: recent advances and applications to biological systems. Analyst *138*, 1940.
30. Townsend, D., Miljković, M., Bird, B., Lenau, K., Old, O., Almond, M., Kendall, C., Lloyd, G., Shepherd, N., Barr, H., et al. (2015). Infrared micro-spectroscopy for cyto-pathological classification of esophageal cells. Analyst *140*, 2215–2223.
31. De Bruyne, S., Speeckaert, M.M., and Delanghe, J.R. (2017). Applications of Mid-infrared Spectroscopy in the Clinical Laboratory Setting. Crit. Rev. Clin. Lab. Sci. *55*, 1–20.
32. Chen, X., Shu, W., Zhao, L., and Wan, J. (2022). Advanced mass spectrometric and spectroscopic methods coupled with machine learning for in vitro diagnosis. View *4*, 20220038.
33. Silveira, L., Borges, R.d.C.F., Navarro, R.S., Giana, H.E., Zângaro, R.A., Pacheco, M.T.T., and Fernandes, A.B. (2017). Quantifying glucose and lipid components in human serum by Raman spectroscopy and multivariate statistics. Laser Med. Sci. *32*, 787–795.
34. Perez-Guaita, D., Ventura-Gayete, J., Pérez-Rambla, C., Sancho-Andreu, M., Garrigues, S., and de la Guardia, M. (2012). Protein determination in serum and whole blood by attenuated total reflectance infrared spectroscopy. Anal. Bioanal. Chem. *404*, 649–656.
35. Perez-Guaita, D., Ventura-Gayete, J., Pérez-Rambla, C., Sancho-Andreu, M., Garrigues, S., and de la Guardia, M. (2013). Evaluation of infrared spectroscopy as a screening tool for serum analysis. Microchem. J. *106*, 202–211.
36. Anderson, D.J., Anderson, R.G., Moug, S.J., and Baker, M.J. (2020). Liquid biopsy for cancer diagnosis using vibrational spectroscopy: systematic review. BJS Open *4*, 554–562.
37. Finlayson, D., Rinaldi, C., and Baker, M.J. (2019). Is infrared spectroscopy ready for the clinic? Anal. Chem. *91*, 12117–12128.
38. Cameron, J.M., Rinaldi, C., Rutherford, S.H., Sala, A., G Theakstone, A., and Baker, M.J. (2022). Clinical spectroscopy: Lost in translation? Appl. Spectrosc. *76*, 393–415.
39. Holle, R., Happich, M., Löwel, H., and Wichmann, H. (2005). KORA - a research platform for population based health research. Gesundheitswesen *67*, 19–25.
40. Smith, S.M., Wallace, E., O'Dowd, T., and Fortin, M. (2021). Interventions for improving outcomes in patients with multimorbidity in primary care and community settings: a systematic review. Syst. Rev. *10*, 271.
41. Jakovljević, M., and Ostojić, L. (2013). Comorbidity and multimorbidity in medicine today: challenges and opportunities for bringing separated branches of medicine closer to each other. Psychiatr. Danub. *25*, 18–28.
42. Read, J., Pfahringer, B., Holmes, G., and Frank, E. (2011). Classifier chains for multi-label classification. Mach. Learn. *85*, 333–359.
43. Tsoumakas, G., and Katakis, I. (2007). Multi-label classification. Int. J. Data Warehous. Min. *3*, 1–13.
44. Godbole, S., and Sarawagi, S. (2004). Discriminative methods for multi-labeled classification. In Advances in Knowledge Discovery and Data Mining (Springer Berlin Heidelberg), pp. 22–30.
45. Sorower, M.S. (2010). A Literature Survey on Algorithms for Multi-Label Learning*18* (Oregon State University).
46. Kasahara, R., Kino, S., Soyama, S., and Matsuura, Y. (2018). Noninvasive glucose monitoring using mid-infrared absorption spectroscopy based on a few wavenumbers. Biomed. Opt Express *9*, 289–302.

CellPress
OPEN ACCESS

47. Uijl, A., Koudstaal, S., Direk, K., Denaxas, S., Groenwold, R.H.H., Banerjee, A., Hoes, A.W., Hemingway, H., and Asselbergs, F.W. (2019). Risk factors for incident heart failure in age- and sex-specific strata: a population-based cohort using linked electronic health records. Eur. J. Heart Fail. *21*, 1197–1206.
48. Meisinger, C., Thorand, B., Schneider, A., Stieber, J., Döring, A., and Löwel, H. (2002). Sex differences in risk factors for incident type 2 diabetes mellitus. Arch. Intern. Med. *162*, 82.
49. Grundy, S.M., Cleeman, J.I., Daniels, S.R., Donato, K.A., Eckel, R.H., Franklin, B.A., Gordon, D.J., Krauss, R.M., Savage, P.J., Smith, S.C., et al. (2005). Diagnosis and management of the metabolic syndrome. Circulation *112*, 2735–2752.
50. Grundy, S.M. (2016). Metabolic syndrome update. Trends Cardiovasc. Med. *26*, 364–373.
51. Sattar, N., McConnachie, A., Shaper, A.G., Blauw, G.J., Buckley, B.M., de Craen, A.J., Ford, I., Forouhi, N.G., Freeman, D.J., Jukema, J.W., et al. (2008). Can metabolic syndrome usefully predict cardiovascular disease and diabetes? outcome data from two prospective studies. Lancet *371*, 1927–1935.
52. Wang, Y., Li, J., Zheng, X., Jiang, Z., Hu, S., Wadhera, R.K., Bai, X., Lu, J., Wang, Q., Li, Y., et al. (2018). Risk factors associated with major cardiovascular events 1 year after acute myocardial infarction. JAMA Netw. Open *1*, e181079.
53. Esposito, K., Chiodini, P., Colao, A., Lenzi, A., and Giugliano, D. (2012). Metabolic syndrome and risk of cancer. Diabetes Care *35*, 2402–2411.
54. Chen, H., Zheng, X., Zong, X., Li, Z., Li, N., Hur, J., Fritz, C.D., Chapman, W., Jr., Nickel, K.B., Tipping, A., et al. (2021). Metabolic syndrome, metabolic comorbid conditions and risk of early-onset colorectal cancer. Gut *70*, 1147–1154.
55. Huang, P.L. (2009). A comprehensive definition for metabolic syndrome. Dis. Model. Mech. *2*, 231–237.
56. Monnerie, S., Comte, B., Ziegler, D., Morais, J.A., Pujos-Guillot, E., and Gaudreau, P. (2020). Metabolomic and lipidomic signatures of metabolic syndrome and its physiological components in adults: A systematic review. Sci. Rep. *10*, 669.
57. Alberti, K., Eckel, R.H., Grundy, S.M., Zimmet, P.Z., Cleeman, J.I., Donato, K.A., Fruchart, J.-C., James, W.P.T., Loria, C.M., and Smith, S.C. (2009). Harmonizing the metabolic syndrome. Circulation *120*, 1640–1645.
58. de las Fuentes, L., Brown, A.L., Mathews, S.J., Waggoner, A.D., Soto, P.F., Gropler, R.J., and Davila-Roman, V.G. (2006). Metabolic syndrome is associated with abnormal left ventricular diastolic function independent of left ventricular mass. Eur. Heart J. *28*, 553–559.
59. Chen, Y., Xu, W., Zhang, W., Tong, R., Yuan, A., Li, Z., Jiang, H., Hu, L., Huang, L., Xu, Y., et al. (2023). Plasma metabolic fingerprints for large-scale screening and personalized risk stratification of metabolic syndrome. Cell Rep. Med. *4*, 101109.
60. Cai, X., Xue, Z., Zeng, F.-F., Tang, J., Yue, L., Wang, B., Ge, W., Xie, Y., Miao, Z., Gou, W., et al. (2023). Population serum proteomics uncovers a prognostic protein classifier for metabolic syndrome. Cell Rep. Med. *4*, 101172.
61. Lacombe, C., Untereiner, V., Gobinet, C., Zater, M., Sockalingum, G.D., and Garnotel, R. (2015). Rapid screening of classic galactosemia patients: a proof-of-concept study using high-throughput FTIR analysis of plasma. Analyst *140*, 2280–2286.
62. Barth, A. (2007). Infrared spectroscopy of proteins. Biochim. Biophys. Acta *1767*, 1073–1101.
63. Voronina, L., Leonardo, C., Mueller-Reif, J.B., Geyer, P.E., Huber, M., Trubetskov, M., Kepesidis, K.V., Behr, J., Mann, M., Krausz, F., and Žigman, M. (2021). Molecular origin of blood-based infrared spectroscopic fingerprints. Angew. Chem., Int. Ed. Engl. *60*, 17060–17069.
64. Brandstetter, M., Sumalowitsch, T., Genner, A., Posch, A.E., Herwig, C., Drolz, A., Fuhrmann, V., Perkmann, T., and Lendl, B. (2013). Reagent-free monitoring of multiple clinically relevant parameters in human blood plasma using a mid-infrared quantum cascade laser based sensor system. Analyst *138*, 4022–4028.
65. Millán, J., Pintó, X., Muñoz, A., Zúñiga, M., Rubiés-Prat, J., Pallardo, L.F., Masana, L., Mangas, A., Hernández-Mijares, A., González-Santos, P., et al. (2009). Lipoprotein ratios: physiological significance and clinical usefulness in cardiovascular prevention. Vasc. Health Risk Manag. *5*, 757–765.
66. Su, K.-Y., and Lee, W.-L. (2020). Fourier transform infrared spectroscopy as a cancer screening and diagnostic tool: A review and prospects. Cancers *12*, 115.
67. Civelek, M., and Lusis, A.J. (2013). Systems genetics approaches to understand complex traits. Nat. Rev. Genet. *15*, 34–48.
68. Li, X., Ploner, A., Wang, Y., Zhan, Y., Pedersen, N.L., Magnusson, P.K., Jylhävä, J., and Hägg, S. (2021). Clinical biomarkers and associations with healthspan and lifespan: Evidence from observational and genetic data. EBioMedicine *66*, 103318.
69. Afkarian, M., Sachs, M.C., Kestenbaum, B., Hirsch, I.B., Tuttle, K.R., Himmelfarb, J., and de Boer, I.H. (2013). Kidney disease and increased mortality risk in type 2 diabetes. J. Am. Soc. Nephrol. *24*, 302–308.
70. Peila, R., Rodriguez, B.L., and Launer, L.J.; Honolulu-Asia Aging Study (2002). Type 2 diabetes, APOE gene, and the risk for dementia and related pathologies: The Honolulu-Asia Aging Study. Diabetes *51*, 1256–1262.
71. Poznyak, A., Grechko, A.V., Poggio, P., Myasoedova, V.A., Alfieri, V., and Orekhov, A.N. (2020). The diabetes mellitus-atherosclerosis connection: The role of lipid and glucose metabolism and chronic inflammation. Int. J. Mol. Sci. *21*, 1835.
72. Xu, W., Qiu, C., Winblad, B., and Fratiglioni, L. (2007). The effect of borderline diabetes on the risk of dementia and Alzheimer's disease. Diabetes *56*, 211–216.
73. US Preventive Services Task Force (2021). Screening for Prediabetes and Type 2 Diabetes: US Preventive Services Task Force Recommendation Statement. JAMA *326*, 736–743.
74. Bogdanet, D., O'Shea, P., Lyons, C., Shafat, A., and Dunne, F. (2020). The oral glucose tolerance test—is it time for a change?—a literature review with an emphasis on pregnancy. J. Clin. Med. *9*, 3451.
75. Edlitz, Y., and Segal, E. (2022). Prediction of type 2 diabetes mellitus onset using logistic regression-based scorecards. eLife *11*, e71862.
76. Shilo, S., Keshet, A., Rossman, H., Godneva, A., Talmor-Barkan, Y., Aviv, Y., and Segal, E. (2024). Continuous glucose monitoring and intrapersonal variability in fasting glucose. Nat. Med. *30*, 1424–1431.
77. Rutledge, J., Oh, H., and Wyss-Coray, T. (2022). Measuring biological age using omics data. Nat. Rev. Genet. *23*, 715–727.
78. Huber, M., Kepesidis, K.V., Voronina, L., Božić, M., Trubetskov, M., Harbeck, N., Krausz, F., and Žigman, M. (2021). Stability of person-specific blood-based infrared molecular fingerprints opens up prospects for health monitoring. Nat. Commun. *12*, 1511.
79. Eissa, T., Kepesidis, K.V., Zigman, M., and Huber, M. (2023). Limits and prospects of molecular fingerprinting for phenotyping biological systems revealed through in silico modeling. Anal. Chem. *95*, 6523–6532.
80. Harris, C.R., Millman, K.J., van der Walt, S.J., Gommers, R., Virtanen, P., Cournapeau, D., Wieser, E., Taylor, J., Berg, S., Smith, N.J., et al. (2020). Array programming with NumPy. Nature *585*, 357–362.
81. McKinney, W. (2010). Data Structures for Statistical Computing in Python. In Proceedings of the 9th Python in Science Conference (SciPy), pp. 56–61.
82. Virtanen, P., Gommers, R., Oliphant, T.E., Haberland, M., Reddy, T., Cournapeau, D., Burovski, E., Peterson, P., Weckesser, W., Bright, J., et al. (2020). SciPy 1.0: fundamental algorithms for scientific computing in Python. Nat. Methods *17*, 261–272.
83. Pedregosa, F., Varoquaux, G., Gramfort, A., Michel, V., Thirion, B., Grisel, O., Blondel, M., Prettenhofer, P., Weiss, R., Dubourg, V., et al. (2011).

Scikit-learn: Machine learning in Python. J. Mach. Learn. Res. *12*, 2825–2830.

84. Hunter, J.D. (2007). Matplotlib: A 2D graphics environment. Comput. Sci. Eng. *9*, 90–95.

85. Sujana, C., Seissler, J., Jordan, J., Rathmann, W., Koenig, W., Roden, M., Mansmann, U., Herder, C., Peters, A., Thorand, B., and Then, C. (2020). Associations of cardiac stress biomarkers with incident type 2 diabetes and changes in glucose metabolism: KORA F4/FF4 study. Cardiovasc. Diabetol. *19*, 178.

86. Wichmann, H.-E., Gieger, C., and Illig, T. (2005). KORA-gen - resource for population genetics, controls and a broad spectrum of disease phenotypes. Gesundheitswesen *67*, 26–30.

87. Rathmann, W., Haastert, B., Icks, A., Löwel, H., Meisinger, C., Holle, R., and Giani, G. (2003). High prevalence of undiagnosed diabetes mellitus in southern germany: Target populations for efficient screening. the KORA survey 2000. Diabetologia *46*, 182–189.

88. Jellinger, P.S., Handelsman, Y., Rosenblit, P.D., Bloomgarden, Z.T., Fonseca, V.A., Garber, A.J., Grunberger, G., Guerin, C.K., Bell, D.S., Mechanick, J.I., et al. (2017). American association of clinical endocrinologists and American college of endocrinology guidelines for management of dyslipidemia and prevention of cardiovascular disease. Endocr. Pract. *23*, 1–87.

89. Nantsupawat, N., Booncharoen, A., Wisetborisut, A., Jiraporncharoen, W., Pinyopornpanish, K., Chutarattanakul, L., and Angkurawaranon, C. (2019). Appropriate total cholesterol cut-offs for detection of abnormal LDL cholesterol and non-HDL cholesterol among low cardiovascular risk population. Lipids Health Dis. *18*, 28.

90. Meisinger, C., Heier, M., Völzke, H., Löwel, H., Mitusch, R., Hense, H.-W., and Lüdemann, J. (2006). Regional disparities of hypertension prevalence and management within germany. J. Hypertens. *24*, 293–299.

91. World Health Organization (1999). Definition, diagnosis and classification of diabetes mellitus and its complications: report of a WHO consultation. part 1, diagnosis and classification of diabetes mellitus.

92. Elhadad, M.A., Wilson, R., Zaghlool, S.B., Huth, C., Gieger, C., Grallert, H., Graumann, J., Rathmann, W., Koenig, W., Sinner, M.F., et al. (2021). Metabolic syndrome and the plasma proteome: from association to causation. Cardiovasc. Diabetol. *20*, 111.

93. Meisinger, C., Rückert, I., Stöckl, D., Thorand, B., Peters, A., Kowall, B., and Rathmann, W. (2014). Hematological parameters and prediabetes and diabetes in adults from the general population: A cross-sectional study. J. Diabetes Metabol. *05*.

94. Riedl, A., Wawro, N., Gieger, C., Meisinger, C., Peters, A., Roden, M., Kronenberg, F., Herder, C., Rathmann, W., Völzke, H., et al. (2018). Identification of comprehensive metabotypes associated with cardiometabolic diseases in the population-based KORA study. Mol. Nutr. Food Res. *62*, 1800117.

95. Lau, L.H.Y., Nano, J., Cecil, A., Schederecker, F., Rathmann, W., Prehn, C., Zeller, T., Lechner, A., Adamski, J., Peters, A., and Thorand, B. (2021). Cross-sectional and prospective relationships of endogenous progestogens and estrogens with glucose metabolism in men and women: a KORA F4/FF4 study. BMJ Open Diabetes Res. Care *9*, e001951.

96. Sangster, T., Major, H., Plumb, R., Wilson, A.J., and Wilson, I.D. (2006). A pragmatic and readily implemented quality control strategy for HPLC-MS and GC-MS-based metabonomic analysis. Analyst *131*, 1075–1078.

97. Yang, H., Yang, S., Kong, J., Dong, A., and Yu, S. (2015). Obtaining information about protein secondary structures in aqueous solution using Fourier transform IR spectroscopy. Nat. Protoc. *10*, 382–396.

98. Morais, C.L.M., Lima, K.M.G., Singh, M., and Martin, F.L. (2020). Tutorial: multivariate classification for vibrational spectroscopy in biological samples. Nat. Protoc. *15*, 2143–2162.

99. Zhang, M.-L., and Zhou, Z.-H. (2014). A review on multi-label learning algorithms. IEEE Trans. Knowl. Data Eng. *26*, 1819–1837.

100. Read, J., and Hollmén, J. (2014). A deep interpretation of classifier chains. In Advances in Intelligent Data Analysis XIII (Springer International Publishing), pp. 251–262.

101. Marquardt, D.W., and Snee, R.D. (1975). Ridge regression in practice. Am. Statistician *29*, 3–20.

CellPress
OPEN ACCESS

STAR★METHODS

KEY RESOURCES TABLE

REAGENT or RESOURCE	SOURCE	IDENTIFIER
Biological samples		
Human blood plasma of KORA cohort	Helmholtz Biobank	https://helmholtz-munich.de/en/epi/cohort/kora
Pooled human blood serum	BioWest, Nuaille, France	Cat# S4200-100; Lot# S1559454200
Deposited data		
Medical examination records of KORA cohort	KORA study, Holle et al.[39]	https://helmholtz-muenchen.managed-otrs.com/external
FTIR measurements of KORA cohort	This study	https://helmholtz-muenchen.managed-otrs.com/external
Software and algorithms		
Python (version 3.8.8)	Python Software Foundation	https://python.org
NumPy (version 1.21.2)	Harris et al.[80]	https://numpy.org
Pandas (version 1.2.4)	McKinney[81]	https://pandas.pydata.org
SciPy (version 1.6.2)	Virtanen et al.[82]	https://scipy.org
Scikit-learn (version 0.24.1)	Pedregosa et al.[83]	https://scikit-learn.org
Matplotlib (version 3.5.1)	Hunter[84]	https://matplotlib.org
Other		
MIRA Analyzer (MA6) mid-infrared analyzer	Clade GmbH, Germany	https://clade.io

RESOURCE AVAILABILITY

Lead contact

Further information and requests for resources should be directed to and will be fulfilled by the lead contact, Mihaela Žigman (mihaela.zigman@mpq.mpg.de).

Materials availability

This study did not generate new unique reagents.

Data and code availability

- The data of this study are available upon request by means of a project agreement from KORA (https://helmholtz-muenchen.managed-otrs.com/external). Requests should be sent to kora.passt@helmholtz-muenchen.de and are subject to approval by the KORA Board.
- This study does not report original code. Details on the software and algorithms used in this study are listed in the key resources table and STAR Methods.
- Any additional information required to reanalyze the data reported in this study is available from the lead contact upon request.

EXPERIMENTAL MODEL AND SUBJECT DETAILS

Study design and sample collection

The KORA study is a population-based cohort in Southern Germany.[39] It served as the basis for analysis in this work. The study comprised of an age- and gender-stratified sample of participants randomly drawn from the resident registration offices within the study area. The KORA S4 baseline was conducted in 1999–2001 (n = 4261) and was followed up in 2006–2008 (n = 3080) and 2013–2014 (n = 2279) –- named KORA F4 and KORA FF4, respectively.[85] Medical examination data and blood plasma samples from the available subset of the KORA F4 (n = 3044) and FF4 samplings (n = 2140) were included in this work and were denoted by sample set #1 and sample set #2, respectively. Within these sample sets, some individuals participated in only one sample donation, while others participated in both. Across both sample sets, 2015 individuals participated in both donations, while 1154 individuals participated in only one donation. This results in a total of 5184 blood plasma donations from 3169 unique individuals. Data collection methods and standardized sample collections have been described in detail elsewhere.[39,85–87] Table S1 provides an overview of the cohort distributions. The KORA F4 and FF4 study methods were approved by the ethics committee of the Bavarian Chamber of Physicians, Munich (EC No. 06068).

Phenotype definitions, biochemical and hematological analysis

Individuals were identified as dyslipidemic based on abnormally elevated levels of non-HDL cholesterol with the commonly used cut-off point at 4.1 mmol/L.[88,89] Hypertension was defined based on a blood pressure test with a reading ≥ 140 and/or 90 mmHg or by known use of antihypertensive medication.[90] The diabetic state was determined by an oral glucose tolerance test (OGTT) or a validated self-report.[85,87] Self-reported diabetes was confirmed by contacting the responsible physicians, medical chart reviews, or by taking self-reported glucose-lowering medications.[85] For individuals with no prior diagnosis of diabetes, an OGTT test was performed and the diabetic state was determined according to the 1999 WHO diagnostic criteria.[87,91] Prediabetes included individuals with impaired fasting glucose, impaired glucose tolerance, or both. As previously applied to the KORA F4 and FF4 samples,[92] metabolic syndrome was defined according to the harmonized criteria proposed by the International Diabetes Federation (IDF) and American Heart Association/National Heart, Lung, and Blood Institute (AHA/NHLBI) in 2009.[57] Metabolic syndrome was present if ≥ 3 of the following 5 criteria were satisfied: (1) waist circumference ≥ 94 cm for males, ≥ 80 cm for females; (2) systolic blood pressure ≥ 130 mmHg or diastolic blood pressure ≥ 85 mmHg or treatment with anihypertensive medication; (3) fasting serum glucose ≥ 100 mg/dL or intake of antidiabetic medication; (4) serum HDL cholesterol < 40 mg/dL for males, < 50 mg/dL for females, or drug treatment for reduced HDL (fibrates); and (5) fasting serum triglycerides ≥ 150 mg/dL or drug treatment for elevated triglycerides (fibrates). Blood samples were collected from the cubital vein without stasis of all participants and immediately measured for the biochemical analysis using standardized protocols described elsewhere.[93–95] Blood glucose levels were determined using a hexokinase method from serum.[93,94] HbA1c was determined using a reverse-phase cation-exchange High-Pressure Liquid Chromatography (HPLC) analysis.[93] Total cholesterol, HDL cholesterol, and LDL cholesterol were determined using enzymatic, colorimetric routines (CHOD-PAP) from serum.[93,94] Triglycerides were determined using an enzymatic, colorimetric routine (GPO-PAP) from serum.[93,94] Creatinine was determined using a modified kinetic Jaffe reaction from serum. Hematological parameters (leukocytes, erythrocytes, hematocrit, platelets) were determined by impedance measurements from EDTA.[93,94] Albumin was determined using immunonephelometry from serum.[95]

Exclusion of samples

Due to missing values in the medical examination records, some samples were excluded from analysis whenever the missing values were relevant to the investigated question. The sample sizes used for each investigated question was mentioned in each corresponding figure.

METHOD DETAILS

Sample preparation and FTIR measurements

The plasma samples were stored at −80°C until analysis in this work. The samples were handled in a randomized and blinded fashion where the person performing the measurement had no access to either sample identifiers or associated medical records. In advance of the FTIR measurements, one aliquot per plasma sample was thawed and again centrifuged for 10 min at 2000 g. The supernatant was distributed into 50 μL measurement tubes and refrozen at −80°C. Thus, all FTIR measurements were performed upon two freeze-thaw cycles. Measurements were performed in the liquid phase using a commercially available automated spectrometer (MIRA Analyzer, Clade.io) with a flow-through transmission cuvette made of calcium fluoride (CaF_2) with a path length of approximately 8 μm. The spectra were acquired with a resolution of 4 cm^{-1} in a spectral range between 950 cm^{-1} and 3050 cm^{-1}. A water reference spectrum was recorded after each sample measurement to reconstruct the IR absorption spectra. To track experimental errors over extended time,[96] a measurement of quality control serum (QC, pooled human serum, BioWest, Nuaille, France) was performed after every five plasma measurements. Each measurement sequence contained up to 40 samples, including the plasma and QC samples. After each measurement sequence, the spectrometer was carefully cleaned and re-qualified according to the manufacturer's recommendations. Measurements of sample set #1 were performed over the span of 3 months. Measurements of sample set #2 were performed approximately 2.7 years prior to the measurements of sample set #1 and also spanned over 3 months. As CaF_2 is slightly soluble in water, the volume and path length of the measurement cuvette increases over time. Due to wearing off, different transmission cuvettes were used between measurements of the first and second sample sets. The spectra of the QCs were used to evaluate the measurement error. In our previous study, involving the same experimental procedures, we found that the measurement error was small when compared to the inter-person biological variability of IR spectra of serum.[78]

Measurement preprocessing

Preprocessing of the IR spectra was performed as in our previous study.[21] If the liquid sample contains less water than the reference (pure water), negative absorption occurs. This was corrected by a previously described approach where a scaled water absorption spectrum was added to each sample spectrum using a coefficient optimized such that the first derivative of the signal between 2000 and 2300 cm^{-1} is minimal.[21,78,97] Subsequently, all spectra were truncated to 1000–3000 cm^{-1} and the region between 1800 and 2800 cm^{-1} was removed since, in plasma, it observes no significant and biologically relevant absorbance. Each measured spectrum was then treated as a vector and normalized using the L2 norm, unless otherwise mentioned in the figure legend. The normalization helps to correct for measurement noise and spectral differences that can arise from changes in the total molecular concentration in

the samples (e.g., owing to variations in sample collection, storage/handling, and processing) as described in previous work.[78,98] Figure S2 depicts the effect normalization had on the comparability between different groups of samples.

QUANTIFICATION AND STATISTICAL ANALYSIS

Classification analysis

An L2-regularized logistic regression algorithm was used for the classification analysis. To enable multilabel predictions, where an input can be assigned to multiple labels (i.e., phenotypes), a chaining method was employed.[42,99] The strategy consisted of fitting one logistic regression classifier per modeled label. The architecture and label order of the classification chain was depicted in Figure 2B. When training, each classifier was fit on a training set of IR spectra, in addition to the true labels of the investigated phenotypes. The first classifier in the chain had no additional information other than the input IR spectra and the true labels relevant to its prediction (dyslipidemia). Subsequent classifiers had additional access to the true labels from all preceding phenotypes when training. When making predictions, only the input IR spectra were used and the predictions of each preceding classifier got passed on to all other classifiers coming within the chain since all the true labels are hidden from the classifier chain. Naturally, the order of the classification had an effect on the predictive performance of the multilabel classification. To investigate its effects and select a model with the optimal classification order, an exhaustive grid search was carried out on all possible permutations of ordering the 5 labels to examine the predictive performance of each multilabel classification variant. The grid search was only carried out on measurements from sample set #1 where each label order was tested in a 10-fold cross-validation. A large number of label arrangements were found to lead to similar predictive efficacies (Table S4). It was reassuring to observe that the top performing models followed analytically comparable label arrangements – e.g., the classifier detecting whether an individual was healthy remained toward the end of the chain. From the total of 120 possible label arrangements, the order which maximized the exact match ratio was selected since this investigation on sample set #1 showed that consistent results would be achievable using different arrangements. The commonly used metrics of exact match ratio, Hamming score, and Hamming loss were used as measures of overall multilabel performance (further explained below).[43–45,100] Based on the probabilities given by the classifiers, a threshold of 0.5 was selected to identify each positive class prediction from a negative class prediction. Furthermore, the ROC curve was examined for each logistic regression within the chain to observe how each binary classification performed across all possible prediction thresholds. Classifier validation is described in the following section.

Validation of multilabel and binary classifications

All reported metrics of classification performance were validated on unseen test samples (Figure S1). Relevant to the results depicted in Figures 2C, 2D, 3C and 3D, three types of validations were performed: (1) a 10-fold cross-validation on sample set #1; (2) another 10-fold cross-validation on sample set #2; and (3) a sample set-independent validation. For the sample set-independent validation, our aim was to investigate how the classifications performed when tested on samples measured years apart and on entirely different individuals from the training samples. Since a large portion of individuals overlapped between the two sample sets utilized in our study, we could not directly train on sample set #1 and test on sample set #2. Instead, we held-out approximately 10% of the individuals that overlapped between the two sample sets to be used for classifier testing. We then trained the classifier on sample set #1, using individuals not included in the held-out set. The trained classifier was then tested on the held-out portion of individuals from sample set #2. This procedure was then repeated 10 times, holding-out a different 10% of test individuals in each iteration. This allowed us to retain a large number of samples for both classifier training and testing while not violating the goal of training and testing on fully different individuals from the two samples sets. Classification performance metrics (the exact match ratio, Hamming score, Hamming loss, and ROC curves) were averaged across all test splits of the data and reported along with their standard deviations.

Multilabel classification metrics

In multilabel classifications, unlike binary or multiclass classifications, one input may be simultaneously associated with multiple outputs/labels. Interpreting the efficacy of such predictions is more complex as predictions may be entirely correct, partially correct to varying degrees, or entirely incorrect. Therefore, several metrics that judge the prediction efficacy differently should be used to judge the multilabel prediction. In this study, three established metrics were used: Exact match ratio,[42,45] Hamming score,[43–45] and Hamming loss.[42,43,45] Descriptions of each metric are provided below. An illustrative example of interpreting each metric is provided in Table S5.

The exact match ratio is a strict metric that treats partially correct predictions as entirely incorrect. Given a sample $\mathbf{x}_i$, the prediction is scored with a 1 only if the prediction $\widehat{\mathbf{y}}_i$ exactly matches the true label vector $\mathbf{y}_i$ associated with the sample. Otherwise, the prediction is scored with a 0. This evaluation was performed for every sample and averaged to provide the proportion of exactly matched predictions. Its values range from 0 to 1, where higher values indicate better prediction efficacy. The metric is expressed as the following where I is the indicator function:

$$\text{Exact Match Ratio} = \frac{1}{n_{samples}} \sum_{i=1}^{n_{samples}} \mathbb{I}(\mathbf{y}_i = \widehat{\mathbf{y}}_i)$$

The Hamming score, also known as the Jaccard similarity coefficient, considers partially correct predictions to different degrees. Given a sample, it is defined as the proportion of predicted positive labels matching the true labels to the total number of possible positive (predicted and actual) labels for that sample. A predicted positive label here implies the existence of a certain label/condition (e.g., a phenotype is observed for a given sample). The metric does not reward a model correctly predicting that a label does not apply to a given sample (true negative), while it penalizes false positive predictions. The metric is therefore more strict than simply calculating the fraction of predicted labels that match the true labels. Its values range from 0 to 1, where higher values indicate better prediction efficacy, and is expressed as the following:

$$\text{Hamming Score} = \frac{1}{n_{samples}} \sum_{i=1}^{n_{samples}} \frac{|\mathbf{y}_i \cap \widehat{\mathbf{y}}_i|}{|\mathbf{y}_i \cup \widehat{\mathbf{y}}_i|}$$

The Hamming loss is defined as the fraction of incorrectly predicted labels across all instances and labels. It considers each class individually and counts the number of times the model's predictions differ from the actual labels. Its values range from 0 to 1, where lower values indicate better prediction efficacy (i.e., lower label misclassification rate). To keep a consistent interpretation between the three metrics, where higher values indicate better predictive efficacy, we calculated 1 - Hamming loss as follows:

$$1 - \text{Hamming loss} = 1 - \frac{1}{n_{samples} * n_{labels}} \sum_{i=1}^{n_{samples}} \sum_{j=1}^{n_{labels}} \mathbb{I}\left(y_{i,j} \neq \widehat{y}_{i,j}\right)$$

In the multilabel analysis applied in our study, we considered five labels. These labels can occur in any of 12 different clinically feasible combinations, each with varying prevalence (Figure 2A). We performed a stochastic procedure to establish a benchmark for comparing the prediction value in relation to random chance. For each of the 12 unique label combinations, we randomly sampled 1000000 5-dimensional vectors that describe the labels of the samples observed in our dataset. These sampled label vectors thus preserve the clinically feasible label combinations that may occur and the prevalence of each label combination in the population (i.e., the sampling preserves the distribution of the labels). The same sampling procedure was then repeated but with a shuffling procedure, thus breaking the correspondence between the two sets of 100000 5-dimensional vectors. We then computed the three metrics described above on the two sets of 100000 5-dimensional vectors. This procedure resulted in a stochastic estimate of the three evaluation metrics for a classifier making clinically feasible predictions that follow the true label distributions but was unable to learn when to make which prediction. These estimates served as a benchmark to assess the multilabel model's predictive efficacy and were depicted by dashed lines in Figure 2C.

Regression analysis

An L2-regularized linear regression algorithm (ridge regression) was used for the regression analysis. IR spectra from both sample sets were combined together and used as the model inputs. Predictive performance was estimated by the root mean squared error (RMSE) and coefficient of determination (R^2) in 10-fold cross-validations. The metrics of evaluations were calculated on the test sets, averaged across the 10 cross-validation splits, and reported along with their standard deviation. The predicted values from the test splits were further plotted and compared to the clinically determined ones (Figure 4B).

Feature selection analysis

No explicit feature selection algorithm was applied prior to the classification and regression analysis. Instead, our approach relied on the relatively large sample sizes and the established ability of multivariate predictive analysis to discern optimal weights through L2 (ridge) regularization,[101] alongside comprehensive testing of predictive efficacy on unseen test samples.

Analysis software

Data analysis was performed using Python (version 3.8.8). The open-source Scikit-learn package (version 0.24.1) was used for its implementations of the logistic regression and linear regression algorithms.[83]

Cell Reports Medicine, Volume *5*

Supplemental information

Plasma infrared fingerprinting with machine learning enables single-measurement multi-phenotype health screening

Tarek Eissa, Cristina Leonardo, Kosmas V. Kepesidis, Frank Fleischmann, Birgit Linkohr, Daniel Meyer, Viola Zoka, Marinus Huber, Liudmila Voronina, Lothar Richter, Annette Peters, and Mihaela Žigman

	**Sample set #1** (3044 samples)	**Sample set #2** (2140 samples)	**Combined** (5184 samples)
**Male proportion, %**	48.23	48.79	48.46
**Age, mean (± SD),** ***years***	56.19 (±13.24)	60.19 (±12.30)	57.84 (±13.01)
**Body mass index, mean (± SD),** ***kg/m***^{2}	27.62 (±4.81)	27.77 (±4.92)	27.68 (±4.85)
**Total cholesterol, mean (± SD),** ***mmol/l***	5.58 (±1.02)	5.58 (±1.01)	5.58 (±1.02)
**LDL cholesterol, mean (± SD),** ***mmol/l***	3.51 (±0.90)	3.48 (±0.90)	3.50 (±0.90)
**HDL cholesterol, mean (± SD),** ***mmol/l***	1.45 (±0.37)	1.69 (±0.48)	1.55 (±0.44)
**Triglycerides, mean (± SD),** ***mmol/l***	1.42 (±0.97)	1.38 (±0.81)	1.40 (±0.90)
**Hematocrit, mean (± SD),** ***l/l***	0.41 (±0.03)	0.41 (±0.03)	0.41 (±0.03)
**Erythrocytes, mean (± SD),** ***/pl***	4.53 (±0.42)	4.60 (±0.43)	4.56 (±0.42)
**Leukocytes, mean (± SD),** ***/nl***	5.98 (±1.88)	5.98 (±1.66)	5.98 (±1.79)
**Platelets, mean (± SD),** ***/nl***	253.99 (±62.29)	229.52 (±55.84)	243.89 (±60.91)
**HbA1c, mean (± SD),** ***mmol/mol***	37.21 (±6.80)	37.05 (±6.99)	37.14 (±6.88)
**Fasting glucose, mean (± SD),** ***mmol/l***	5.46 (±1.08)	5.69 (±1.19)	5.55 (±1.13)
**Albumin, mean (± SD),** ***g/l***	44.57 (±3.38)	42.54 (±3.17)	43.73 (±3.44)
**Creatinine, mean (± SD),** μ***mol/l***	80.45 (±21.58)	80.57 (±20.67)	80.50 (±21.21)

Supplementary Table S1. Description of study cohort, related to STAR Methods. Two time-separated samplings of a longitudinal population-based cohort were considered.

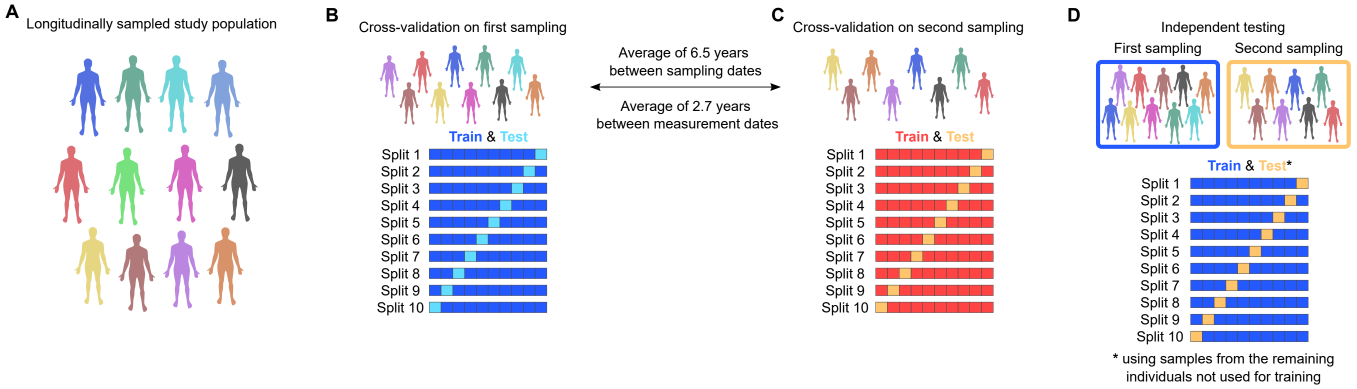

Supplementary Figure S1. Model validation procedure, related to STAR Methods. (**A**) Studied population consisting of naturally occurring heterogeneity between individuals that was sampled at two time points, thus longitudinal. (**B**) On the first sample set of the two, 10-fold cross-validation was performed to obtain metrics of model performance in 10 train and test splits of the samples. Each split was trained on approximately 90% of the samples and tested on approximately 10% of the remaining samples. (**C**) On the second sample set, the same cross-validation procedure was performed – independently of the first. (**D**) In 10 splits, model training was performed on 90% of the first sample set, leaving out a different set of individuals in each iteration. Model testing was then performed on the individuals from the second sample set who were not included in the training data from the first sample set for each data split. The model was thus tested on a dataset of independent samples and individuals.

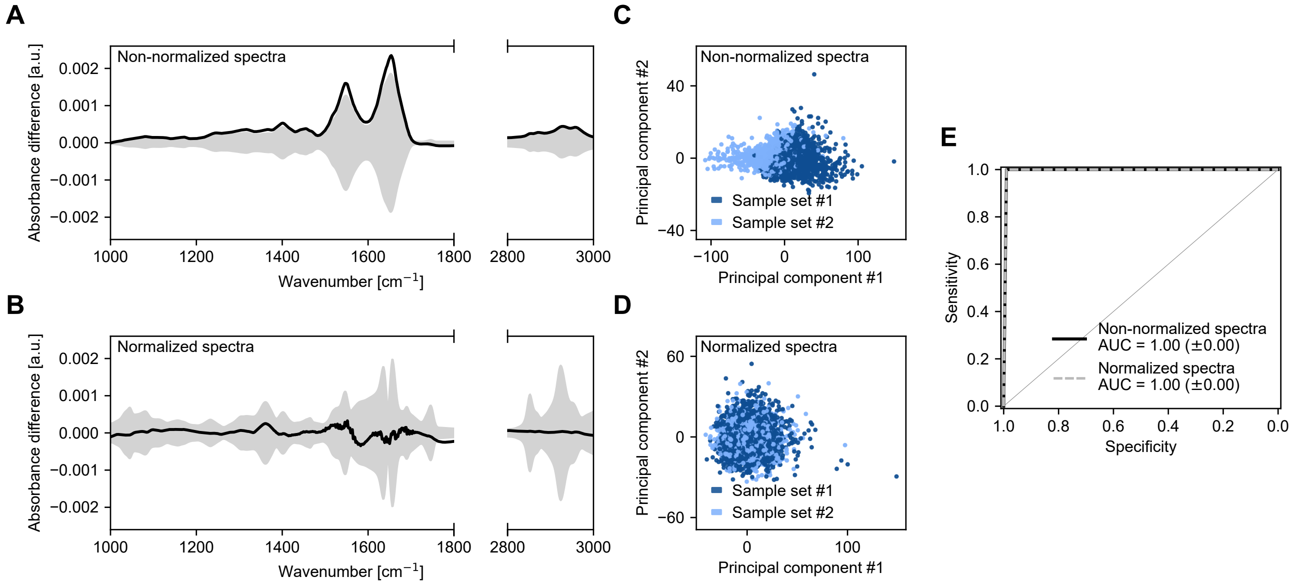

Supplementary Figure S2. Effects of variations in sample and measurement conditions on IR molecular fingerprints, related to STAR Methods. (**A**–**B**) Difference between the mean measurement of the first sample set and the mean measurement of the second on (**A**) non-normalized spectra and (**B**) normalized spectra. Gray-shaded areas depict the standard deviation of the IR fingerprints. (**C**–**D**) Principal component analysis was applied on the combined set of spectra from the two sample sets on (**C**) non-normalized spectra and (**D**) normalized spectra, color-coding the measurements from each sample set. (**E**) Binary classifications were carried out to determine to which sample set a measurement belonged to. Both sample sets were combined into one dataset, and the classifier was trained to assign each measurement into one sample set to determine whether there exists differences between the IR spectra of the sample sets. Two ROC curves of the classifications are depicted – one when applied to normalized spectra and one when applied to non-normalized spectra. The ROC curves are depicted on test samples as determined via cross-validation. Both classifiers were capable of perfectly determining to which sample set each measurement belonged to – revealing that measurement variations existed between the sample sets (even on normalized spectra), despite the overlapping cloud appearance depicted in (**D**).

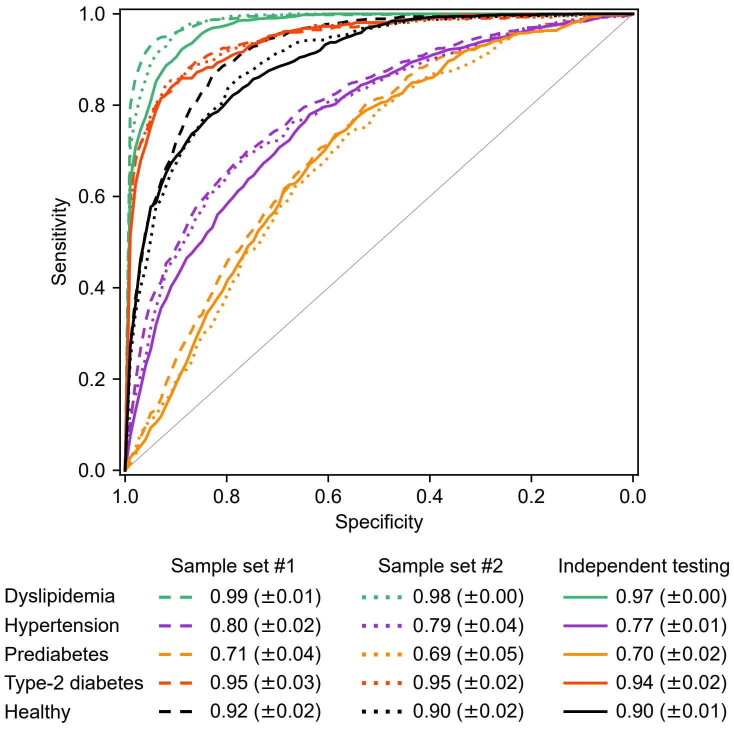

Supplementary Figure S3. Receiver operating characteristic (ROC) curves for binary classifications modeling each listed outcome against all other outcomes (one-versus-rest) without the classifier chaining, related to Figure 2. For each classification, the mean cross-validated ROC curve of the test sets is depicted and the area under the curve is listed below, along with its standard deviation. Model validation was carried out as described in the main text. Compared to the ROC curves depicted in Figure 2**D** in the main text, the binary classification analysis here was performed with independent binary classifiers that do not utilize the chain mechanism.

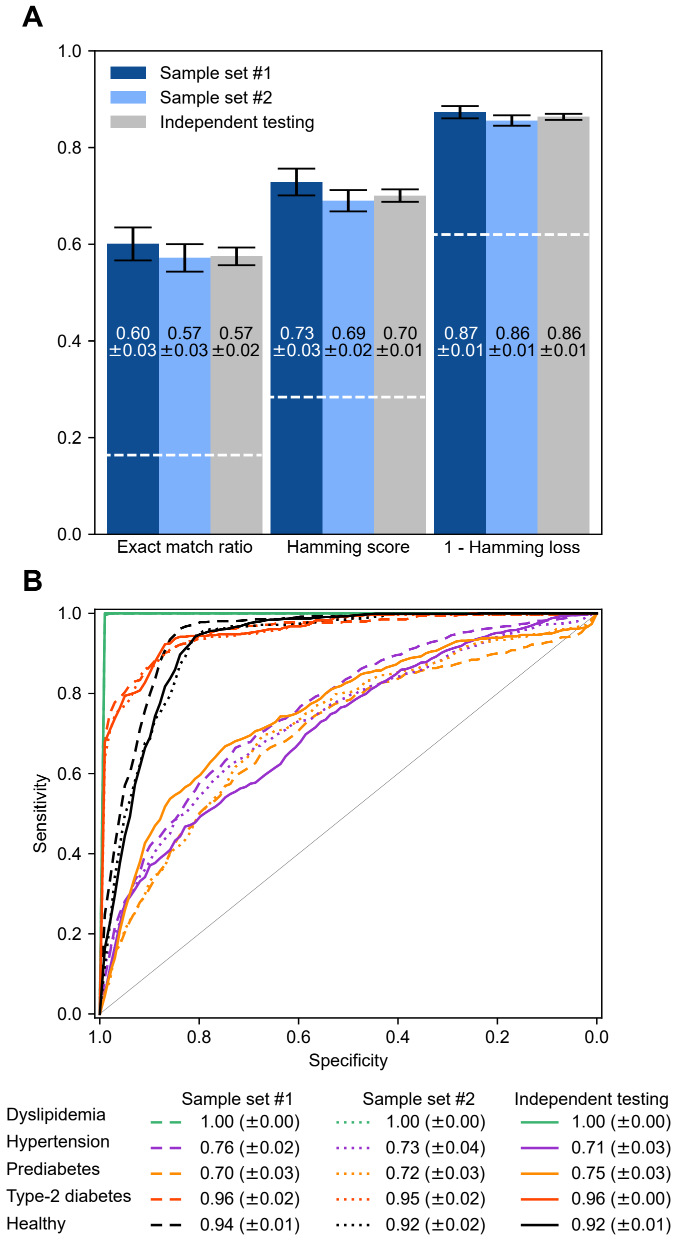

Supplementary Figure S4. Capacity of clinical analytes to predict common phenotypes, related to Figure 2. Twelve clinical analytes (total cholesterol, LDL cholesterol, HDL cholesterol, triglycerides, hematocrit, erythrocytes, leukocytes, platelets, HbA1c, fasting glucose, albumin, and creatinine) were used to compare the performance of simultaneously detecting the phenotypes of interest with the performance of using the IR molecular fingerprints as exclusive predictors (shown in Figure 2 in the main text). (**A**–**B**) Test predictive performance of a model using the clinical analytes as the exclusive predictors. Model validation was carried out as described in the main text.

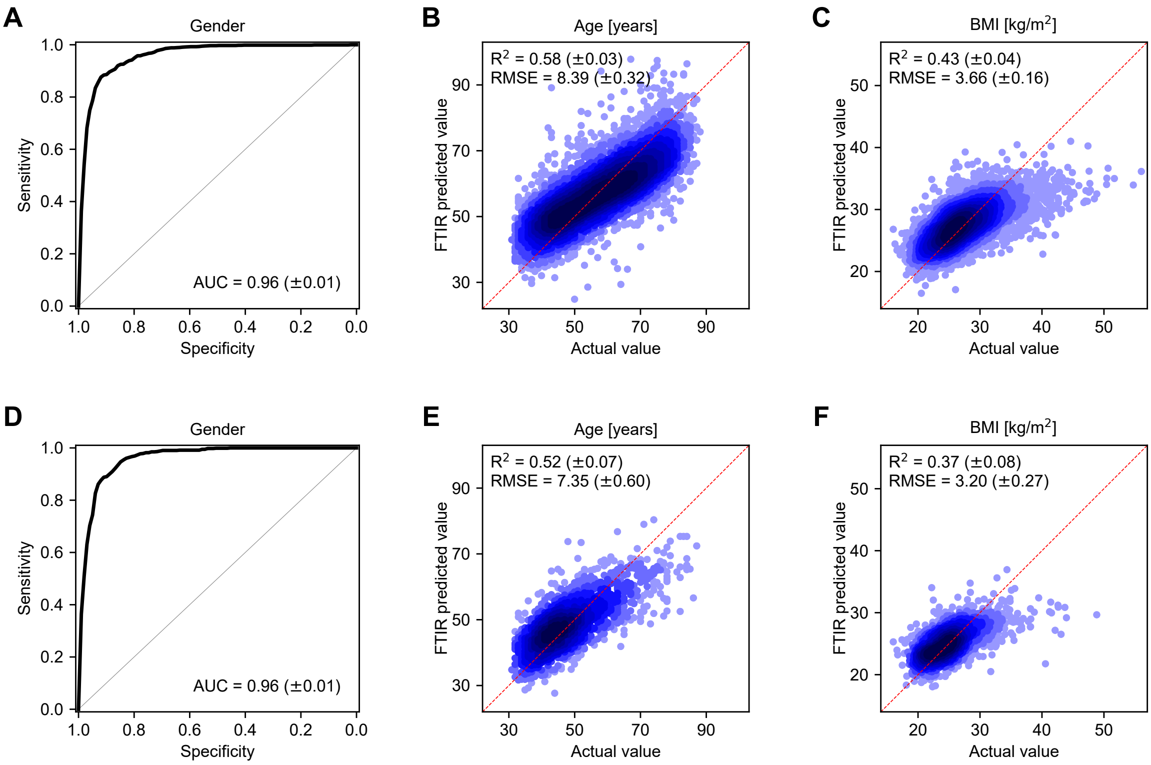

Supplementary Figure S5. Prediction of anthropometric parameters using IR molecular fingerprints, related to Figure 2. (**A**) Test ROC curves for distinguishing males from females. Classification performance was estimated by repeatedly training on 90% of the first sample set and testing on the second sample set in 10 iterations, ensuring no overlap between the longitudinally sampled individuals. (**B**–**C**) Prediction error of regression models trained to estimate (**B**) age and (**C**) body mass index (BMI) of the sampled individuals, combining samples from both sets. The predicted values follow from the test sets of models fit on training sets in a 10-fold cross-validation where each point represents a measurement. (**D**–**F**) Same procedure, but applied on the healthy subset of the population to reduce the contribution of the health phenotypes on the anthropometric parameter predictions.

		Sample set #1		Sample set #2	
		**Controls**	**Cases**	**Controls**	**Cases**
**Dyslipidemia**	***n***	1298	1298	856	856
	**Male proportion**, %	50.8	50.4	51.6	51.8
	**Age, mean (± SD),** ***years***	56.3 (±13.3)	57.8 (±12.1)	60.7 (±11.5)	60.6 (±11.3)
	**Body mass index, mean (± SD),** ***kg/m***^{2}	27.5 (±4.8)	28.2 (±4.5)	28.4 (±4.8)	28.6 (±4.7)
**Hypertension**	***n***	757	757	551	551
	**Male proportion**, %	50.5	50.5	49.7	49.7
	**Age, mean (± SD),** ***years***	59.4 (±11.3)	61.1 (±11.4)	63.1 (±10.6)	64.6 (±10.4)
	**Body mass index, mean (± SD),** ***kg/m***^{2}	28.0 (±4.1)	28.5 (±4.2)	27.9 (±4.3)	28.5 (±4.4)
**Prediabetes**	***n***	492	492	375	375
	**Male proportion**, %	52.8	52.8	57.1	57.1
	**Age, mean (± SD),** ***years***	63.1 (±11.0)	63.2 (±11.0)	65.1 (±11.1)	65.2 (±11.0)
	**Body mass index, mean (± SD),** ***kg/m***^{2}	29.7 (±4.7)	29.7 (±4.8)	29.6 (±4.8)	29.6 (±4.8)
**Type-2 diabetes**	***n***	335	335	295	295
	**Male proportion**, %	59.4	59.4	60.0	60.0
	**Age, mean (± SD),** ***years***	66.9 (±9.3)	67.1 (±9.3)	69.2 (±9.9)	69.4 (±10.1)
	**Body mass index, mean (± SD),** ***kg/m***^{2}	31.1 (±5.0)	31.2 (±5.1)	30.9 (±5.2)	31.1 (±5.3)
**Healthy**	***n***	715	715	526	526
	**Male proportion**, %	45.5	45.5	43.9	43.9
	**Age, mean (± SD),** ***years***	51.0 (±11.2)	49.3 (±11.4)	56.1 (±10.5)	54.5 (±10.8)
	**Body mass index, mean (± SD),** ***kg/m***^{2}	26.2 (±3.9)	25.8 (±4.0)	26.5 (±3.9)	26.0 (±4.1)

Supplementary Table S2. Description of cohorts used in case-control matched classifications, related to Figure 2 and Figure S6. Matching was performed based on gender, age, and body mass index of the sampled individuals in a pair-wise fashion.

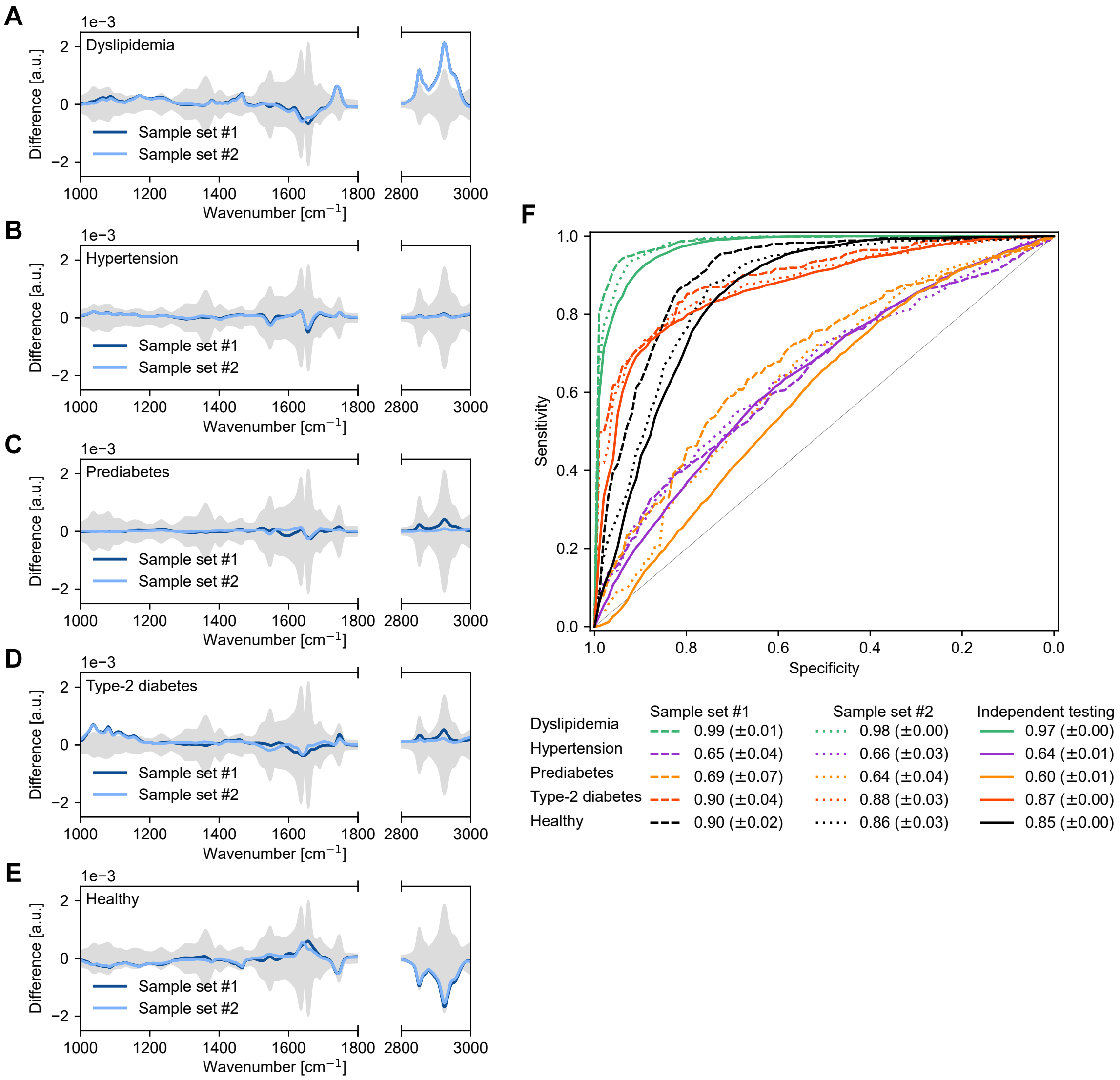

Supplementary Figure S6. Phenotype detection in gender, age, and body mass index matched cohorts using IR molecular fingerprints, related to Figure 2 and Table S2. (**A**–**E**) Difference between the mean spectral measurement of cases and controls for each condition in the first sample set (dark blue) and second sample set (light blue). The "healthy" category includes individuals having none of the other listed conditions. (**F**) ROC curves for the binary classifications detecting each phenotype. The mean cross-validated ROC curve of the test sets is depicted and the area under the curve is listed, along with its standard deviation, for each binary classification.

Sample set #1 (baseline)						
**No. of risk factors**	**Sample count**	**Visceral obesity**	**Hypertriglyceridemia**	**Low HDL cholesterol**	**Hypertension**	**Hyperglycemia**
**0**	547	0 (0.0%)	0 (0.0%)	0 (0.0%)	0 (0.0%)	0 (0.0%)
**1**	709	473 (66.7%)	30 (4.2%)	40 (5.6%)	105 (14.8%)	61 (8.6%)
**2**	659	555 (84.2%)	146 (22.2%)	169 (25.6%)	264 (40.1%)	184 (27.9%)
**3**	548	504 (92.0%)	318 (58.0%)	238 (43.4%)	296 (54.0%)	288 (52.6%)
**4**	365	347 (95.1%)	333 (91.2%)	278 (76.2%)	214 (58.6%)	288 (78.9%)
**5**	163	163 (100.0%)	163 (100.0%)	163 (100.0%)	163 (100.0%)	163 (100.0%)
**Sample set #2 (follow-up)**						
**No. of risk factors**	**Sample count**	**Visceral obesity**	**Hypertriglyceridemia**	**Low HDL cholesterol**	**Hypertension**	**Hyperglycemia**
**0**	291	0 (0.0%)	0 (0.0%)	0 (0.0%)	0 (0.0%)	0 (0.0%)
**1**	562	434 (77.2%)	14 (2.5%)	7 (1.2%)	39 (6.9%)	68 (12.1%)
**2**	488	432 (88.5%)	107 (21.9%)	50 (10.2%)	130 (26.6%)	257 (52.7%)
**3**	375	361 (96.3%)	215 (57.3%)	135 (36.0%)	172 (45.9%)	242 (64.5%)
**4**	290	278 (95.9%)	281 (96.9%)	216 (74.5%)	121 (41.7%)	264 (91.0%)
**5**	97	97 (100.0%)	97 (100.0%)	97 (100.0%)	97 (100.0%)	97 (100.0%)

Supplementary Table S3. Breakdown of metabolic risk factors for pre-MetS and MetS investigations, related to Figure 3. Each row represents a group of individuals with varying numbers of concurrent risk factors. The sample counts and percentages are listed according to the distribution of risk factors observed in each group. Distributions of the first and second sample sets are listed separately. Samples with unknown values for any of the risk factors were excluded.

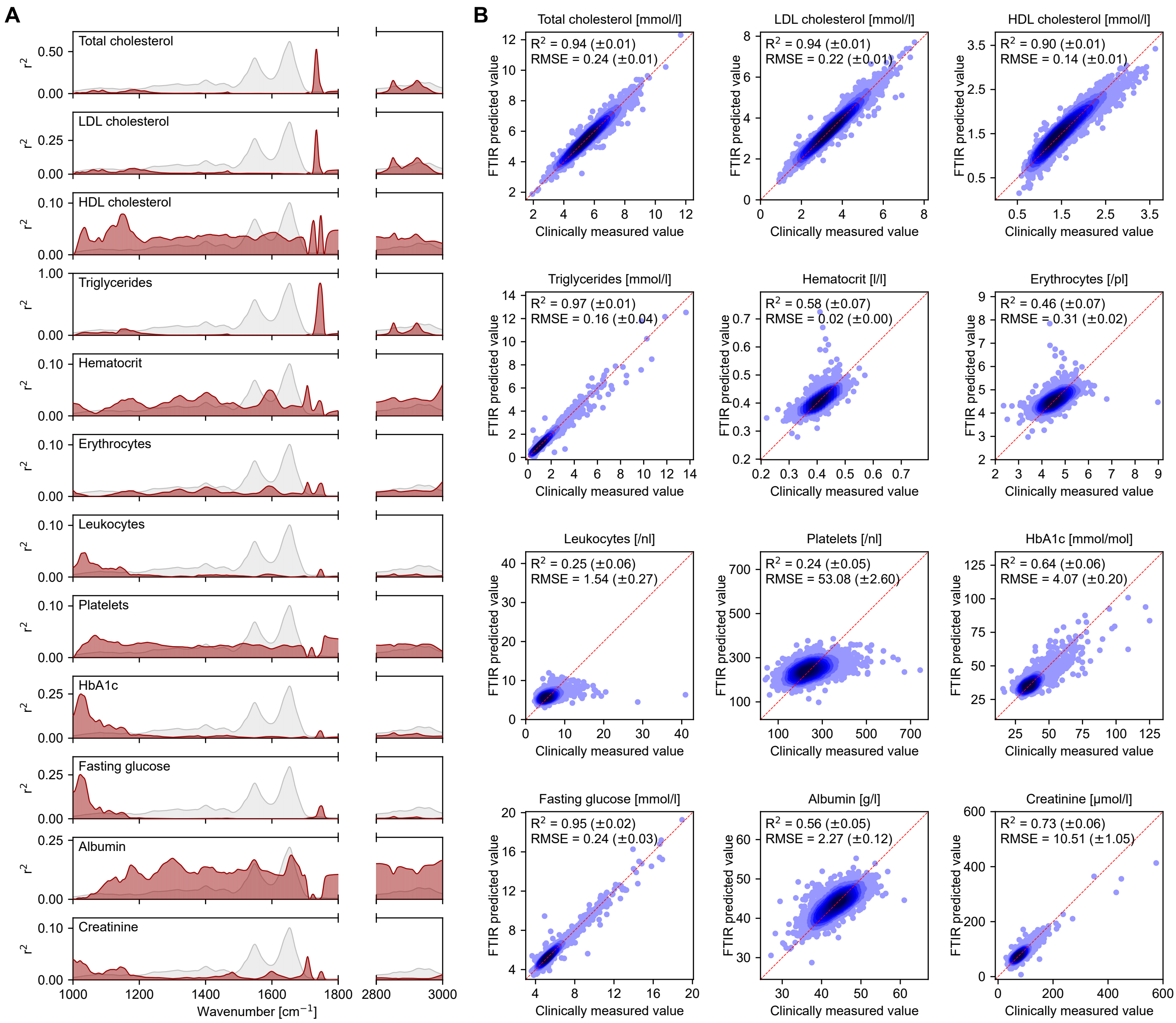

Supplementary Figure S7. Classical clinical analytes reflected in non-normalized IR molecular fingerprints, related to Figure 4. (**A**) Pearson correlation coefficient (red curves) between the concentrations of each clinical analyte and the absorbance at each wavenumber between 1000 and 3000 cm^{-1} for non-normalized spectra. The mean absorbance spectrum of all measured IR spectra (n = 5184) is overlaid in gray on each panel as a visual reference for the shape of the spectrum. (**B**) Performance of quantitatively predicting clinical analytes using the non-normalized IR spectra of the measured population. Regression algorithms were trained for each parameter to capture the relations between the clinically-measured values and the multivariate spectral features. The predicted values follow from the test sets of 10-fold cross-validations. Each point represents a measurement. The mean coefficient of determination (R^2) and the root mean squared error (RMSE) are listed for each parameter, along with their standard deviations across the test splits. The diagonal (dashed red line) is a visual reference for a perfect fit.

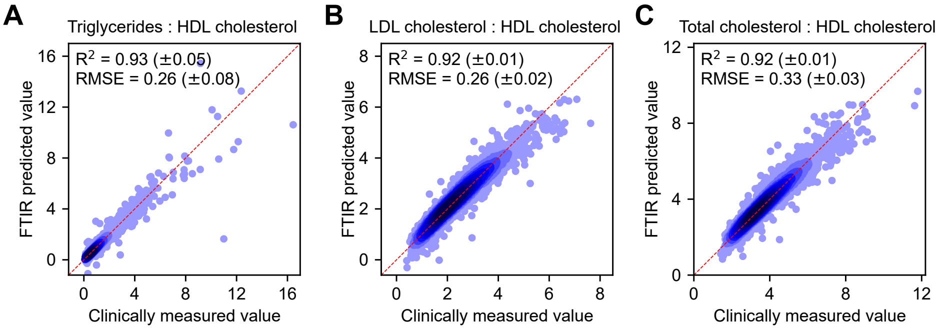

Supplementary Figure S8. Performance of quantitatively predicting cholesterol ratios using IR molecular fingerprints, related to Figure 4. Ratios were calculated by dividing the clinically measured mmol/l concentrations of triglycerides by HDL cholesterol (**A**), LDL cholesterol by HDL cholesterol (**B**), and total cholesterol by HDL cholesterol (**C**). Regression algorithms were trained for each ratio to capture the relations between the calculated values and the multivariate spectral features. Predicted values follow from test sets of models fit on training sets in a 10-fold cross-validation. Each point represents a measurement. Model performance was measured by the mean coefficient of determination (R^2) and the root mean squared error (RMSE) and is listed for each parameter, along the standard deviation, across the test sets of the cross-validation. The diagonal (dashed red line) represents a visual reference for a perfect fit.

Supplementary Table S4. Predictive performance of multilabel classification using different label arrangements for optimal model selection, related to STAR Methods. To select an optimal classification order, several permutations were tested to observe their influence on the overall predictive efficacy. For each listed order, model performance was estimated by cross-validating on only sample set #1 to avoid introducing model selection bias by leaking information from sample set #2 (which was used for the independent testing). The mean and standard deviation of the calculated exact match ratio, Hamming score, and Hamming loss is listed for each of the possible 120 ordering of the 5 binary classifiers in the chain. The last entry in the table lists the performance of a non-chained model where each of the 5 phenotypes was detected with an independent binary classifier. Abbreviations: T2D – type-2 diabetes; PD – prediabetes.

#	Label order	Exact match ratio	Hamming score	1 - Hamming loss
**1**	Dyslipidemia → Hypertension → T2D → PD → Healthy	0.58 (±0.03)	0.71 (±0.03)	0.86 (±0.01)
**2**	Hypertension → T2D → Dyslipidemia → PD → Healthy	0.58 (±0.03)	0.71 (±0.03)	0.86 (±0.01)
**3**	Hypertension → Dyslipidemia → T2D → PD → Healthy	0.57 (±0.03)	0.71 (±0.03)	0.86 (±0.01)
**4**	PD → Hypertension → T2D → Dyslipidemia → Healthy	0.57 (±0.03)	0.70 (±0.03)	0.86 (±0.01)
**5**	Dyslipidemia → Hypertension → PD → T2D → Healthy	0.57 (±0.03)	0.70 (±0.03)	0.86 (±0.01)
**6**	T2D → Hypertension → Dyslipidemia → PD → Healthy	0.57 (±0.03)	0.71 (±0.03)	0.86 (±0.01)
**7**	PD → Hypertension → Dyslipidemia → T2D → Healthy	0.57 (±0.04)	0.70 (±0.03)	0.86 (±0.01)
**8**	Dyslipidemia → PD → Hypertension → T2D → Healthy	0.57 (±0.04)	0.70 (±0.03)	0.86 (±0.01)
**9**	Hypertension → PD → T2D → Dyslipidemia → Healthy	0.57 (±0.03)	0.70 (±0.03)	0.86 (±0.01)
**10**	Hypertension → PD → Dyslipidemia → T2D → Healthy	0.57 (±0.03)	0.70 (±0.03)	0.86 (±0.01)
**11**	Hypertension → Dyslipidemia → PD → T2D → Healthy	0.57 (±0.03)	0.70 (±0.03)	0.86 (±0.01)
**12**	PD → Dyslipidemia → Hypertension → T2D → Healthy	0.57 (±0.04)	0.70 (±0.03)	0.86 (±0.01)
**13**	Dyslipidemia → Hypertension → Healthy → PD → T2D	0.57 (±0.03)	0.70 (±0.03)	0.86 (±0.01)
**14**	PD → T2D → Hypertension → Dyslipidemia → Healthy	0.57 (±0.04)	0.70 (±0.03)	0.86 (±0.01)
**15**	Dyslipidemia → T2D → Hypertension → PD → Healthy	0.57 (±0.03)	0.70 (±0.03)	0.86 (±0.01)
**16**	T2D → Dyslipidemia → Hypertension → PD → Healthy	0.57 (±0.03)	0.70 (±0.03)	0.86 (±0.01)
**17**	Hypertension → T2D → PD → Dyslipidemia → Healthy	0.57 (±0.03)	0.70 (±0.03)	0.86 (±0.01)
**18**	Dyslipidemia → Hypertension → PD → Healthy → T2D	0.57 (±0.03)	0.70 (±0.03)	0.86 (±0.01)
**19**	Hypertension → Dyslipidemia → Healthy → PD → T2D	0.57 (±0.03)	0.70 (±0.03)	0.86 (±0.01)
**20**	PD → Hypertension → Dyslipidemia → Healthy → T2D	0.57 (±0.03)	0.70 (±0.03)	0.86 (±0.01)
**21**	T2D → Hypertension → PD → Dyslipidemia → Healthy	0.57 (±0.03)	0.70 (±0.03)	0.86 (±0.01)
**22**	Dyslipidemia → PD → Hypertension → Healthy → T2D	0.57 (±0.04)	0.70 (±0.03)	0.86 (±0.01)
**23**	Dyslipidemia → PD → T2D → Hypertension → Healthy	0.57 (±0.04)	0.70 (±0.03)	0.86 (±0.01)
**24**	PD → T2D → Dyslipidemia → Hypertension → Healthy	0.57 (±0.04)	0.70 (±0.03)	0.86 (±0.01)
**25**	Hypertension → PD → Dyslipidemia → Healthy → T2D	0.57 (±0.03)	0.70 (±0.03)	0.86 (±0.01)
**26**	Hypertension → Dyslipidemia → PD → Healthy → T2D	0.57 (±0.03)	0.70 (±0.03)	0.86 (±0.01)
**27**	PD → Dyslipidemia → Hypertension → Healthy → T2D	0.57 (±0.04)	0.70 (±0.03)	0.86 (±0.01)
**28**	PD → Dyslipidemia → T2D → Hypertension → Healthy	0.57 (±0.04)	0.70 (±0.03)	0.86 (±0.01)
**29**	Dyslipidemia → Hypertension → T2D → Healthy → PD	0.57 (±0.03)	0.70 (±0.03)	0.86 (±0.01)
**30**	Dyslipidemia → Hypertension → Healthy → T2D → PD	0.57 (±0.03)	0.70 (±0.03)	0.86 (±0.01)
**31**	PD → Hypertension → T2D → Healthy → Dyslipidemia	0.57 (±0.03)	0.70 (±0.03)	0.86 (±0.01)
**32**	T2D → PD → Hypertension → Dyslipidemia → Healthy	0.57 (±0.04)	0.70 (±0.03)	0.86 (±0.01)
**33**	Hypertension → T2D → Dyslipidemia → Healthy → PD	0.57 (±0.03)	0.70 (±0.03)	0.86 (±0.01)
**34**	Hypertension → PD → T2D → Healthy → Dyslipidemia	0.57 (±0.03)	0.70 (±0.03)	0.86 (±0.01)
**35**	Hypertension → Dyslipidemia → T2D → Healthy → PD	0.57 (±0.03)	0.70 (±0.03)	0.86 (±0.01)
**36**	PD → T2D → Hypertension → Healthy → Dyslipidemia	0.57 (±0.04)	0.70 (±0.03)	0.86 (±0.01)
**37**	Dyslipidemia → T2D → PD → Hypertension → Healthy	0.57 (±0.04)	0.70 (±0.03)	0.86 (±0.01)
**38**	T2D → Dyslipidemia → PD → Hypertension → Healthy	0.57 (±0.04)	0.70 (±0.03)	0.86 (±0.01)
**39**	Hypertension → Dyslipidemia → Healthy → T2D → PD	0.57 (±0.03)	0.70 (±0.03)	0.86 (±0.01)
**40**	T2D → Hypertension → Dyslipidemia → Healthy → PD	0.57 (±0.03)	0.70 (±0.03)	0.86 (±0.01)
**41**	Hypertension → T2D → PD → Healthy → Dyslipidemia	0.57 (±0.03)	0.70 (±0.03)	0.86 (±0.01)
**42**	T2D → PD → Dyslipidemia → Hypertension → Healthy	0.57 (±0.04)	0.70 (±0.03)	0.86 (±0.01)
**43**	T2D → Hypertension → PD → Healthy → Dyslipidemia	0.57 (±0.03)	0.70 (±0.03)	0.86 (±0.01)
**44**	Dyslipidemia → T2D → Hypertension → Healthy → PD	0.57 (±0.04)	0.70 (±0.03)	0.86 (±0.01)
**45**	T2D → Dyslipidemia → Hypertension → Healthy → PD	0.57 (±0.03)	0.70 (±0.03)	0.86 (±0.01)
**46**	T2D → PD → Hypertension → Healthy → Dyslipidemia	0.57 (±0.04)	0.70 (±0.03)	0.86 (±0.01)
**47**	PD → Hypertension → Healthy → Dyslipidemia → T2D	0.56 (±0.04)	0.69 (±0.03)	0.85 (±0.01)
**48**	Dyslipidemia → PD → T2D → Healthy → Hypertension	0.56 (±0.04)	0.70 (±0.03)	0.86 (±0.01)
**49**	PD → Hypertension → Healthy → T2D → Dyslipidemia	0.56 (±0.03)	0.69 (±0.03)	0.85 (±0.01)
**50**	PD → T2D → Dyslipidemia → Healthy → Hypertension	0.56 (±0.04)	0.70 (±0.03)	0.86 (±0.01)
**51**	Hypertension → PD → Healthy → Dyslipidemia → T2D	0.56 (±0.03)	0.69 (±0.03)	0.85 (±0.01)
**52**	PD → Dyslipidemia → T2D → Healthy → Hypertension	0.56 (±0.04)	0.70 (±0.03)	0.86 (±0.01)
**53**	Hypertension → PD → Healthy → T2D → Dyslipidemia	0.56 (±0.03)	0.69 (±0.03)	0.85 (±0.01)
**54**	Dyslipidemia → PD → Healthy → Hypertension → T2D	0.56 (±0.03)	0.70 (±0.02)	0.86 (±0.01)
**55**	PD → Dyslipidemia → Healthy → Hypertension → T2D	0.56 (±0.03)	0.70 (±0.02)	0.86 (±0.01)
**56**	Dyslipidemia → PD → Healthy → T2D → Hypertension	0.56 (±0.03)	0.70 (±0.02)	0.86 (±0.01)
**57**	PD → Dyslipidemia → Healthy → T2D → Hypertension	0.56 (±0.03)	0.70 (±0.02)	0.86 (±0.01)
**58**	Dyslipidemia → T2D → PD → Healthy → Hypertension	0.56 (±0.04)	0.70 (±0.03)	0.86 (±0.01)
**59**	T2D → Dyslipidemia → PD → Healthy → Hypertension	0.56 (±0.04)	0.70 (±0.03)	0.86 (±0.01)
**60**	T2D → PD → Dyslipidemia → Healthy → Hypertension	0.56 (±0.04)	0.70 (±0.03)	0.86 (±0.01)

Continued on next page.

Supplementary Table S4. *Continued from previous page.*

#	Label order	Exact match ratio	Hamming score	1 - Hamming loss
**61**	Hypertension → T2D → Healthy → Dyslipidemia → PD	0.56 (±0.03)	0.69 (±0.03)	0.86 (±0.01)
**62**	Hypertension → Healthy → PD → Dyslipidemia → T2D	0.56 (±0.03)	0.69 (±0.03)	0.85 (±0.01)
**63**	Hypertension → T2D → Healthy → PD → Dyslipidemia	0.56 (±0.03)	0.69 (±0.03)	0.86 (±0.01)
**64**	T2D → Hypertension → Healthy → Dyslipidemia → PD	0.56 (±0.03)	0.69 (±0.03)	0.86 (±0.01)
**65**	Hypertension → Healthy → PD → T2D → Dyslipidemia	0.56 (±0.03)	0.69 (±0.03)	0.85 (±0.01)
**66**	Dyslipidemia → Healthy → Hypertension → PD → T2D	0.56 (±0.03)	0.69 (±0.02)	0.85 (±0.01)
**67**	T2D → Hypertension → Healthy → PD → Dyslipidemia	0.56 (±0.03)	0.69 (±0.03)	0.86 (±0.01)
**68**	Hypertension → Healthy → Dyslipidemia → PD → T2D	0.56 (±0.03)	0.69 (±0.03)	0.85 (±0.01)
**69**	Dyslipidemia → Healthy → PD → Hypertension → T2D	0.55 (±0.03)	0.69 (±0.02)	0.85 (±0.01)
**70**	Dyslipidemia → Healthy → PD → T2D → Hypertension	0.55 (±0.03)	0.69 (±0.02)	0.85 (±0.01)
**71**	Hypertension → Healthy → Dyslipidemia → T2D → PD	0.55 (±0.03)	0.69 (±0.03)	0.86 (±0.01)
**72**	Dyslipidemia → Healthy → Hypertension → T2D → PD	0.55 (±0.03)	0.69 (±0.03)	0.86 (±0.01)
**73**	Dyslipidemia → Healthy → T2D → Hypertension → PD	0.55 (±0.03)	0.69 (±0.02)	0.86 (±0.01)
**74**	Hypertension → Healthy → T2D → Dyslipidemia → PD	0.55 (±0.03)	0.68 (±0.03)	0.86 (±0.01)
**75**	Hypertension → Healthy → T2D → PD → Dyslipidemia	0.55 (±0.03)	0.69 (±0.03)	0.86 (±0.01)
**76**	Dyslipidemia → Healthy → T2D → PD → Hypertension	0.55 (±0.03)	0.69 (±0.03)	0.86 (±0.01)
**77**	Dyslipidemia → T2D → Healthy → Hypertension → PD	0.55 (±0.03)	0.69 (±0.03)	0.86 (±0.01)
**78**	T2D → Dyslipidemia → Healthy → Hypertension → PD	0.55 (±0.03)	0.69 (±0.03)	0.86 (±0.01)
**79**	Dyslipidemia → T2D → Healthy → PD → Hypertension	0.55 (±0.04)	0.69 (±0.03)	0.86 (±0.01)
**80**	T2D → Dyslipidemia → Healthy → PD → Hypertension	0.55 (±0.03)	0.69 (±0.03)	0.86 (±0.01)
**81**	PD → T2D → Healthy → Hypertension → Dyslipidemia	0.54 (±0.03)	0.67 (±0.02)	0.85 (±0.01)
**82**	PD → T2D → Healthy → Dyslipidemia → Hypertension	0.54 (±0.03)	0.67 (±0.02)	0.85 (±0.01)
**83**	T2D → PD → Healthy → Hypertension → Dyslipidemia	0.53 (±0.03)	0.67 (±0.02)	0.85 (±0.01)
**84**	T2D → PD → Healthy → Dyslipidemia → Hypertension	0.53 (±0.03)	0.67 (±0.02)	0.85 (±0.01)
**85**	PD → Healthy → Dyslipidemia → Hypertension → T2D	0.53 (±0.03)	0.67 (±0.02)	0.84 (±0.01)
**86**	PD → Healthy → Dyslipidemia → T2D → Hypertension	0.53 (±0.03)	0.67 (±0.02)	0.84 (±0.01)
**87**	PD → Healthy → Hypertension → Dyslipidemia → T2D	0.53 (±0.03)	0.67 (±0.02)	0.84 (±0.01)
**88**	PD → Healthy → T2D → Dyslipidemia → Hypertension	0.53 (±0.03)	0.67 (±0.02)	0.84 (±0.01)
**89**	PD → Healthy → T2D → Hypertension → Dyslipidemia	0.53 (±0.02)	0.66 (±0.02)	0.84 (±0.01)
**90**	PD → Healthy → Hypertension → T2D → Dyslipidemia	0.53 (±0.03)	0.66 (±0.02)	0.84 (±0.01)
**91**	Healthy → Dyslipidemia → Hypertension → PD → T2D	0.53 (±0.03)	0.66 (±0.02)	0.84 (±0.01)
**92**	Healthy → PD → Dyslipidemia → Hypertension → T2D	0.52 (±0.02)	0.66 (±0.02)	0.84 (±0.01)
**93**	Healthy → Hypertension → PD → Dyslipidemia → T2D	0.52 (±0.03)	0.66 (±0.02)	0.84 (±0.01)
**94**	Healthy → PD → Dyslipidemia → T2D → Hypertension	0.52 (±0.02)	0.66 (±0.02)	0.84 (±0.01)
**95**	Healthy → Dyslipidemia → PD → T2D → Hypertension	0.52 (±0.03)	0.66 (±0.02)	0.84 (±0.01)
**96**	Healthy → Dyslipidemia → PD → Hypertension → T2D	0.52 (±0.02)	0.66 (±0.02)	0.84 (±0.01)
**97**	Healthy → PD → Hypertension → Dyslipidemia → T2D	0.52 (±0.02)	0.66 (±0.02)	0.84 (±0.01)
**98**	Healthy → Hypertension → PD → T2D → Dyslipidemia	0.52 (±0.02)	0.66 (±0.02)	0.84 (±0.01)
**99**	Healthy → PD → T2D → Dyslipidemia → Hypertension	0.52 (±0.02)	0.66 (±0.02)	0.84 (±0.01)
**100**	Healthy → Hypertension → Dyslipidemia → PD → T2D	0.52 (±0.02)	0.66 (±0.02)	0.84 (±0.01)
**101**	Healthy → PD → T2D → Hypertension → Dyslipidemia	0.52 (±0.02)	0.66 (±0.02)	0.84 (±0.01)
**102**	T2D → Healthy → Dyslipidemia → Hypertension → PD	0.52 (±0.03)	0.66 (±0.02)	0.85 (±0.01)
**103**	Healthy → PD → Hypertension → T2D → Dyslipidemia	0.52 (±0.02)	0.66 (±0.02)	0.84 (±0.01)
**104**	Healthy → Dyslipidemia → Hypertension → T2D → PD	0.52 (±0.03)	0.66 (±0.02)	0.85 (±0.01)
**105**	Healthy → Dyslipidemia → T2D → Hypertension → PD	0.52 (±0.03)	0.66 (±0.02)	0.85 (±0.01)
**106**	T2D → Healthy → PD → Dyslipidemia → Hypertension	0.52 (±0.03)	0.66 (±0.02)	0.85 (±0.01)
**107**	T2D → Healthy → PD → Hypertension → Dyslipidemia	0.52 (±0.03)	0.66 (±0.03)	0.85 (±0.01)
**108**	T2D → Healthy → Dyslipidemia → PD → Hypertension	0.52 (±0.03)	0.66 (±0.02)	0.85 (±0.01)
**109**	T2D → Healthy → Hypertension → Dyslipidemia → PD	0.52 (±0.03)	0.66 (±0.03)	0.85 (±0.01)
**110**	Healthy → T2D → Dyslipidemia → Hypertension → PD	0.52 (±0.02)	0.66 (±0.02)	0.85 (±0.01)
**111**	Healthy → Dyslipidemia → T2D → PD → Hypertension	0.52 (±0.03)	0.66 (±0.02)	0.85 (±0.01)
**112**	Healthy → Hypertension → Dyslipidemia → T2D → PD	0.52 (±0.02)	0.66 (±0.02)	0.85 (±0.01)
**113**	T2D → Healthy → Hypertension → PD → Dyslipidemia	0.52 (±0.03)	0.66 (±0.03)	0.85 (±0.01)
**114**	Healthy → T2D → PD → Dyslipidemia → Hypertension	0.52 (±0.03)	0.66 (±0.02)	0.85 (±0.01)
**115**	Healthy → T2D → Dyslipidemia → PD → Hypertension	0.52 (±0.03)	0.66 (±0.02)	0.85 (±0.01)
**116**	Healthy → Hypertension → T2D → Dyslipidemia → PD	0.52 (±0.02)	0.66 (±0.02)	0.85 (±0.01)
**117**	Healthy → Hypertension → T2D → PD → Dyslipidemia	0.52 (±0.03)	0.66 (±0.02)	0.85 (±0.01)
**118**	Healthy → T2D → PD → Hypertension → Dyslipidemia	0.52 (±0.02)	0.66 (±0.02)	0.85 (±0.01)
**119**	Healthy → T2D → Hypertension → Dyslipidemia → PD	0.52 (±0.03)	0.66 (±0.02)	0.85 (±0.01)
**120**	Healthy → T2D → Hypertension → PD → Dyslipidemia	0.52 (±0.03)	0.66 (±0.02)	0.85 (±0.01)
**121**	Non-chained model (label order not applicable)	0.50 (±0.02)	0.64 (±0.02)	0.86 (±0.01)

Input	True label ($\mathbf{y}_i$)	Model output ($\hat{\mathbf{y}}_i$)	Exact match ratio	Hamming score	1 - Hamming loss
$\mathbf{x}_1$	$[1\,0\,0\,0\,0]$	$[0\,1\,0\,0\,0]$	0.00	$0/2 = 0.00$	$3/5 = 0.60$
$\mathbf{x}_2$	$[1\,0\,0\,0\,0]$	$[1\,1\,0\,0\,0]$	0.00	$1/2 = 0.50$	$4/5 = 0.80$
$\mathbf{x}_3$	$[1\,1\,0\,0\,0]$	$[1\,0\,1\,0\,0]$	0.00	$1/3 = 0.33$	$3/5 = 0.60$
$\mathbf{x}_4$	$[1\,1\,0\,0\,0]$	$[1\,1\,1\,0\,0]$	0.00	$2/3 = 0.67$	$4/5 = 0.80$
$\mathbf{x}_5$	$[1\,1\,1\,0\,0]$	$[1\,1\,1\,1\,0]$	0.00	$3/4 = 0.75$	$4/5 = 0.80$
$\mathbf{x}_6$	$[1\,1\,1\,1\,0]$	$[1\,1\,1\,1\,0]$	1.00	$4/4 = 1.00$	$5/5 = 1.00$
**Average**			**0.17**	**0.54**	**0.77**

Supplementary Table S5. Example of evaluating multilabel classification metrics, related to STAR Methods. The exact match ratio, Hamming score, and Hamming loss metrics were computed and provided as examples to demonstrate the accuracy assessment between predictions and true labels. $\mathbf{x}_i$ represents an input sample, $\mathbf{y}_i$ is a five-dimensional vector of the true labels for the sample, and $\hat{\mathbf{y}}_i$ is the vector of predicted labels. Erroneous label predictions are highlighted in red.
